# Supplementary figures and images for: Stock market forecasting research based on GA-WOA-LSTM
Source: PLoS One. 2025 Aug 27;20(8):e0330324. doi: 10.1371/journal.pone.0330324 (PMC12385415; doi:10.1371/journal.pone.0330324)

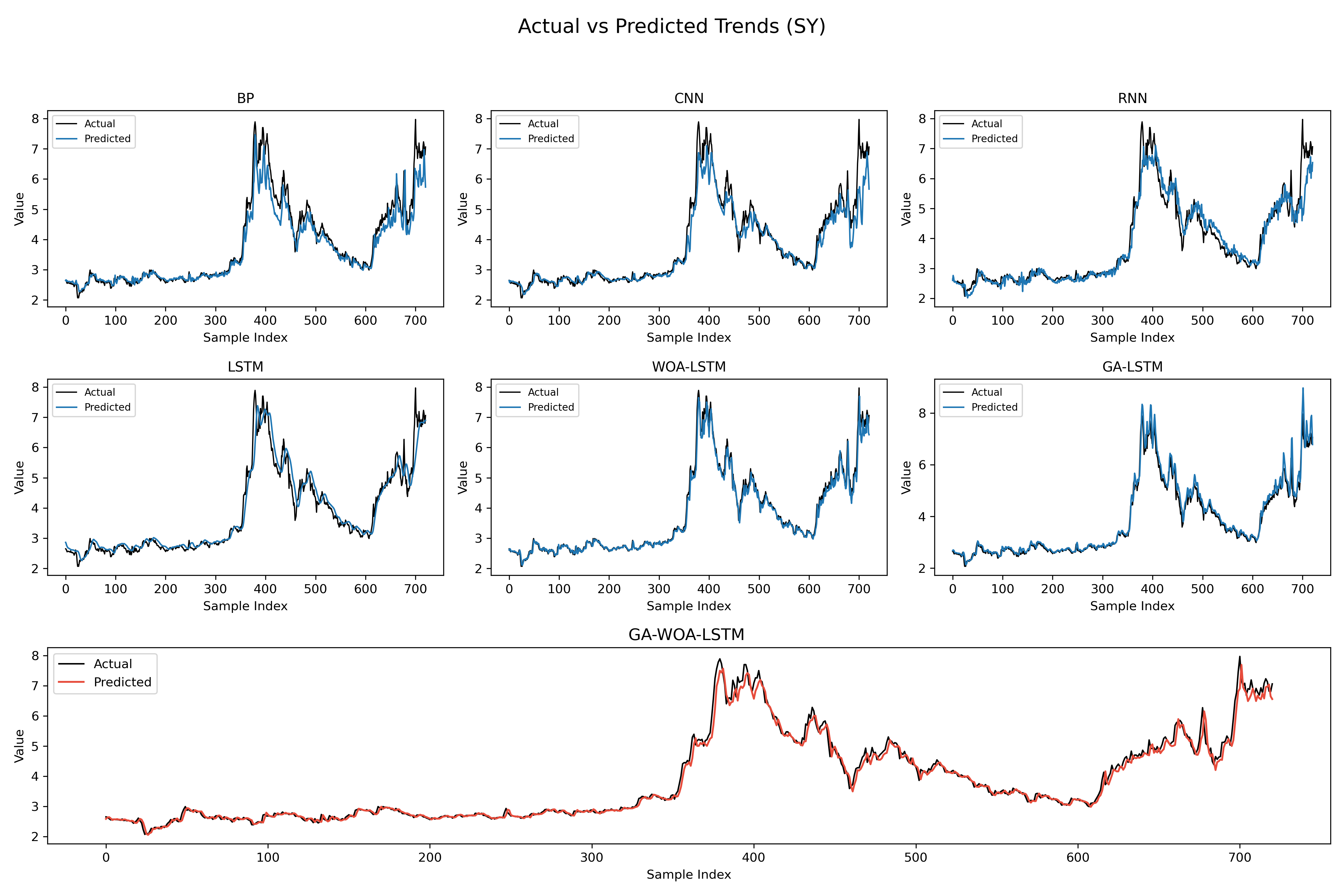

Supplement: S1 File — (ZIP) [file pone.0330324.s001.zip › Paper Model/LH/LOTUS HOLDINGS-600186.SS/figures/actual_vs_predicted_7models_highlighted.png]

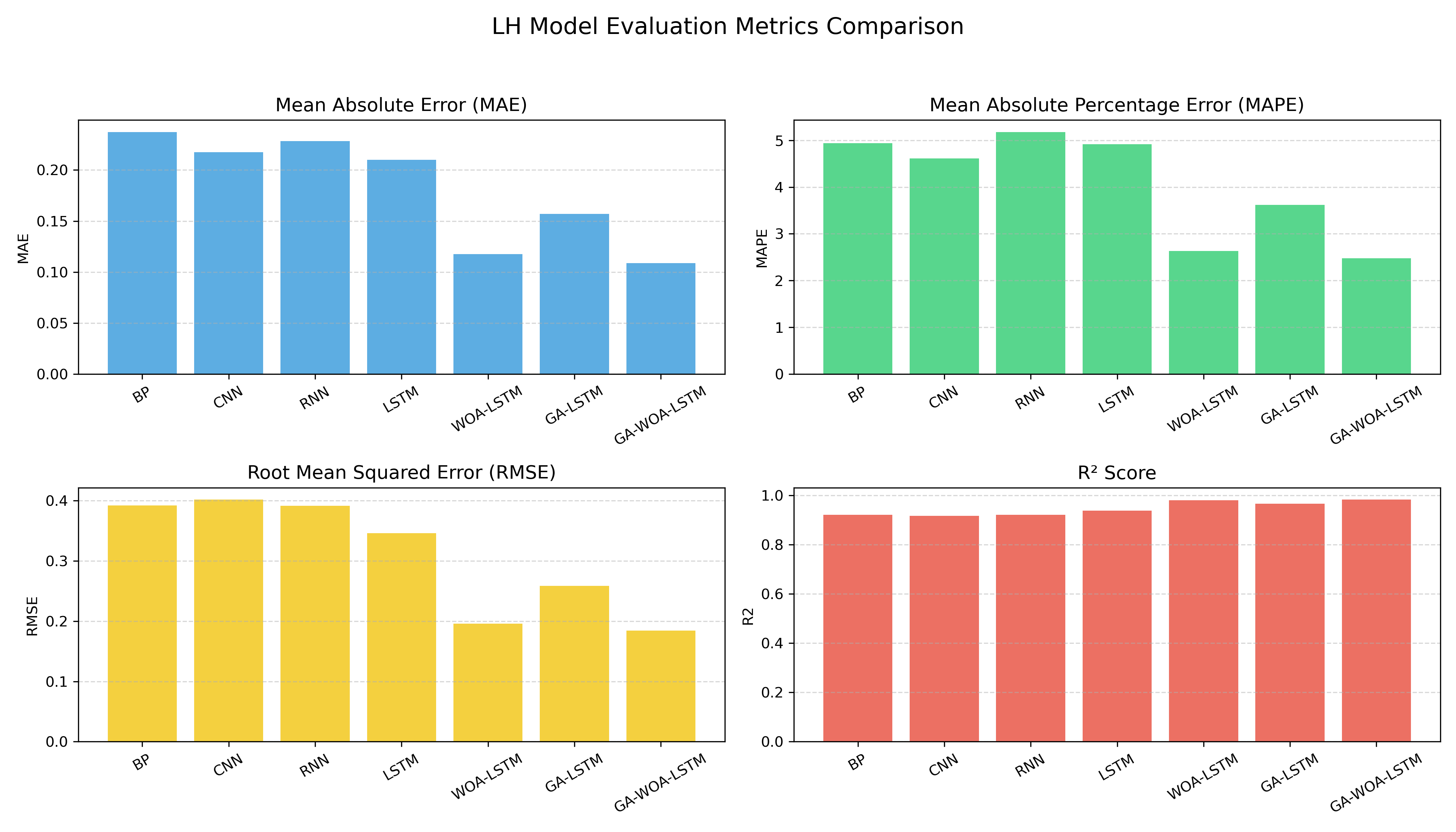

Supplement: S1 File — (ZIP) [file pone.0330324.s001.zip › Paper Model/LH/LOTUS HOLDINGS-600186.SS/figures/test_model_metrics_comparison_grid.png]

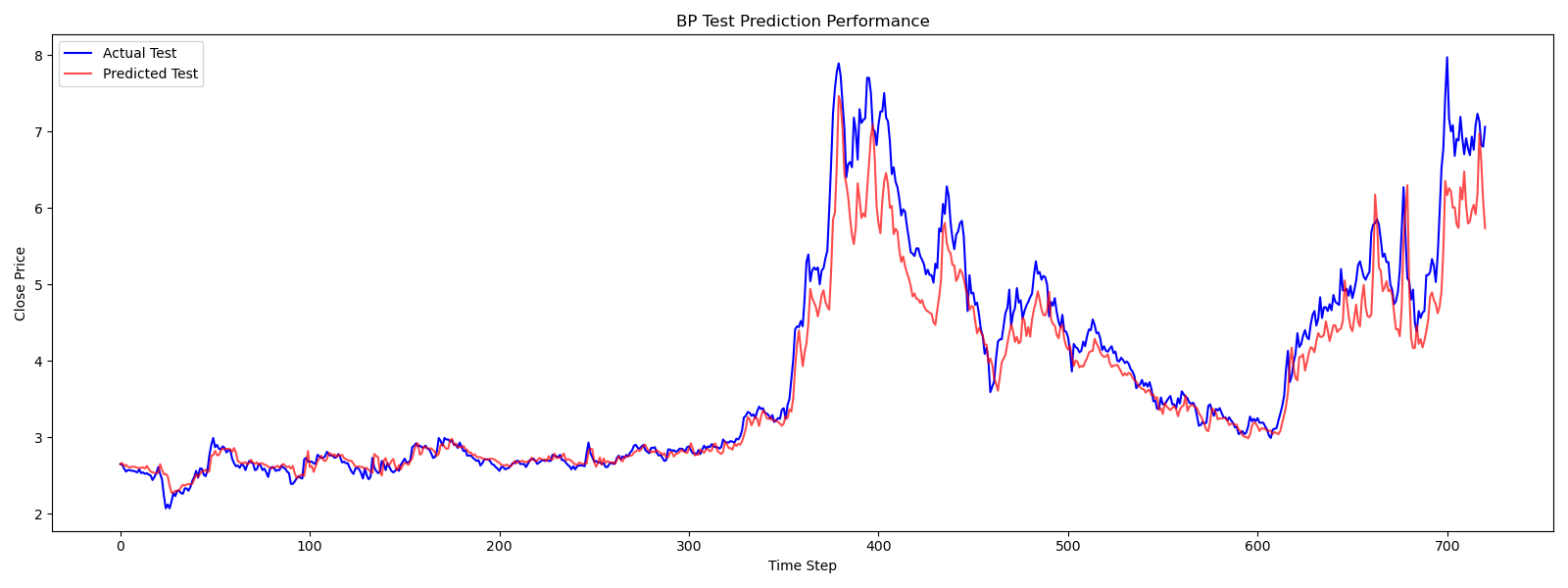

Supplement: S1 File — (ZIP) [file pone.0330324.s001.zip › Paper Model/LH/LOTUS HOLDINGS-600186.SS/GA-WOA-LSTM/figures/BP_test_fit_plot.png]

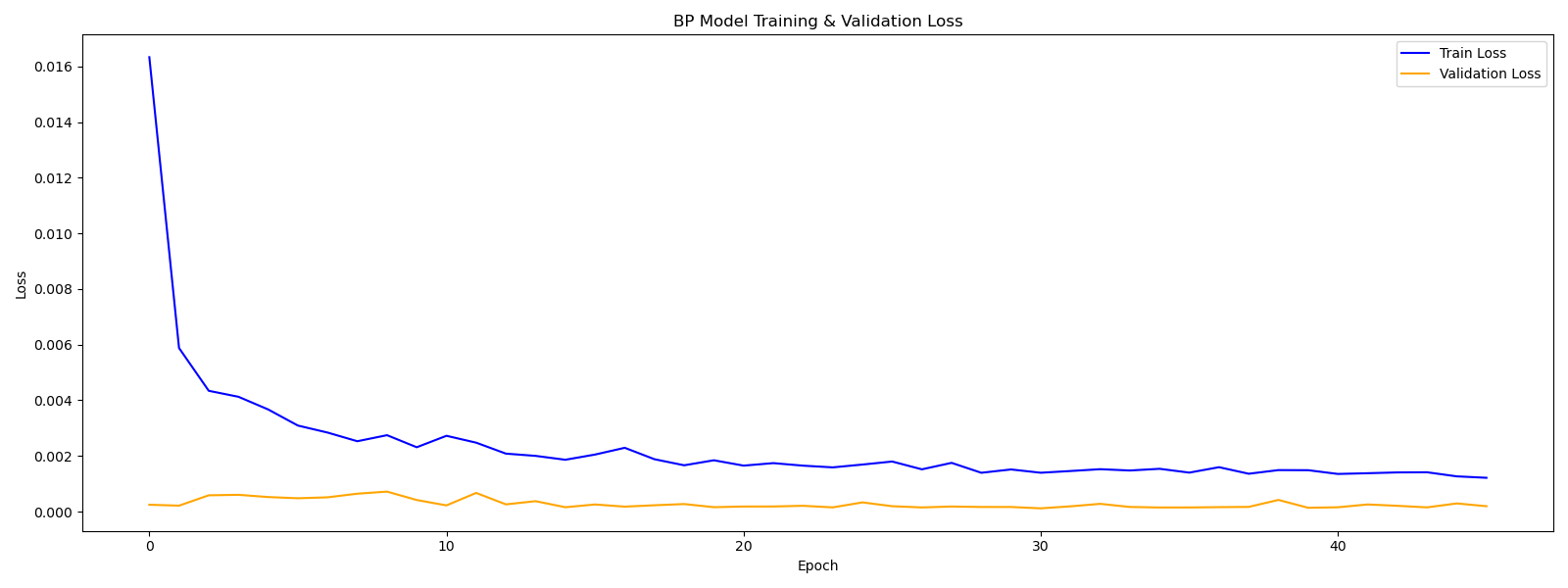

Supplement: S1 File — (ZIP) [file pone.0330324.s001.zip › Paper Model/LH/LOTUS HOLDINGS-600186.SS/GA-WOA-LSTM/figures/BP_training_loss.png]

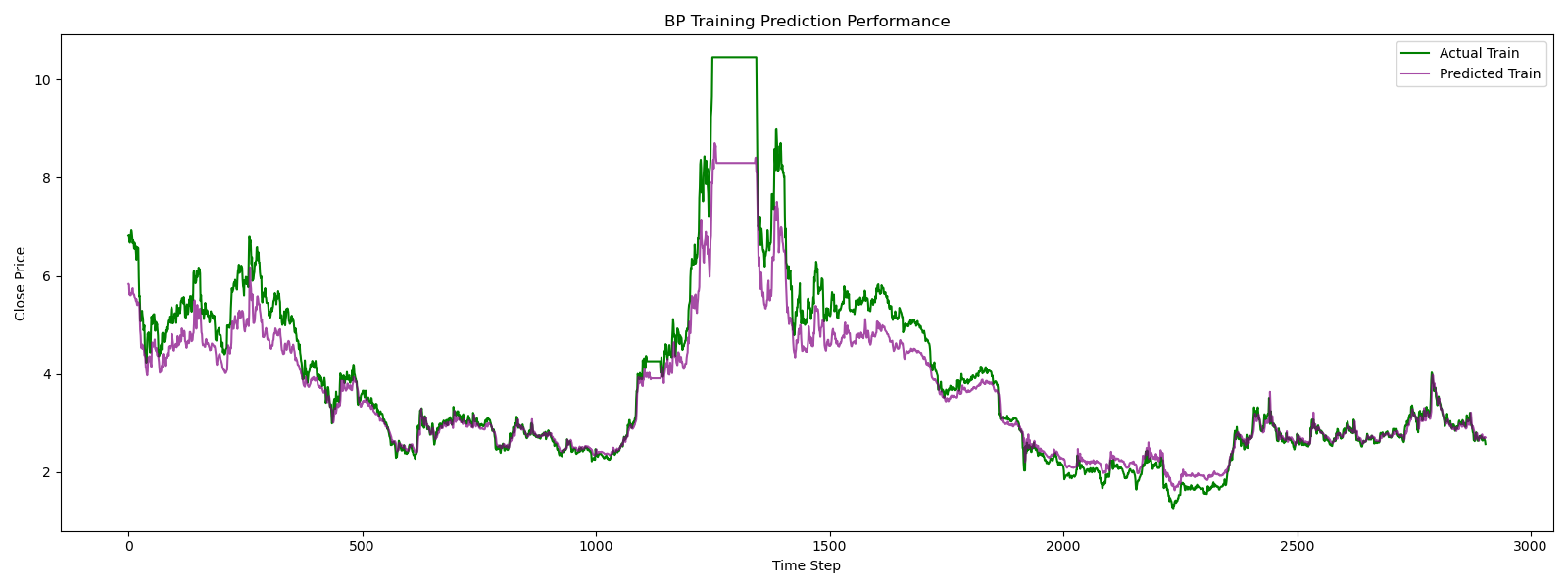

Supplement: S1 File — (ZIP) [file pone.0330324.s001.zip › Paper Model/LH/LOTUS HOLDINGS-600186.SS/GA-WOA-LSTM/figures/BP_train_fit_plot.png]

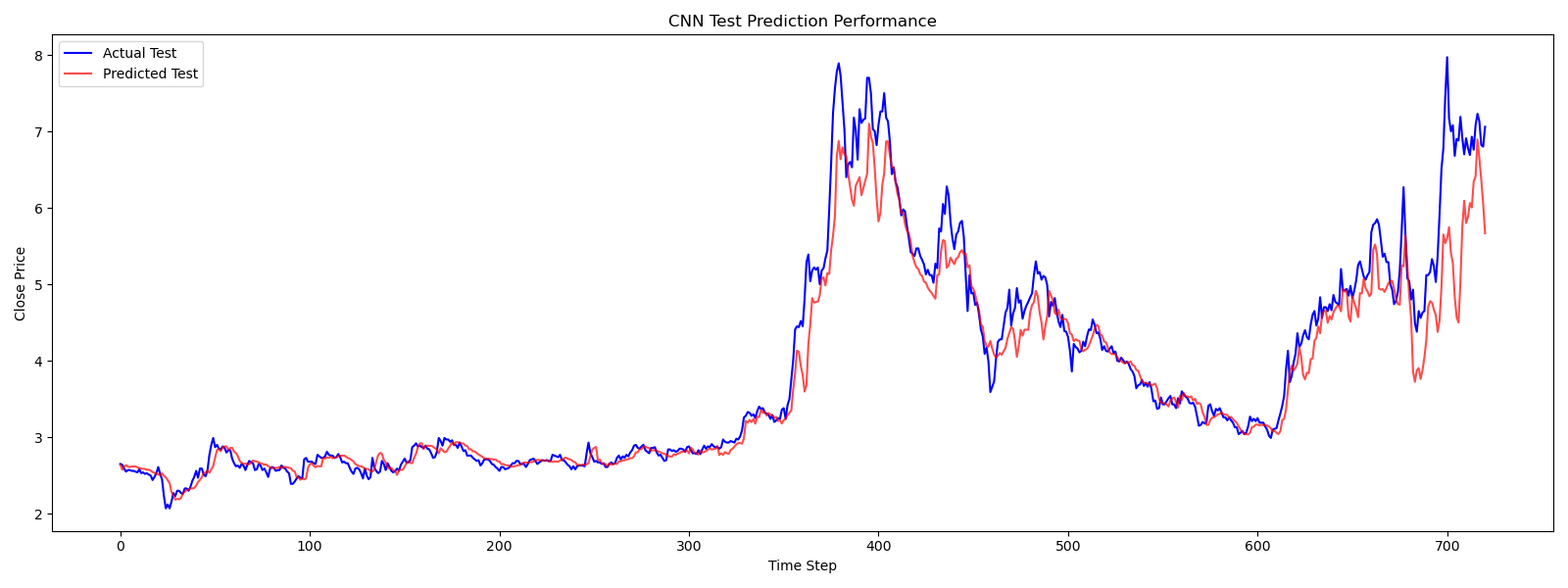

Supplement: S1 File — (ZIP) [file pone.0330324.s001.zip › Paper Model/LH/LOTUS HOLDINGS-600186.SS/GA-WOA-LSTM/figures/CNN_test_fit_plot.png]

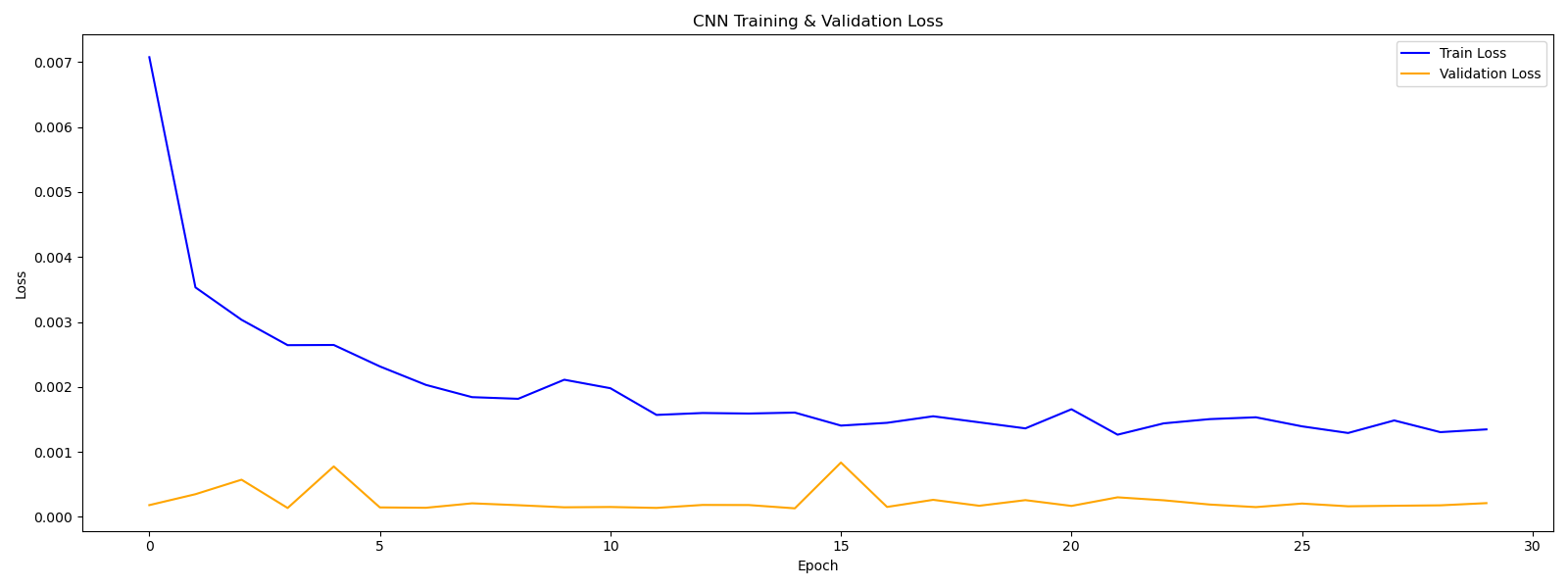

Supplement: S1 File — (ZIP) [file pone.0330324.s001.zip › Paper Model/LH/LOTUS HOLDINGS-600186.SS/GA-WOA-LSTM/figures/CNN_training_loss.png]

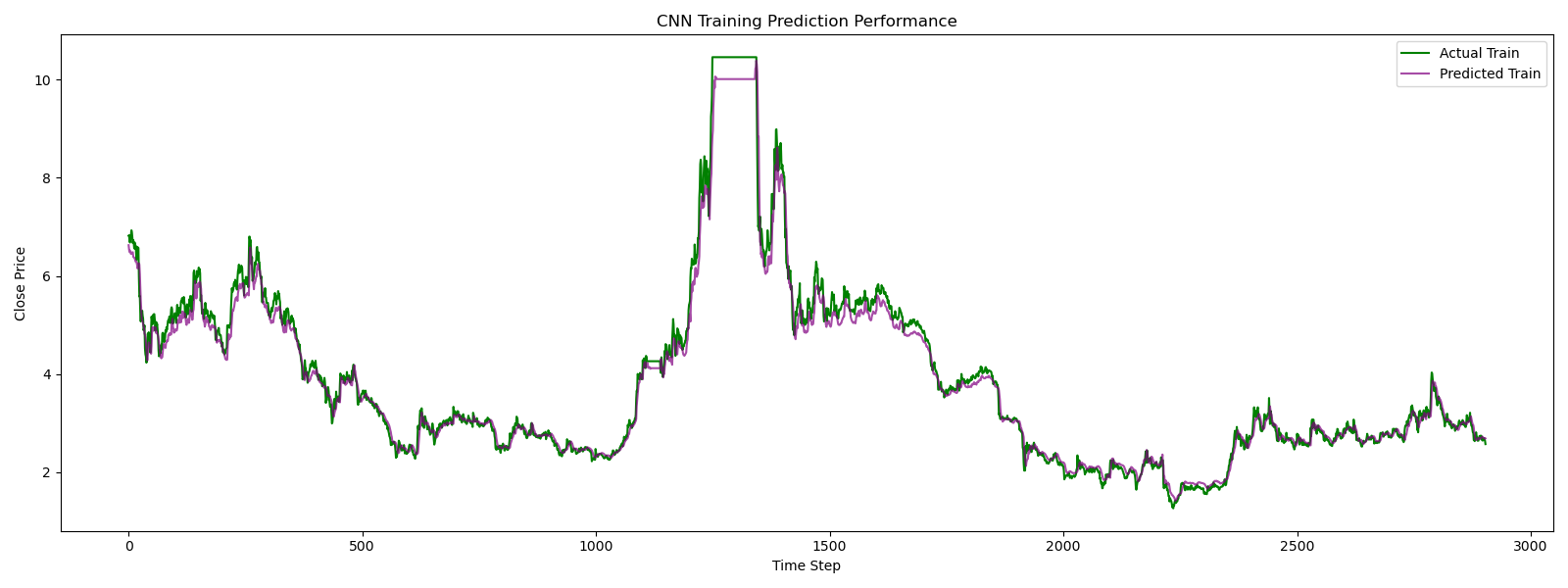

Supplement: S1 File — (ZIP) [file pone.0330324.s001.zip › Paper Model/LH/LOTUS HOLDINGS-600186.SS/GA-WOA-LSTM/figures/CNN_train_fit_plot.png]

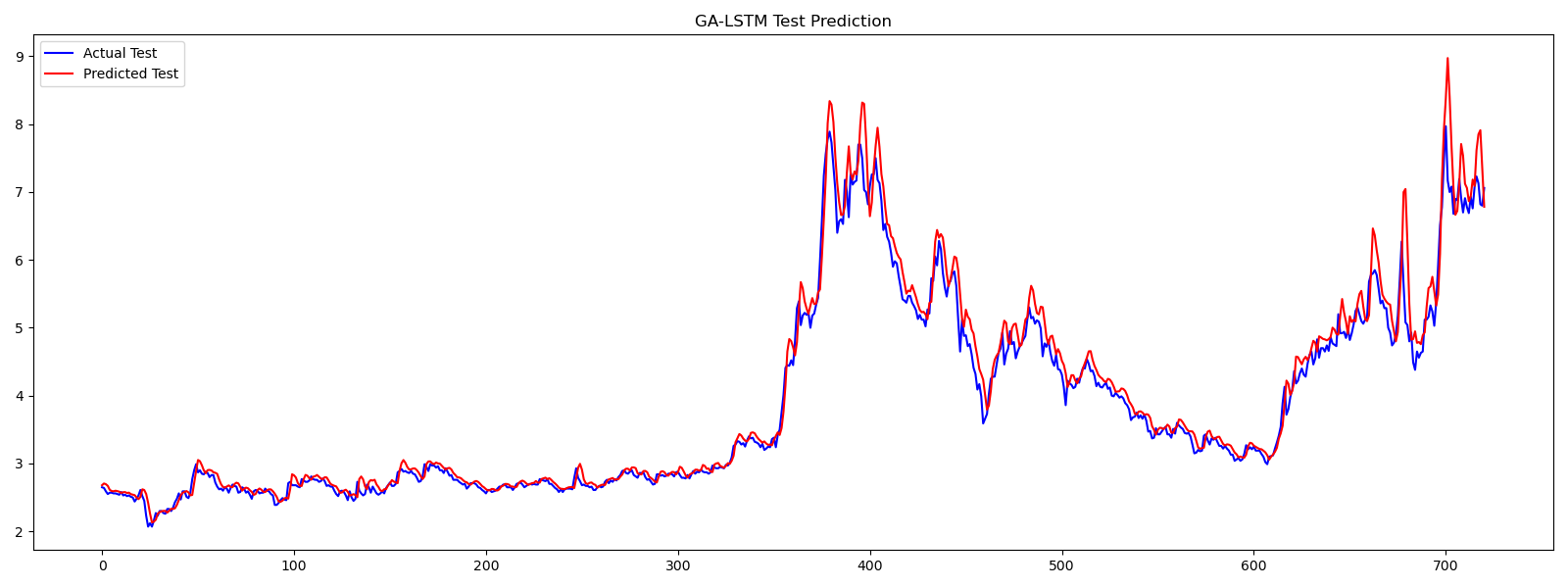

Supplement: S1 File — (ZIP) [file pone.0330324.s001.zip › Paper Model/LH/LOTUS HOLDINGS-600186.SS/GA-WOA-LSTM/figures/GA_LSTM_test_fit_plot.png]

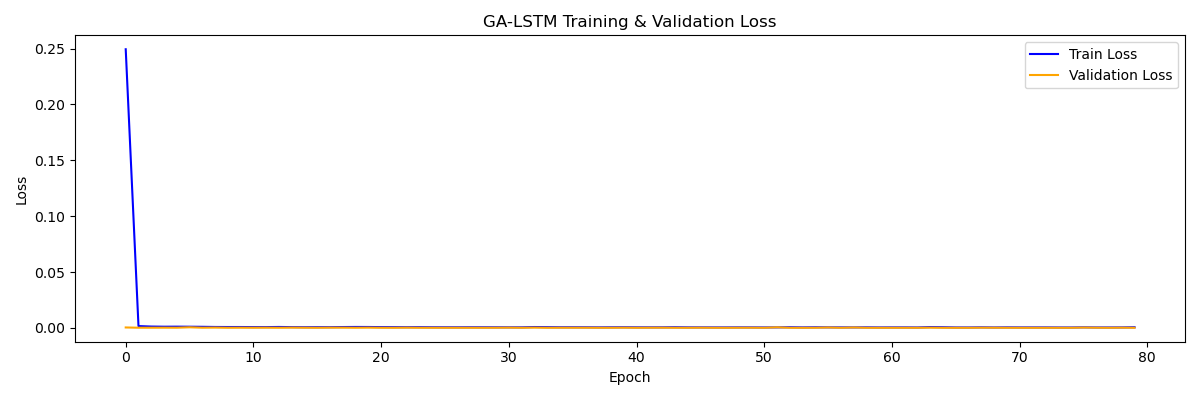

Supplement: S1 File — (ZIP) [file pone.0330324.s001.zip › Paper Model/LH/LOTUS HOLDINGS-600186.SS/GA-WOA-LSTM/figures/GA_LSTM_training_loss.png]

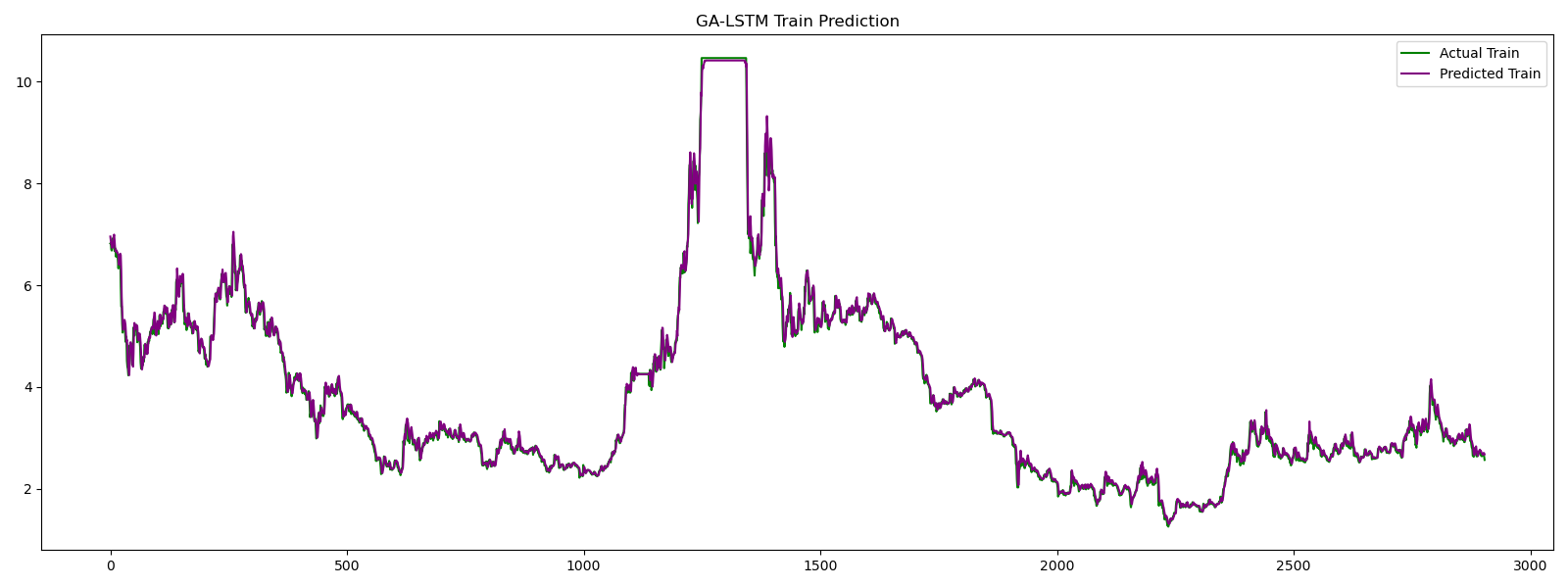

Supplement: S1 File — (ZIP) [file pone.0330324.s001.zip › Paper Model/LH/LOTUS HOLDINGS-600186.SS/GA-WOA-LSTM/figures/GA_LSTM_train_fit_plot.png]

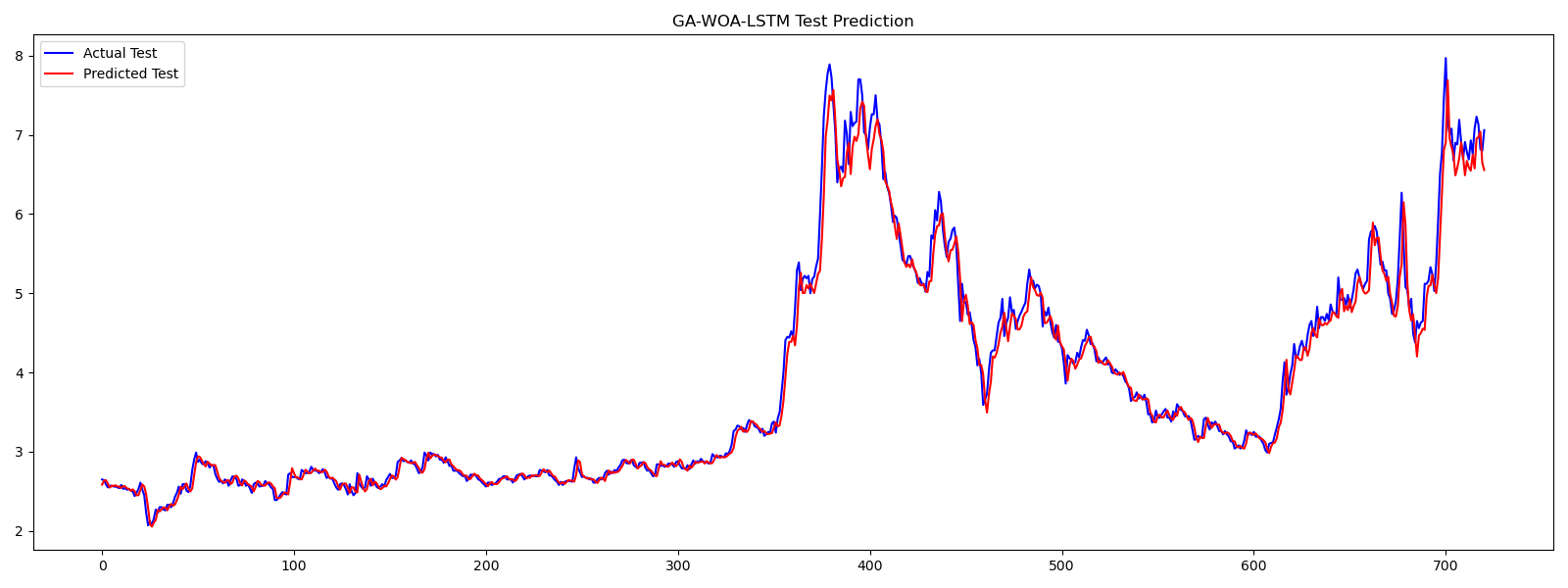

Supplement: S1 File — (ZIP) [file pone.0330324.s001.zip › Paper Model/LH/LOTUS HOLDINGS-600186.SS/GA-WOA-LSTM/figures/GA_WOA_LSTM_test_fit_plot.png]

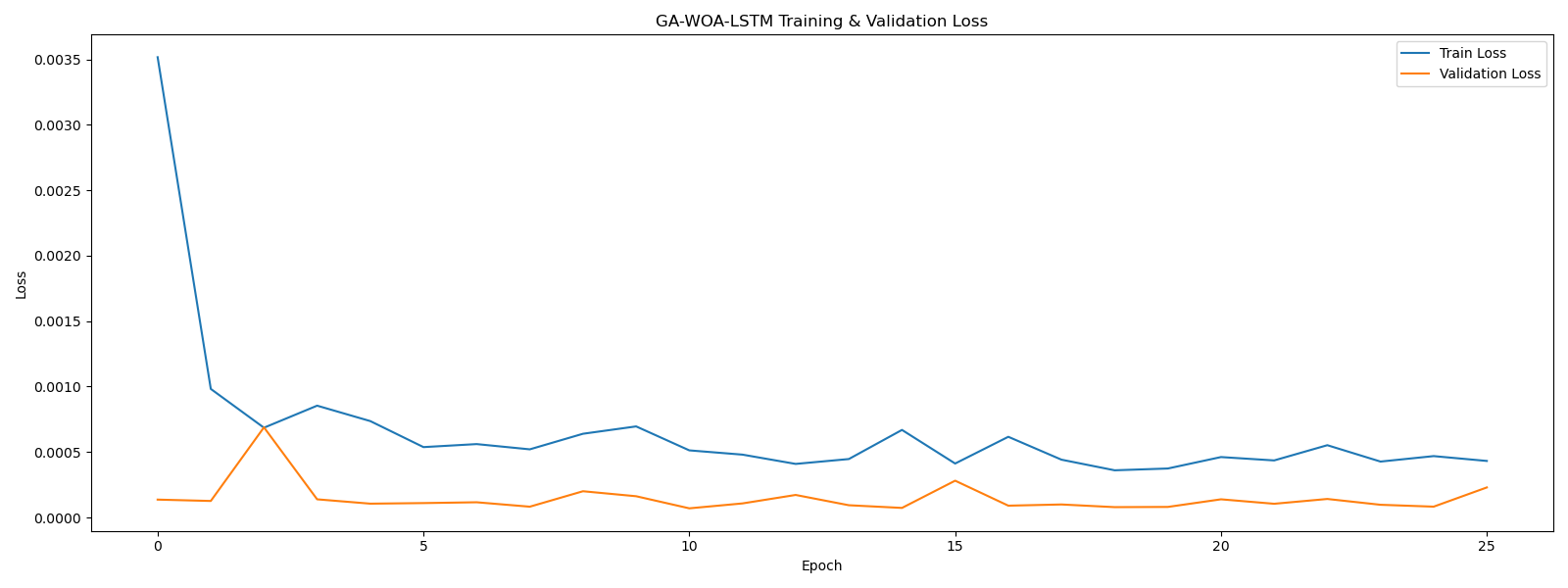

Supplement: S1 File — (ZIP) [file pone.0330324.s001.zip › Paper Model/LH/LOTUS HOLDINGS-600186.SS/GA-WOA-LSTM/figures/GA_WOA_LSTM_training_loss.png]

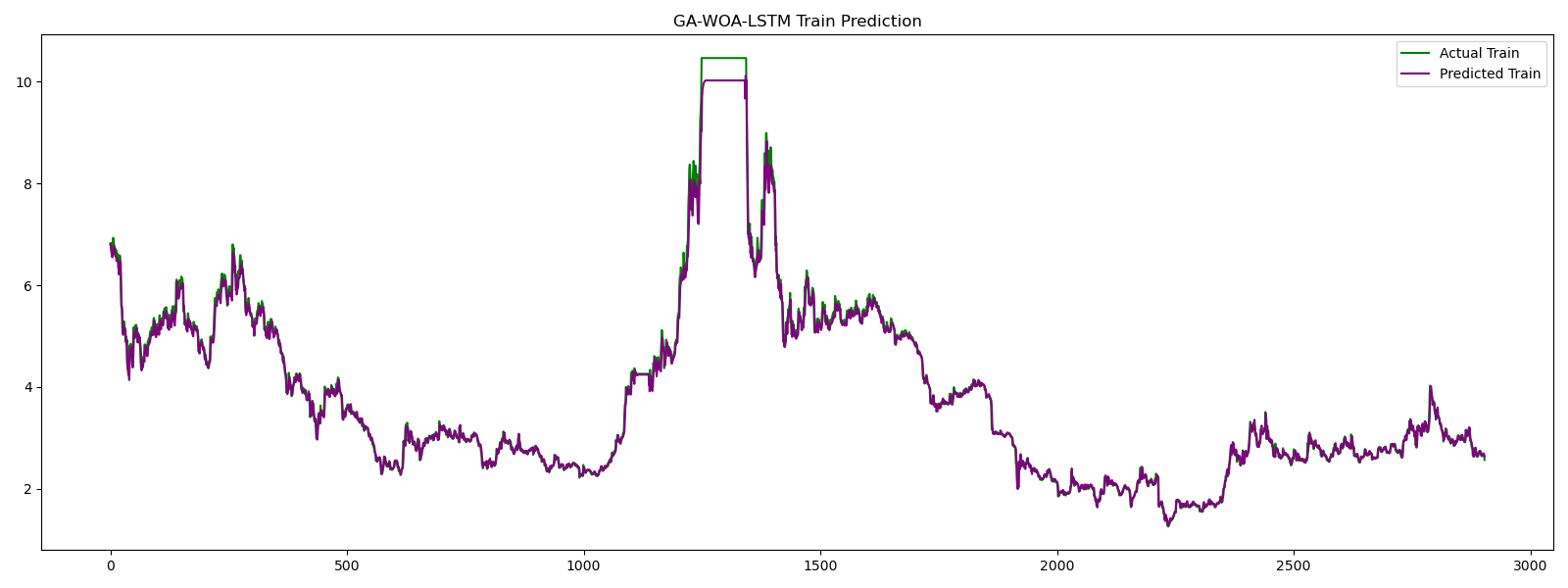

Supplement: S1 File — (ZIP) [file pone.0330324.s001.zip › Paper Model/LH/LOTUS HOLDINGS-600186.SS/GA-WOA-LSTM/figures/GA_WOA_LSTM_train_fit_plot.png]

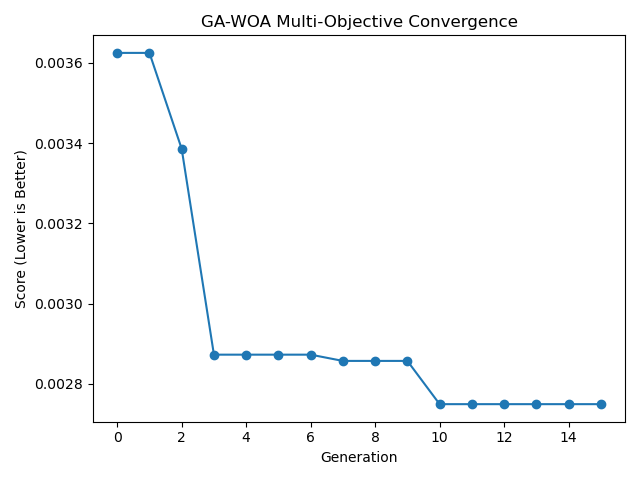

Supplement: S1 File — (ZIP) [file pone.0330324.s001.zip › Paper Model/LH/LOTUS HOLDINGS-600186.SS/GA-WOA-LSTM/figures/GA_WOA_multiobj_convergence_curve.png]

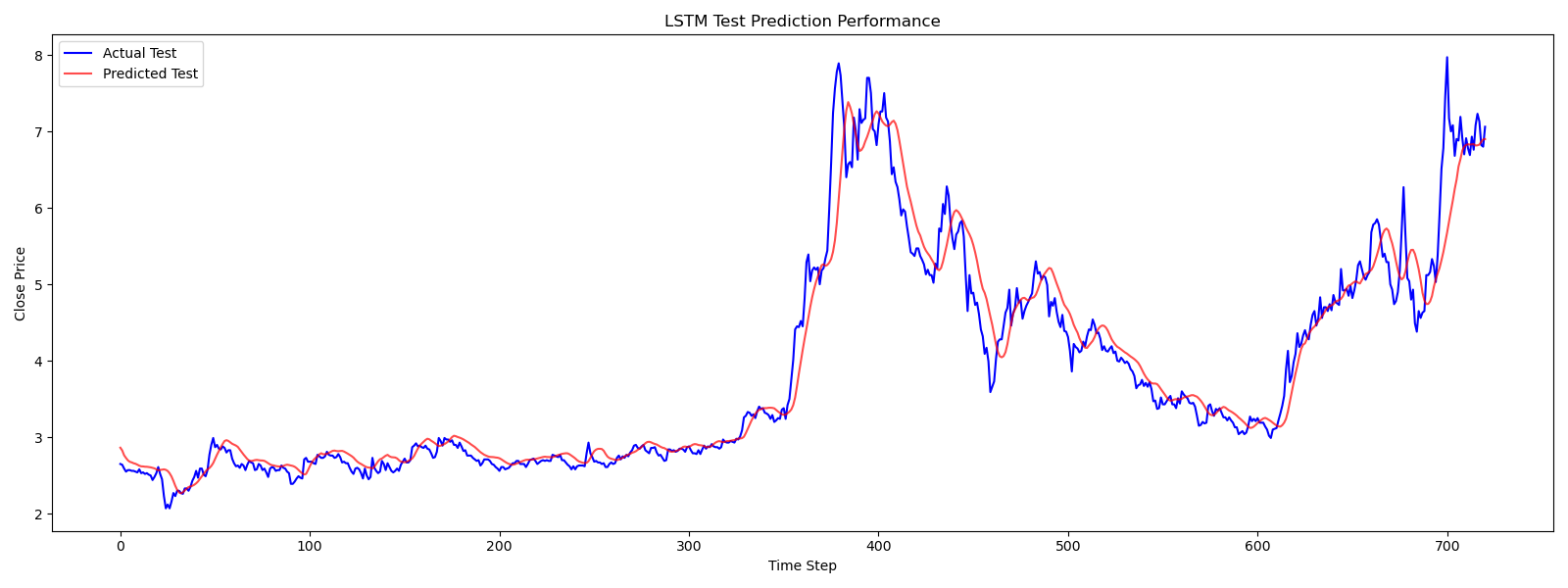

Supplement: S1 File — (ZIP) [file pone.0330324.s001.zip › Paper Model/LH/LOTUS HOLDINGS-600186.SS/GA-WOA-LSTM/figures/LSTM_test_fit_plot.png]

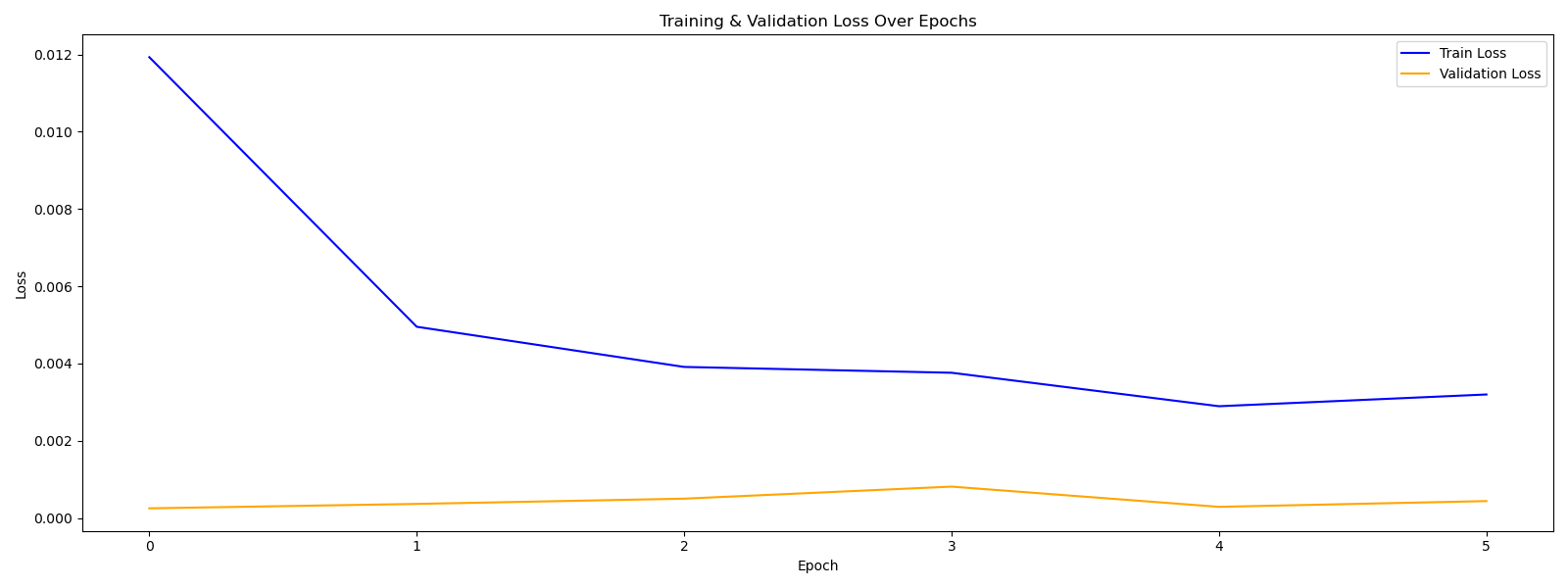

Supplement: S1 File — (ZIP) [file pone.0330324.s001.zip › Paper Model/LH/LOTUS HOLDINGS-600186.SS/GA-WOA-LSTM/figures/LSTM_training_loss.png]

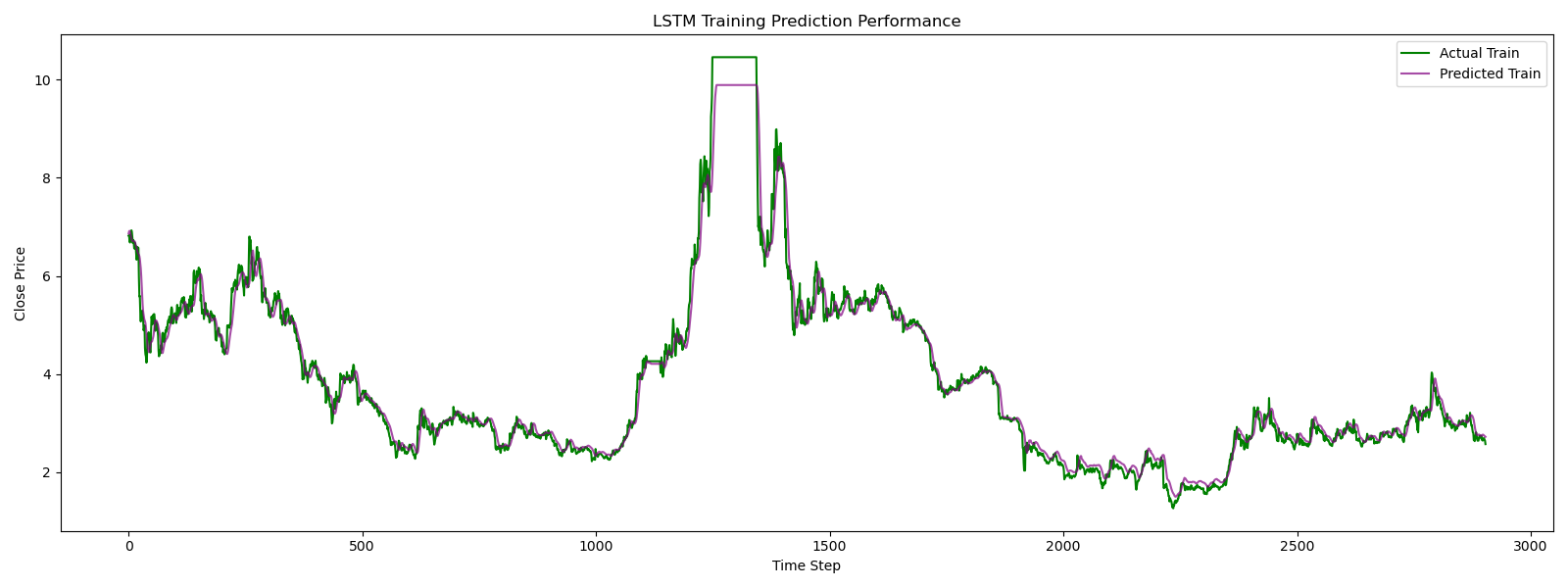

Supplement: S1 File — (ZIP) [file pone.0330324.s001.zip › Paper Model/LH/LOTUS HOLDINGS-600186.SS/GA-WOA-LSTM/figures/LSTM_train_fit_plot.png]

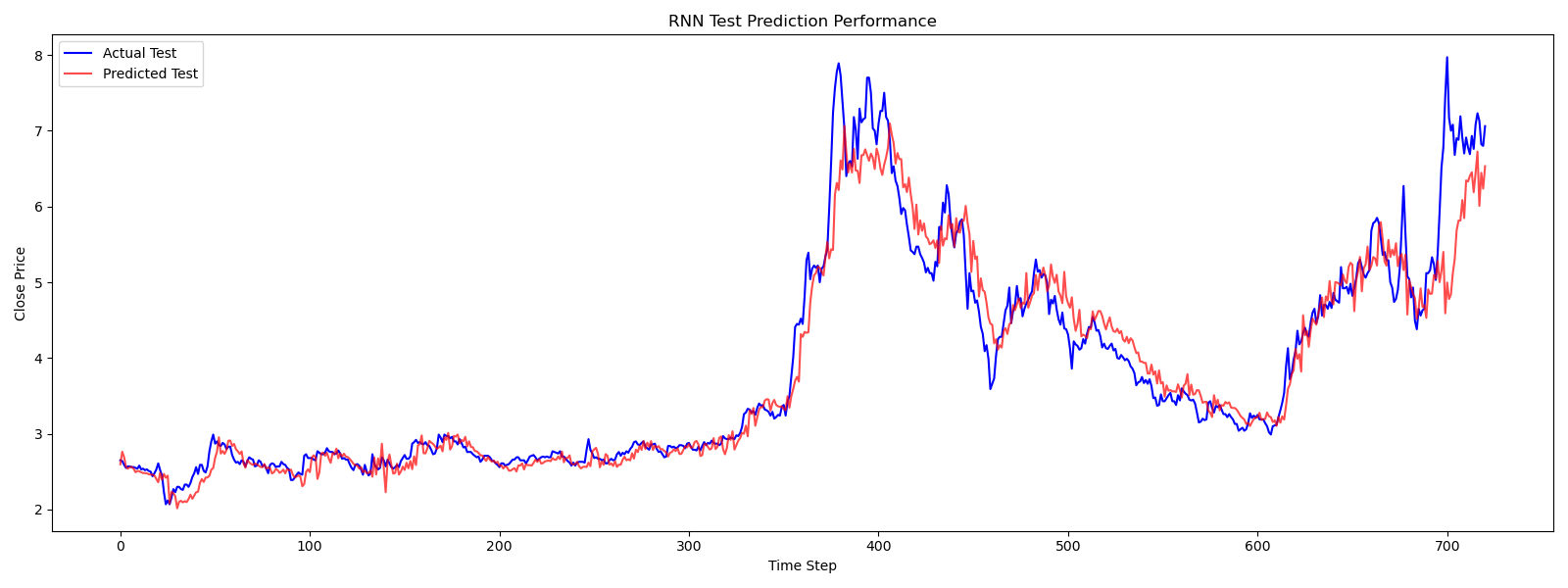

Supplement: S1 File — (ZIP) [file pone.0330324.s001.zip › Paper Model/LH/LOTUS HOLDINGS-600186.SS/GA-WOA-LSTM/figures/RNN_test_fit_plot.png]

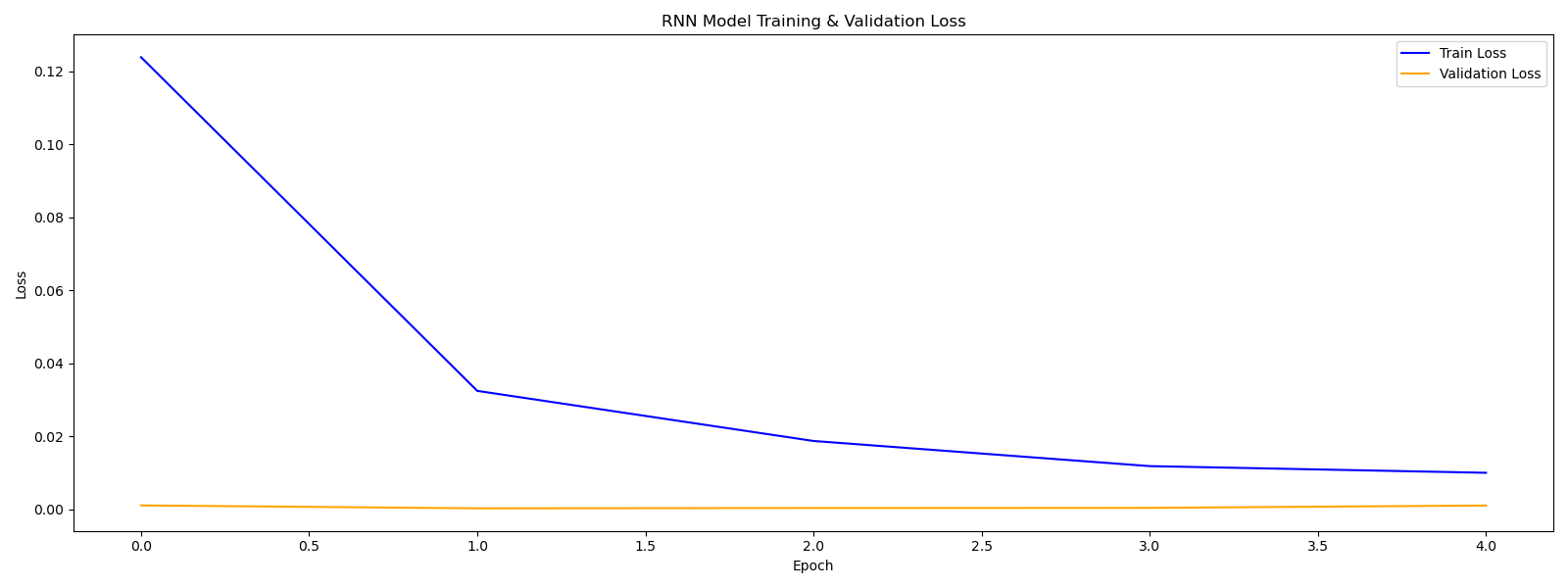

Supplement: S1 File — (ZIP) [file pone.0330324.s001.zip › Paper Model/LH/LOTUS HOLDINGS-600186.SS/GA-WOA-LSTM/figures/RNN_training_loss.png]

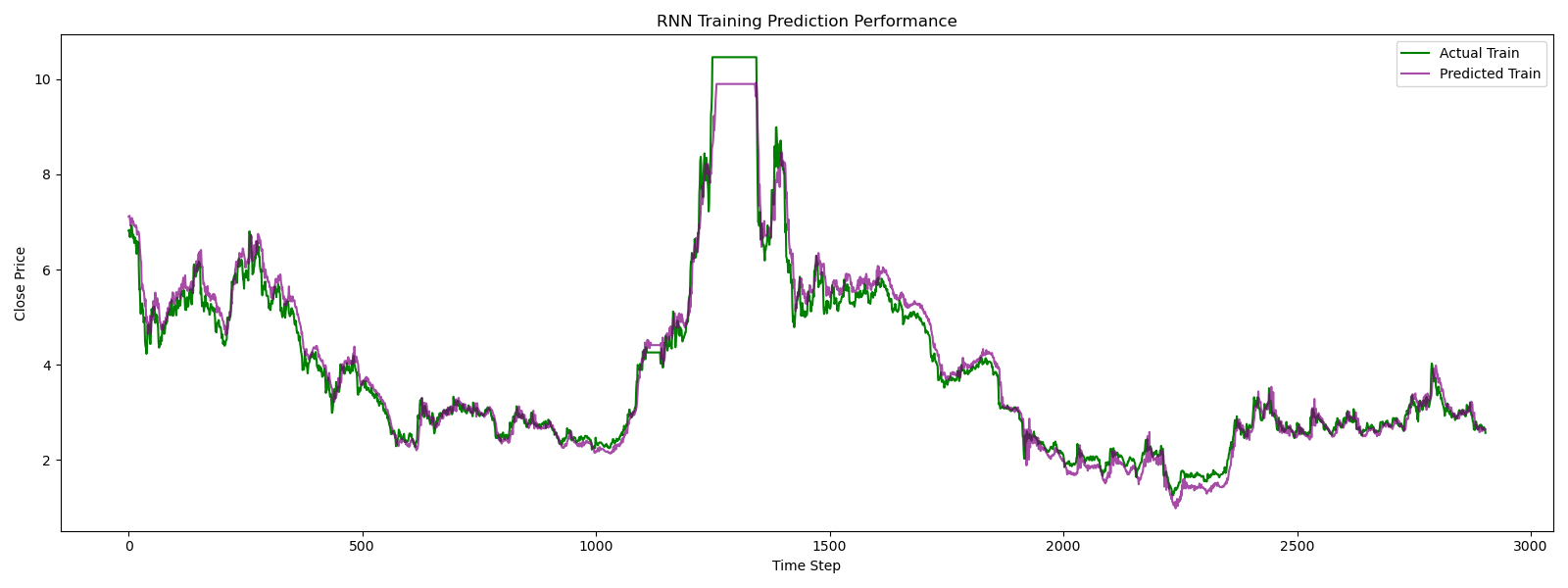

Supplement: S1 File — (ZIP) [file pone.0330324.s001.zip › Paper Model/LH/LOTUS HOLDINGS-600186.SS/GA-WOA-LSTM/figures/RNN_train_fit_plot.png]

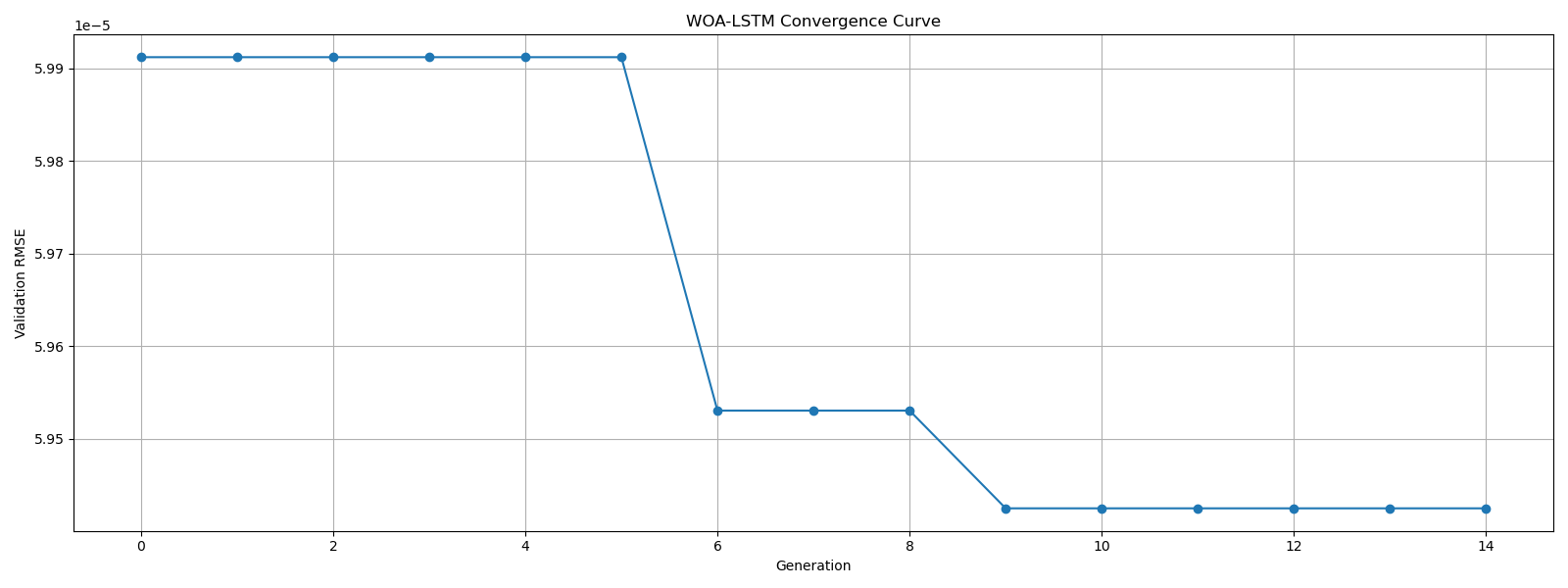

Supplement: S1 File — (ZIP) [file pone.0330324.s001.zip › Paper Model/LH/LOTUS HOLDINGS-600186.SS/GA-WOA-LSTM/figures/WOA_LSTM_convergence_curve.png]

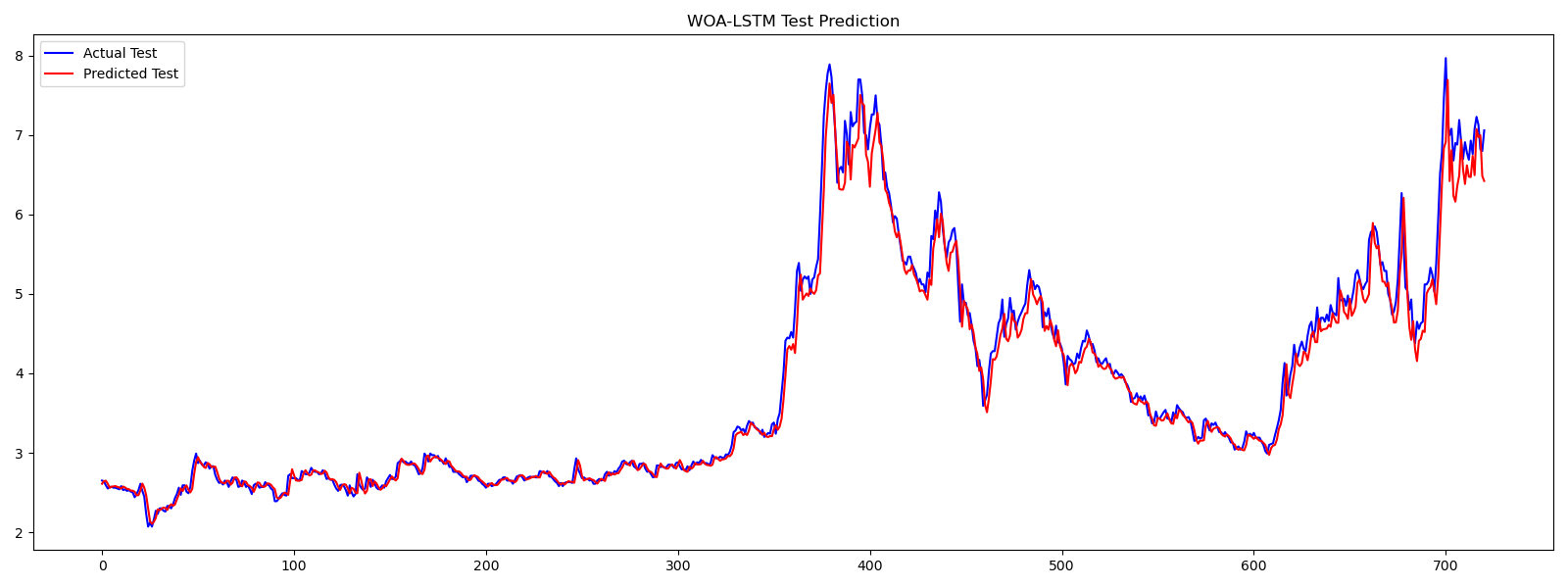

Supplement: S1 File — (ZIP) [file pone.0330324.s001.zip › Paper Model/LH/LOTUS HOLDINGS-600186.SS/GA-WOA-LSTM/figures/WOA_LSTM_test_fit_plot.png]

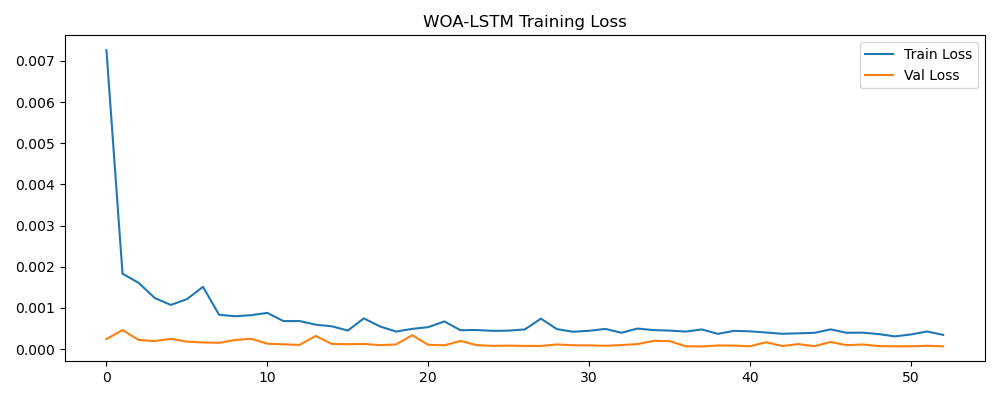

Supplement: S1 File — (ZIP) [file pone.0330324.s001.zip › Paper Model/LH/LOTUS HOLDINGS-600186.SS/GA-WOA-LSTM/figures/WOA_LSTM_training_loss.png]

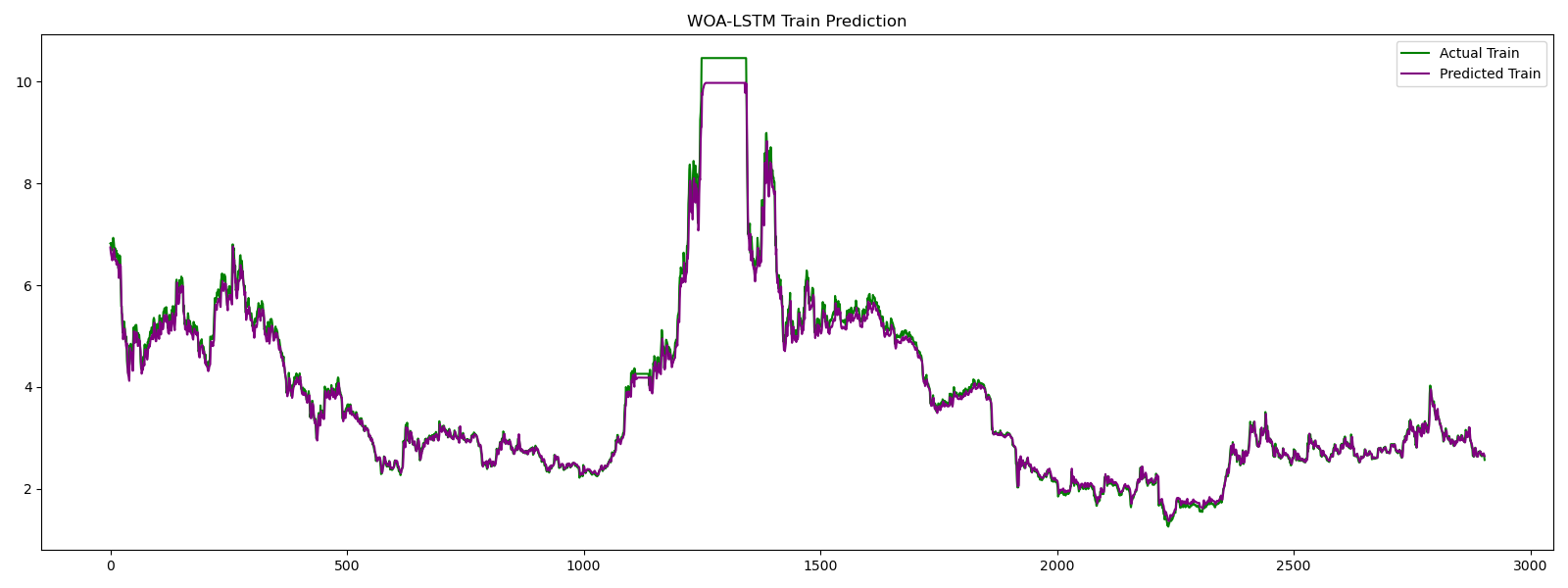

Supplement: S1 File — (ZIP) [file pone.0330324.s001.zip › Paper Model/LH/LOTUS HOLDINGS-600186.SS/GA-WOA-LSTM/figures/WOA_LSTM_train_fit_plot.png]

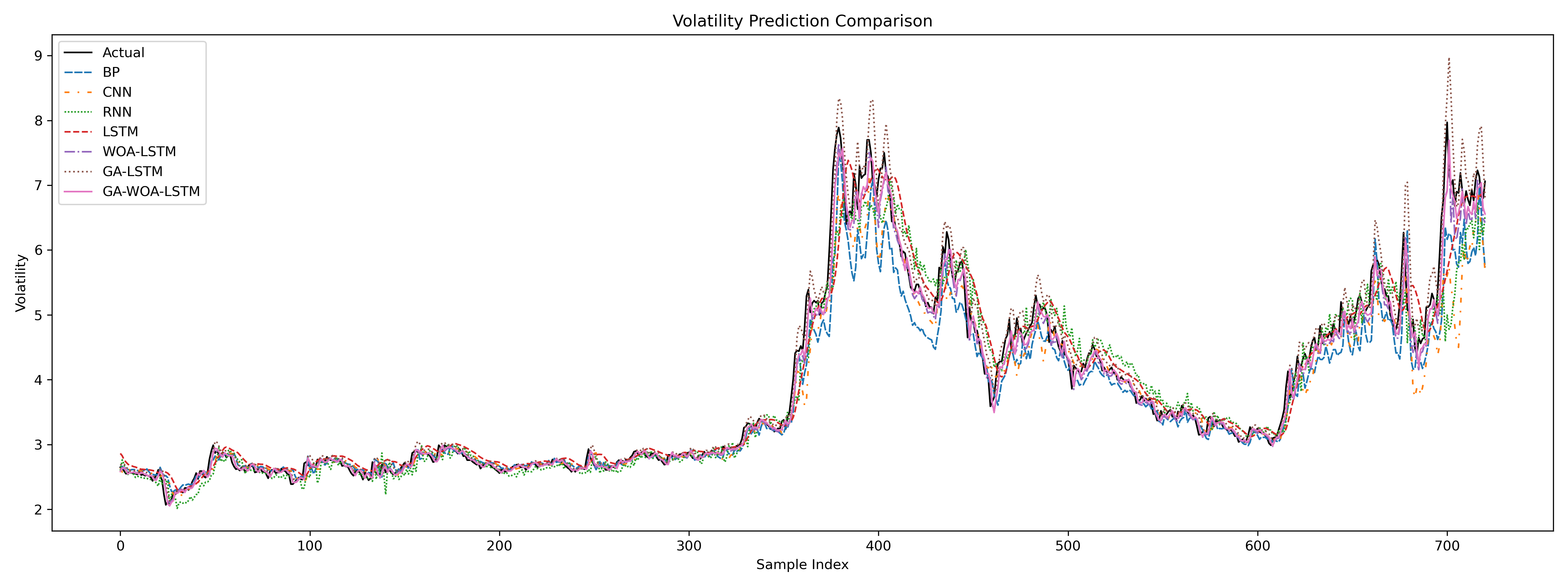

Supplement: S1 File — (ZIP) [file pone.0330324.s001.zip › Paper Model/LH/LOTUS HOLDINGS-600186.SS/test_model_comparison_plot.png]

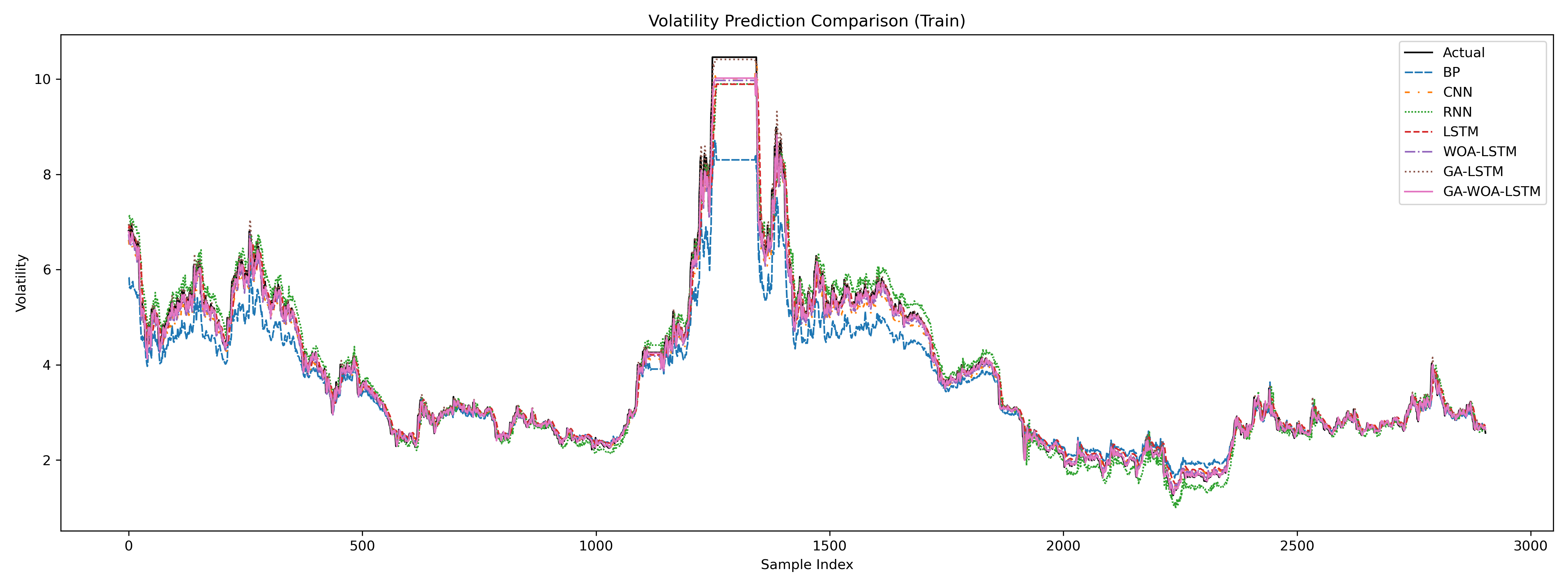

Supplement: S1 File — (ZIP) [file pone.0330324.s001.zip › Paper Model/LH/LOTUS HOLDINGS-600186.SS/train_model_comparison_plot.png]

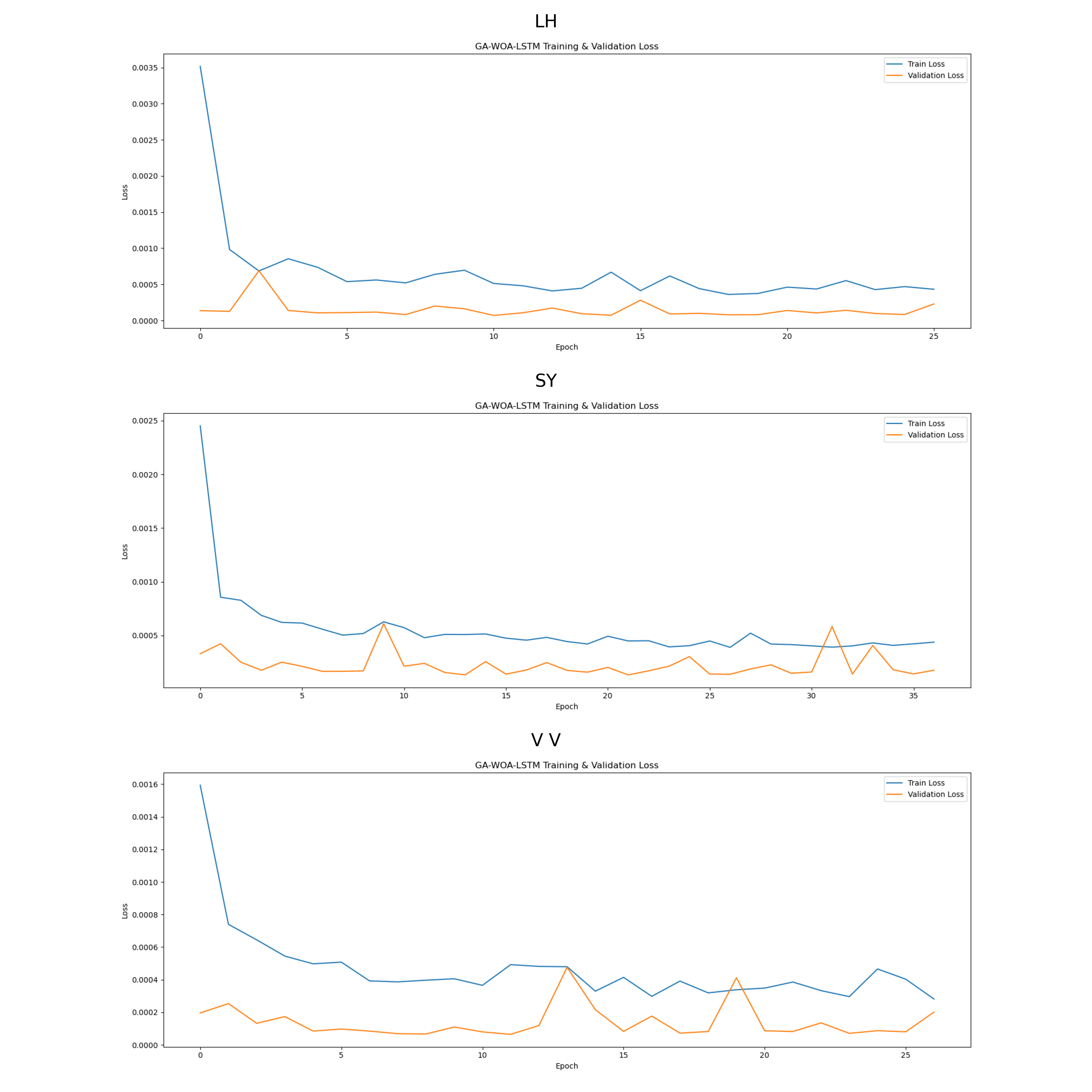

Supplement: S1 File — (ZIP) [file pone.0330324.s001.zip › Paper Model/merged_vertical_image.png]

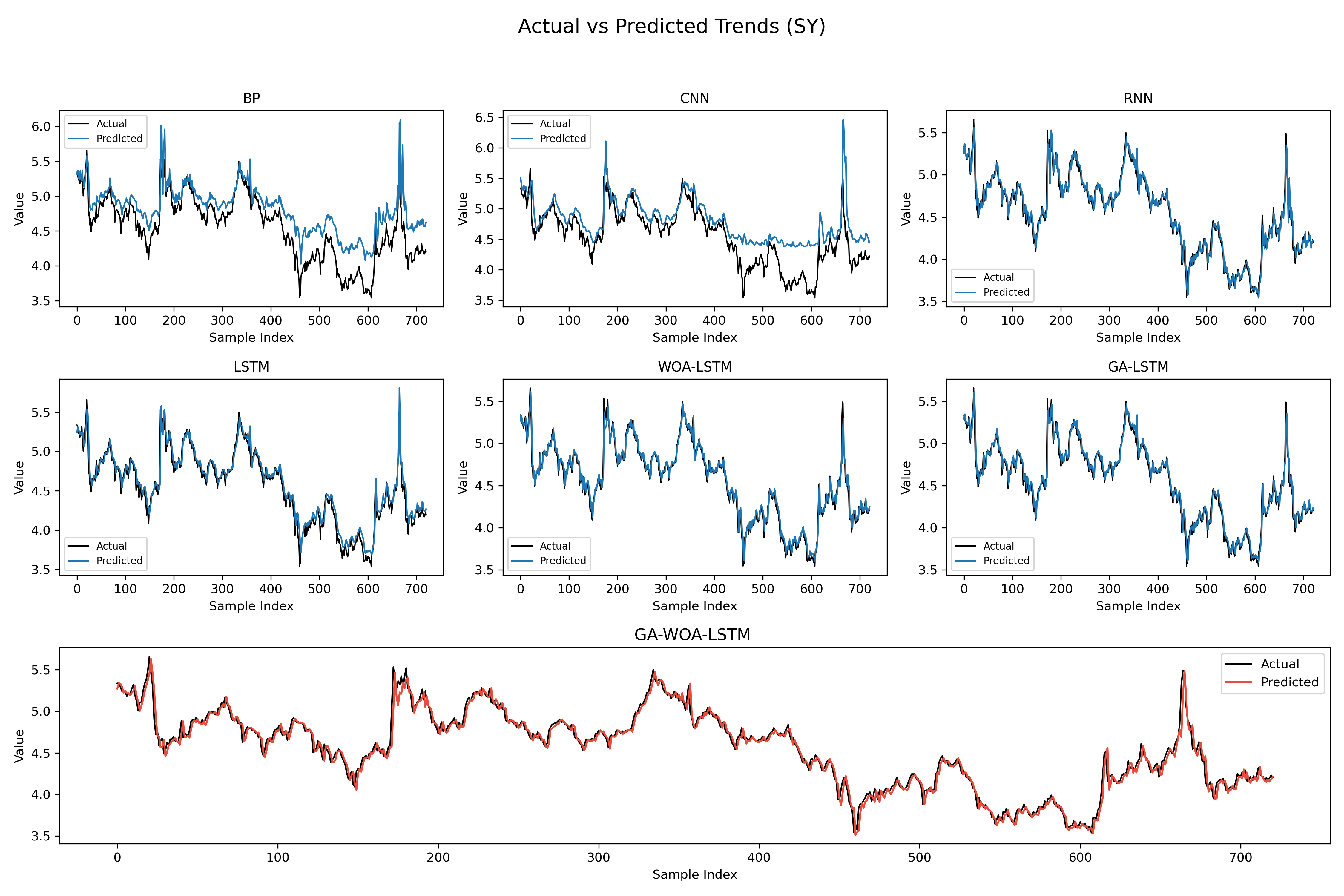

Supplement: S1 File — (ZIP) [file pone.0330324.s001.zip › Paper Model/SY/SANYUAN-600429.SS/figures/actual_vs_predicted_7models_highlighted.png]

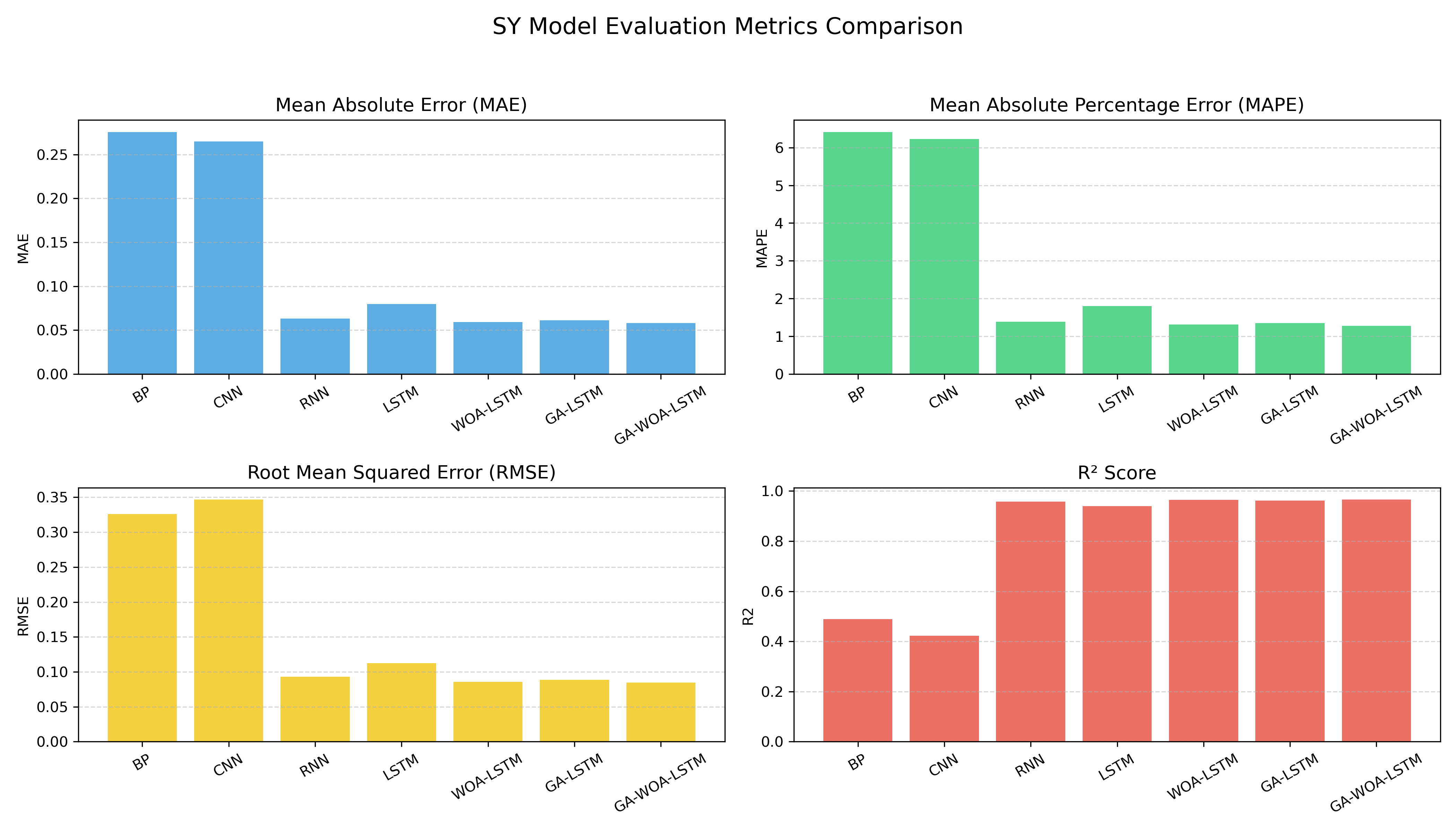

Supplement: S1 File — (ZIP) [file pone.0330324.s001.zip › Paper Model/SY/SANYUAN-600429.SS/figures/test_model_metrics_comparison_grid.png]

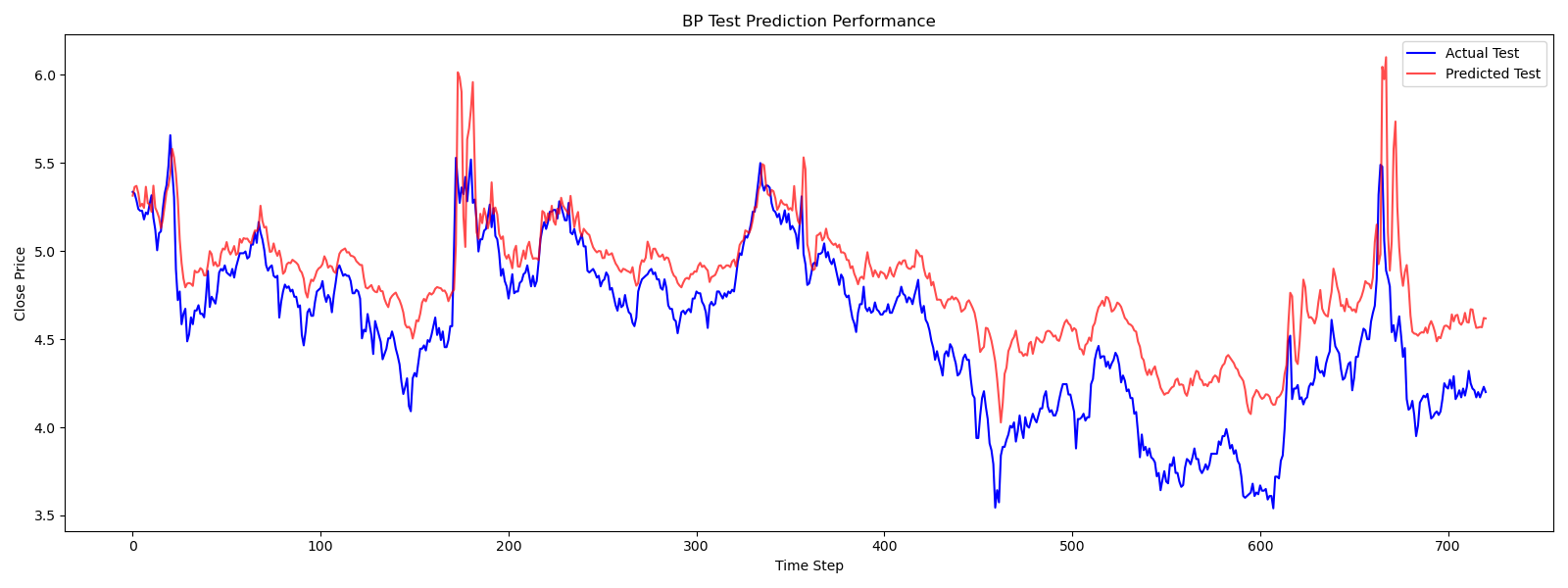

Supplement: S1 File — (ZIP) [file pone.0330324.s001.zip › Paper Model/SY/SANYUAN-600429.SS/GA-WOA-LSTM/figures/BP_test_fit_plot.png]

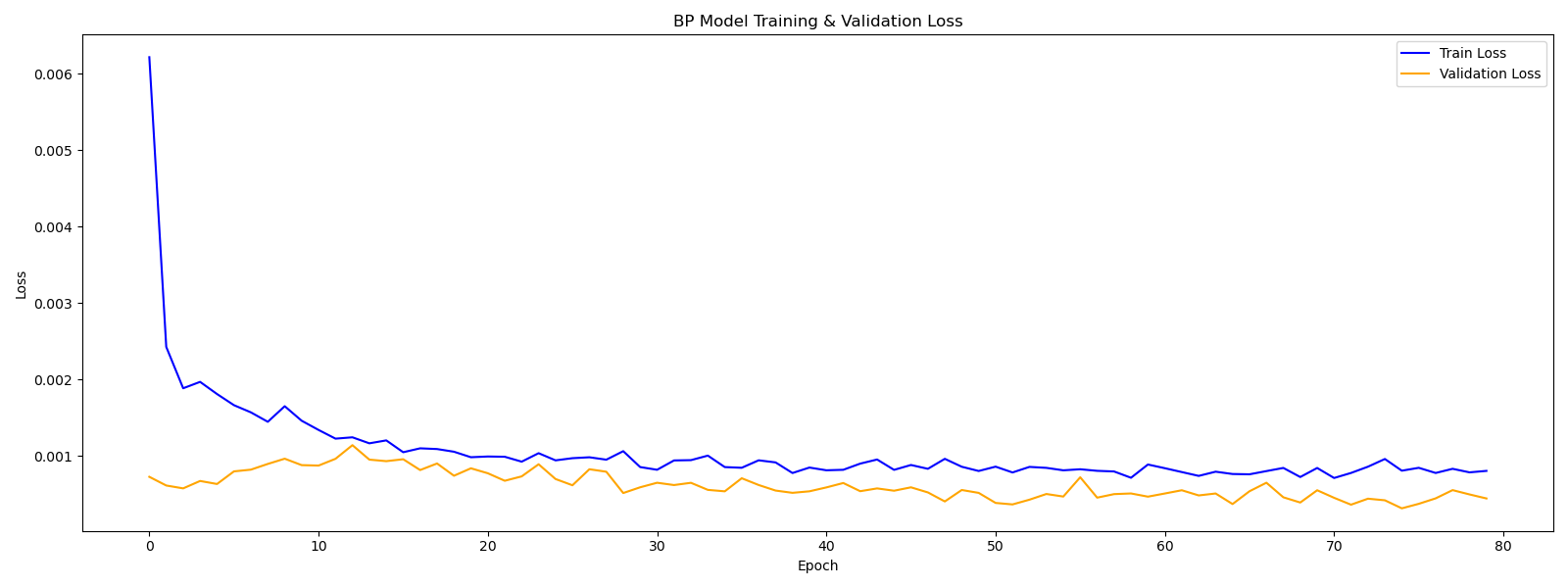

Supplement: S1 File — (ZIP) [file pone.0330324.s001.zip › Paper Model/SY/SANYUAN-600429.SS/GA-WOA-LSTM/figures/BP_training_loss.png]

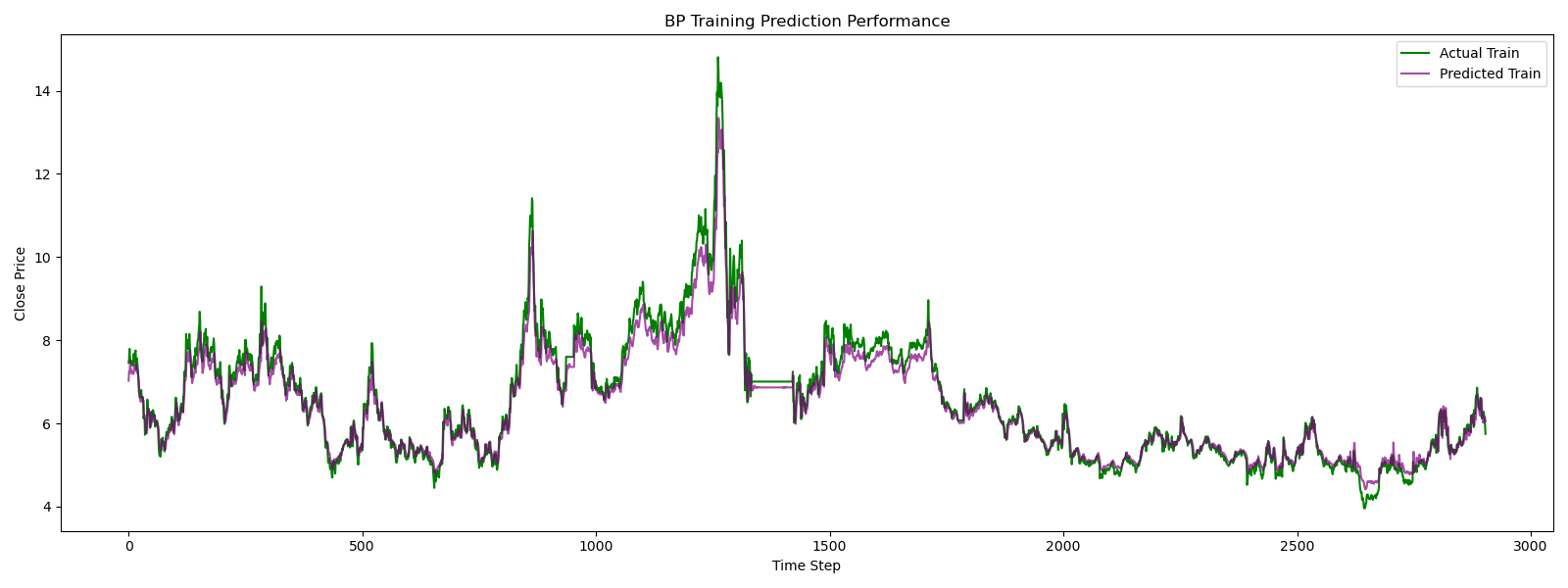

Supplement: S1 File — (ZIP) [file pone.0330324.s001.zip › Paper Model/SY/SANYUAN-600429.SS/GA-WOA-LSTM/figures/BP_train_fit_plot.png]

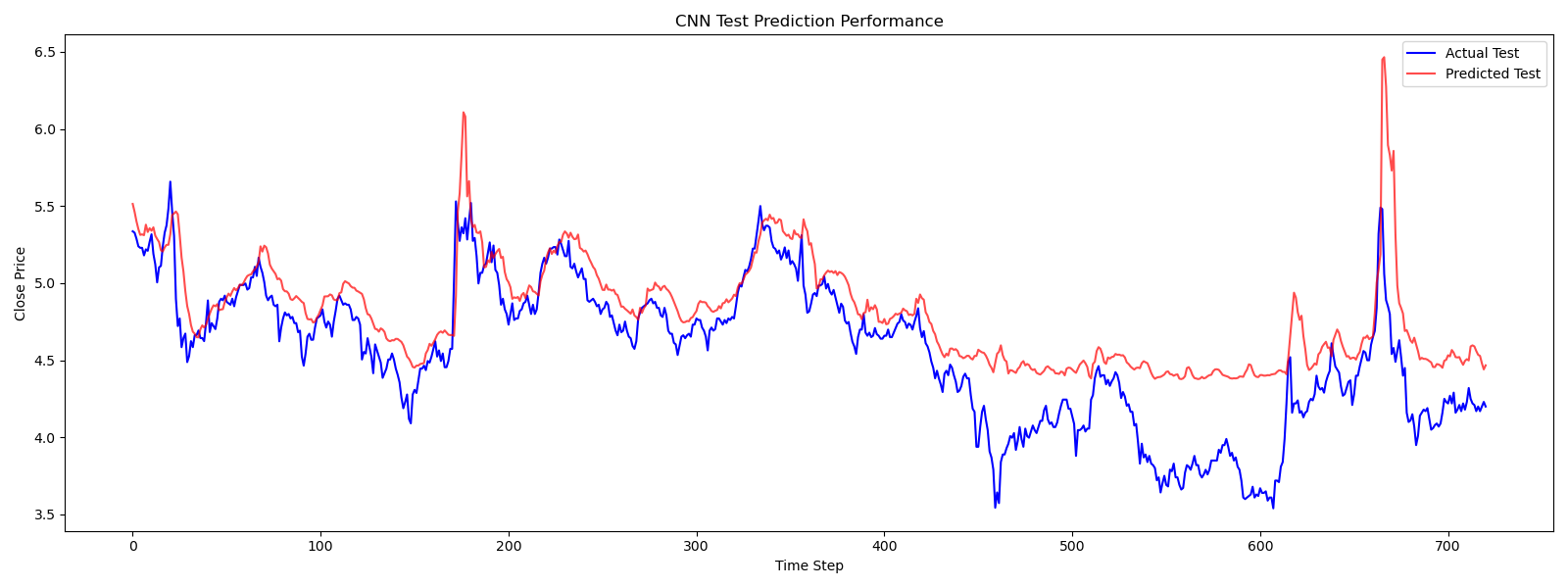

Supplement: S1 File — (ZIP) [file pone.0330324.s001.zip › Paper Model/SY/SANYUAN-600429.SS/GA-WOA-LSTM/figures/CNN_test_fit_plot.png]

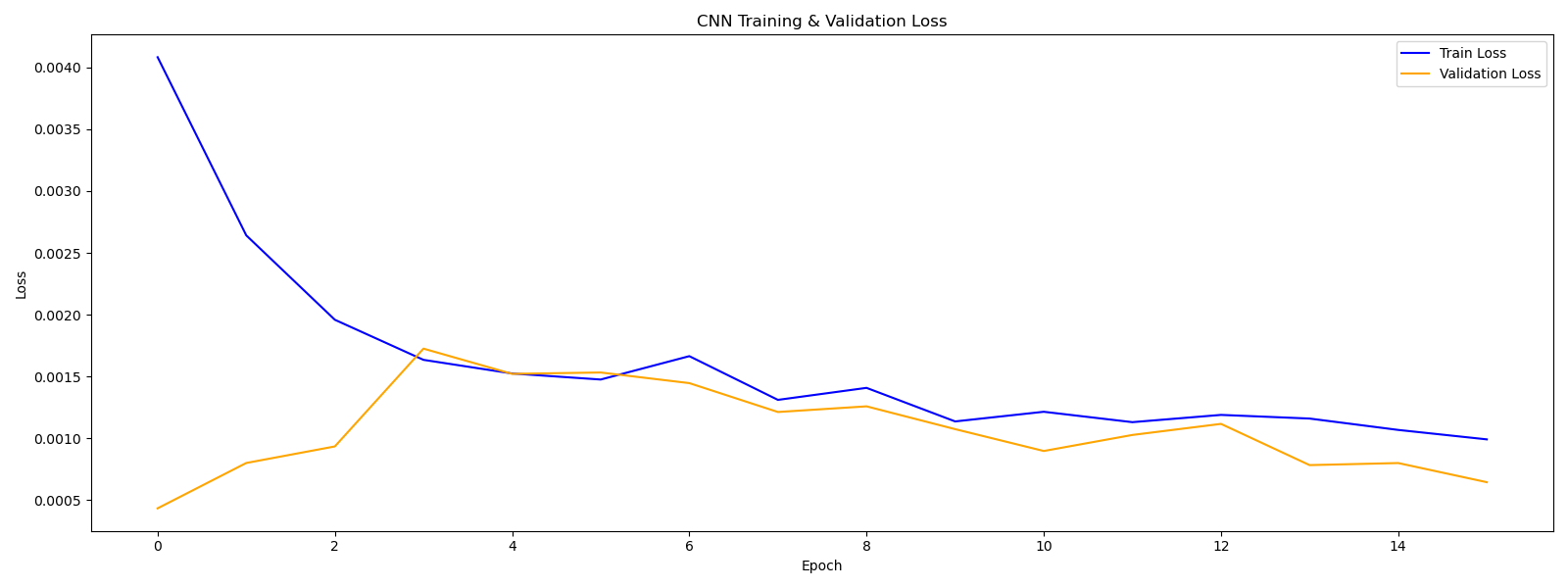

Supplement: S1 File — (ZIP) [file pone.0330324.s001.zip › Paper Model/SY/SANYUAN-600429.SS/GA-WOA-LSTM/figures/CNN_training_loss.png]

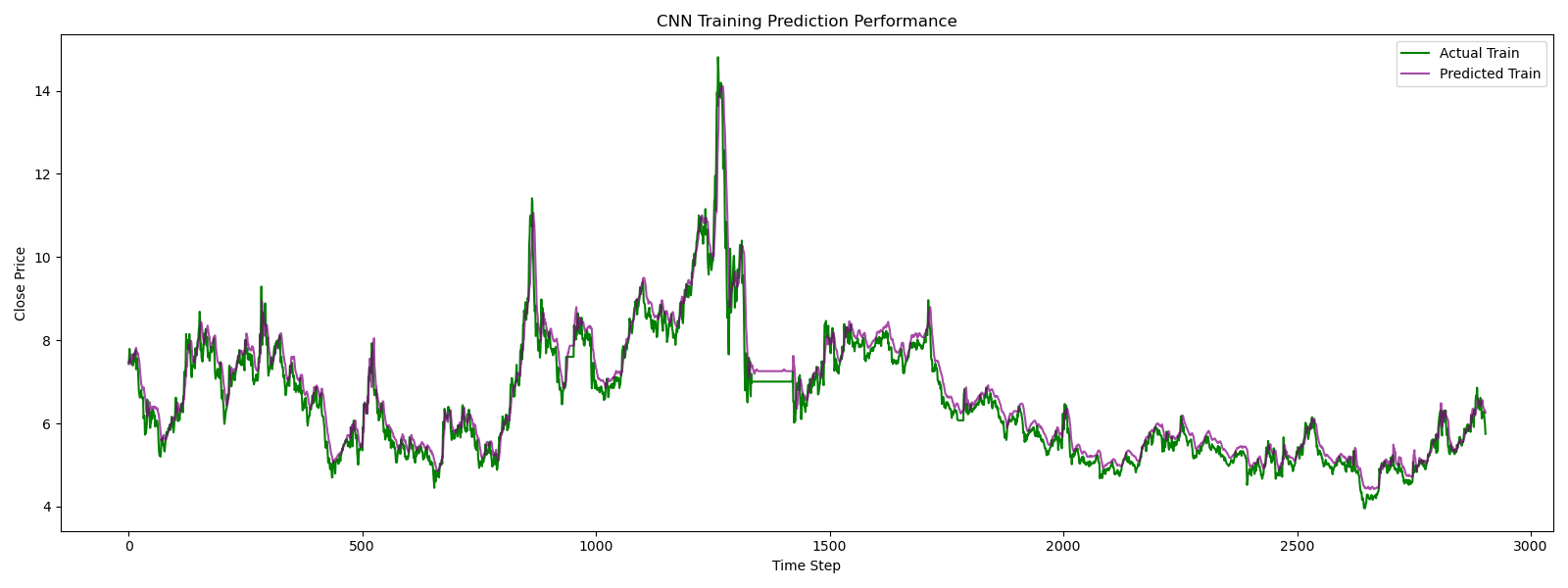

Supplement: S1 File — (ZIP) [file pone.0330324.s001.zip › Paper Model/SY/SANYUAN-600429.SS/GA-WOA-LSTM/figures/CNN_train_fit_plot.png]

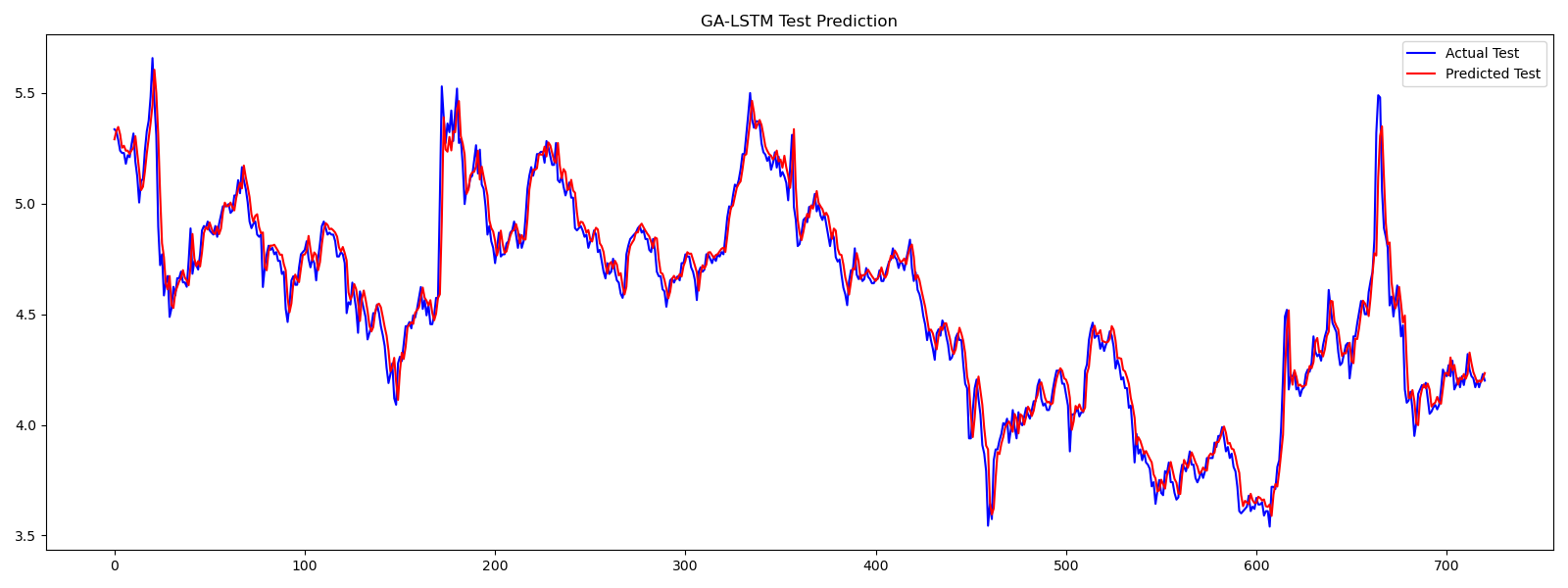

Supplement: S1 File — (ZIP) [file pone.0330324.s001.zip › Paper Model/SY/SANYUAN-600429.SS/GA-WOA-LSTM/figures/GA_LSTM_test_fit_plot.png]

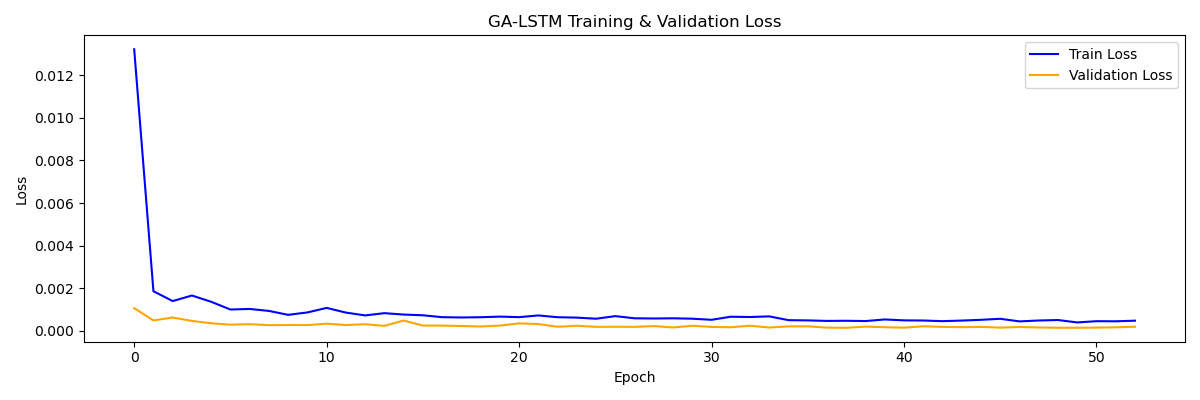

Supplement: S1 File — (ZIP) [file pone.0330324.s001.zip › Paper Model/SY/SANYUAN-600429.SS/GA-WOA-LSTM/figures/GA_LSTM_training_loss.png]

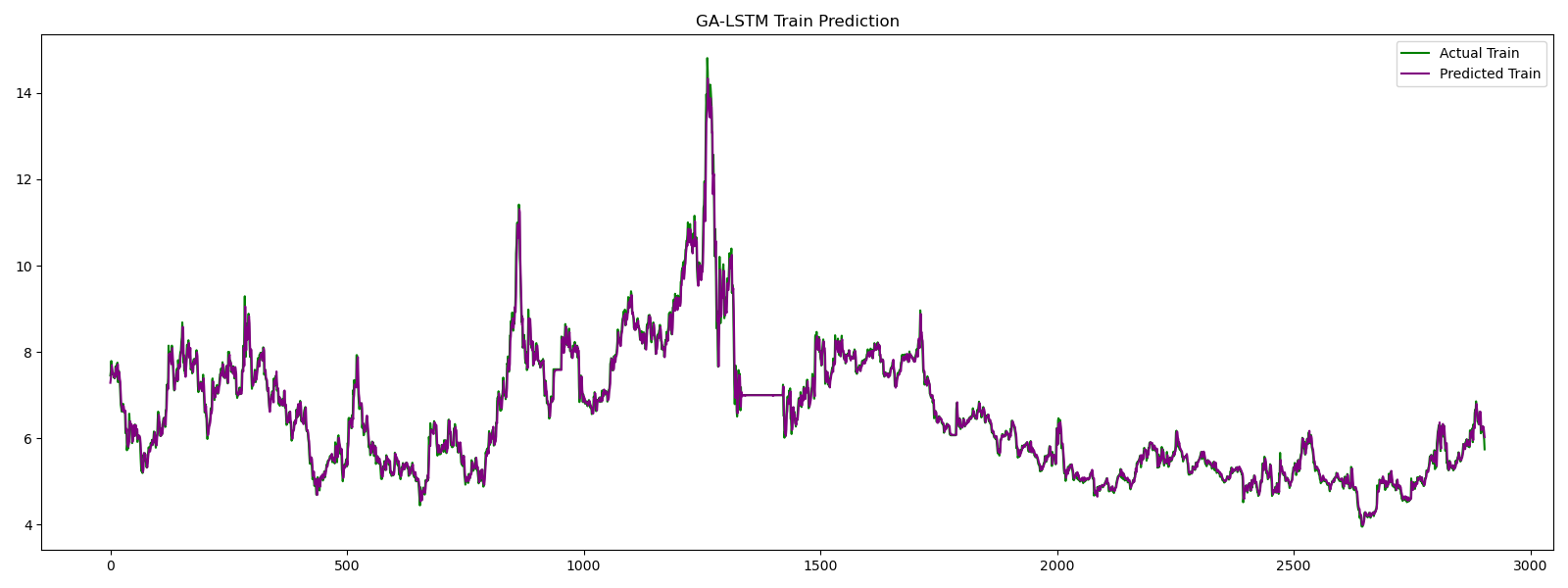

Supplement: S1 File — (ZIP) [file pone.0330324.s001.zip › Paper Model/SY/SANYUAN-600429.SS/GA-WOA-LSTM/figures/GA_LSTM_train_fit_plot.png]

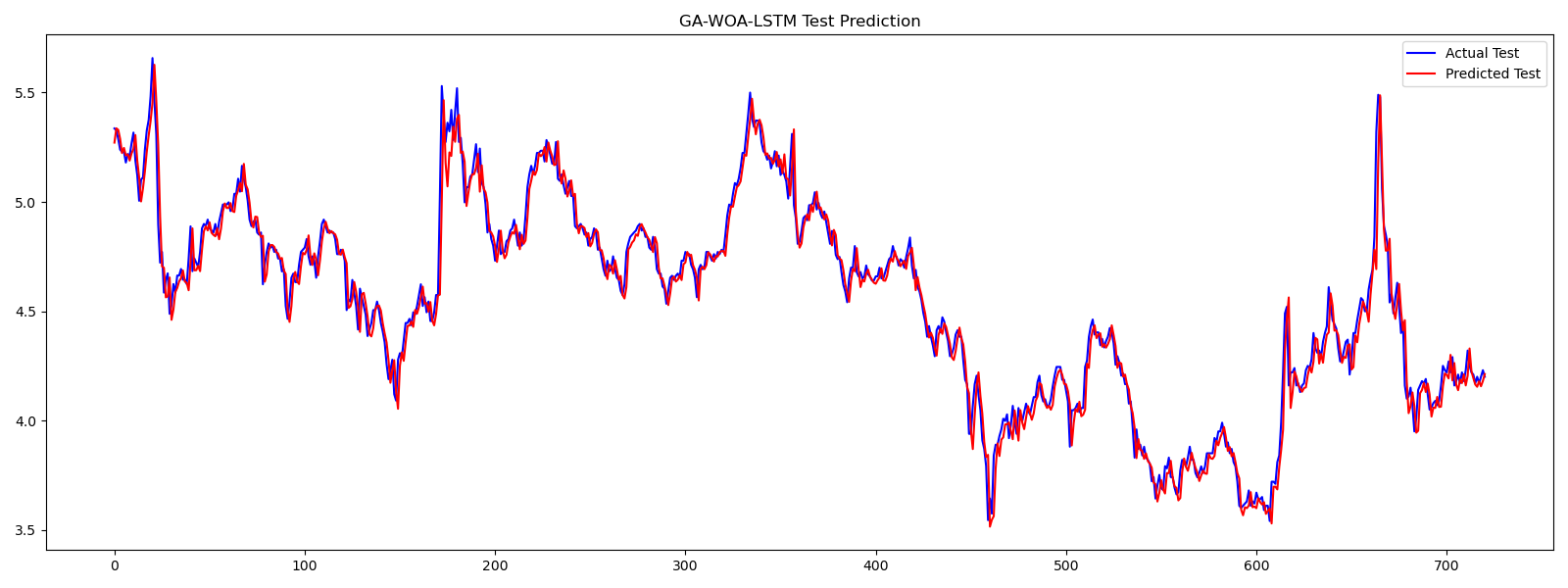

Supplement: S1 File — (ZIP) [file pone.0330324.s001.zip › Paper Model/SY/SANYUAN-600429.SS/GA-WOA-LSTM/figures/GA_WOA_LSTM_test_fit_plot.png]

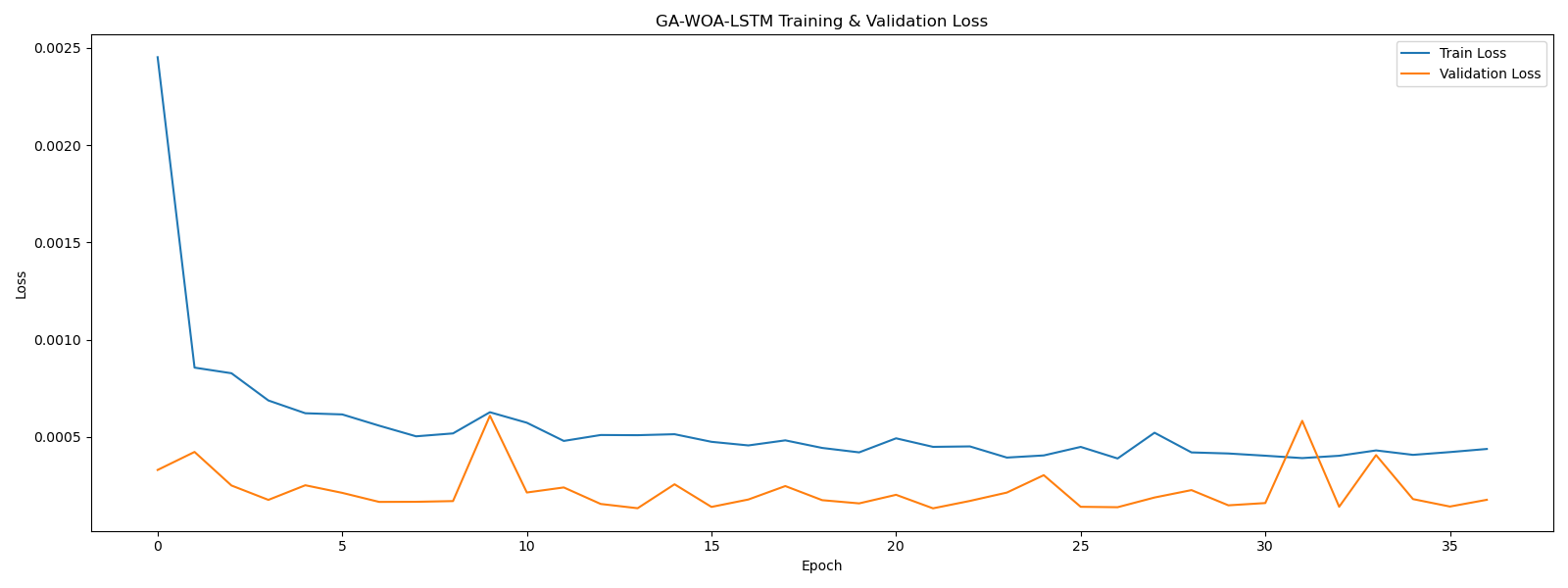

Supplement: S1 File — (ZIP) [file pone.0330324.s001.zip › Paper Model/SY/SANYUAN-600429.SS/GA-WOA-LSTM/figures/GA_WOA_LSTM_training_loss.png]

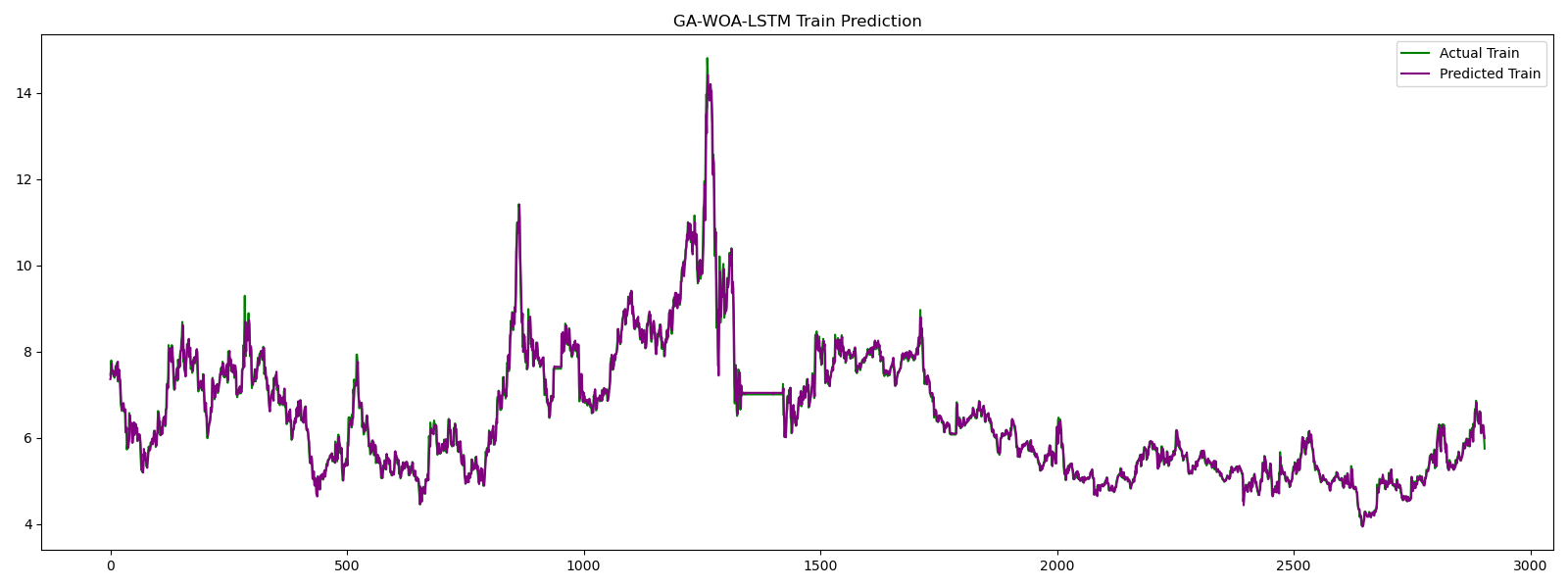

Supplement: S1 File — (ZIP) [file pone.0330324.s001.zip › Paper Model/SY/SANYUAN-600429.SS/GA-WOA-LSTM/figures/GA_WOA_LSTM_train_fit_plot.png]

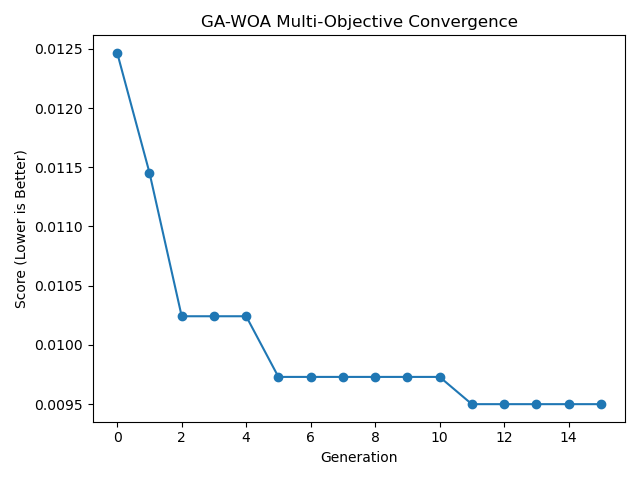

Supplement: S1 File — (ZIP) [file pone.0330324.s001.zip › Paper Model/SY/SANYUAN-600429.SS/GA-WOA-LSTM/figures/GA_WOA_multiobj_convergence_curve.png]

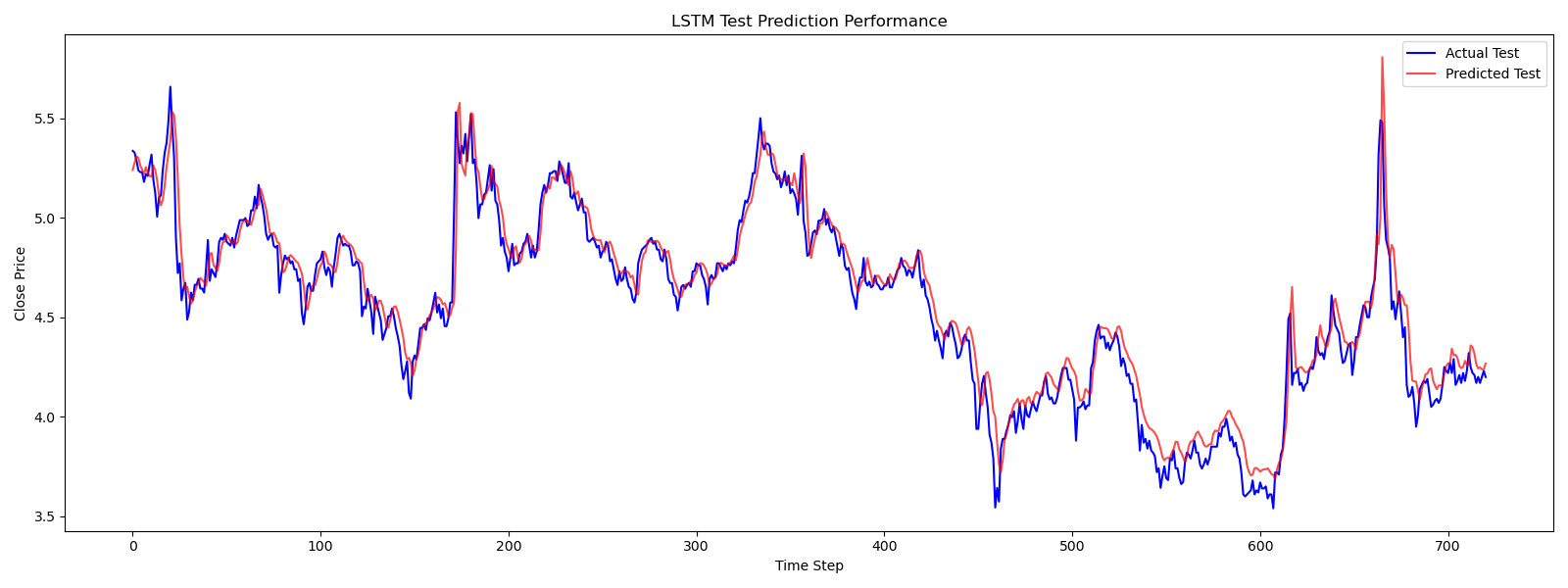

Supplement: S1 File — (ZIP) [file pone.0330324.s001.zip › Paper Model/SY/SANYUAN-600429.SS/GA-WOA-LSTM/figures/LSTM_test_fit_plot.png]

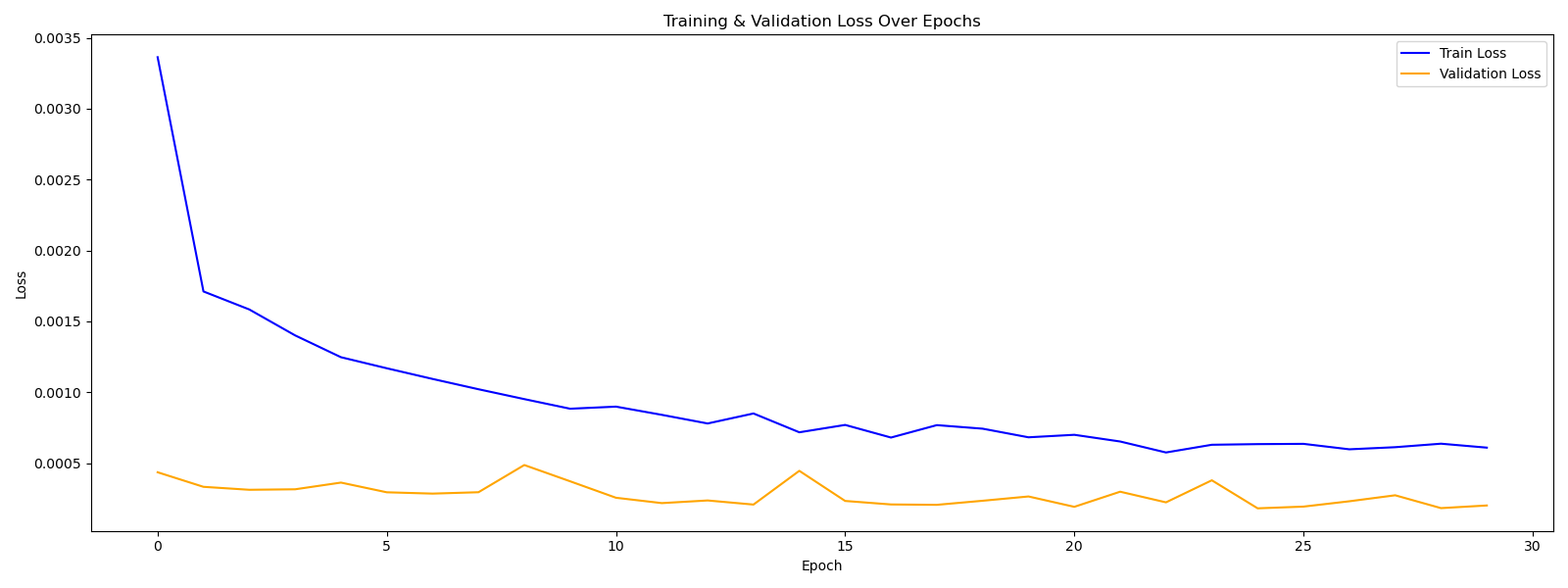

Supplement: S1 File — (ZIP) [file pone.0330324.s001.zip › Paper Model/SY/SANYUAN-600429.SS/GA-WOA-LSTM/figures/LSTM_training_loss.png]

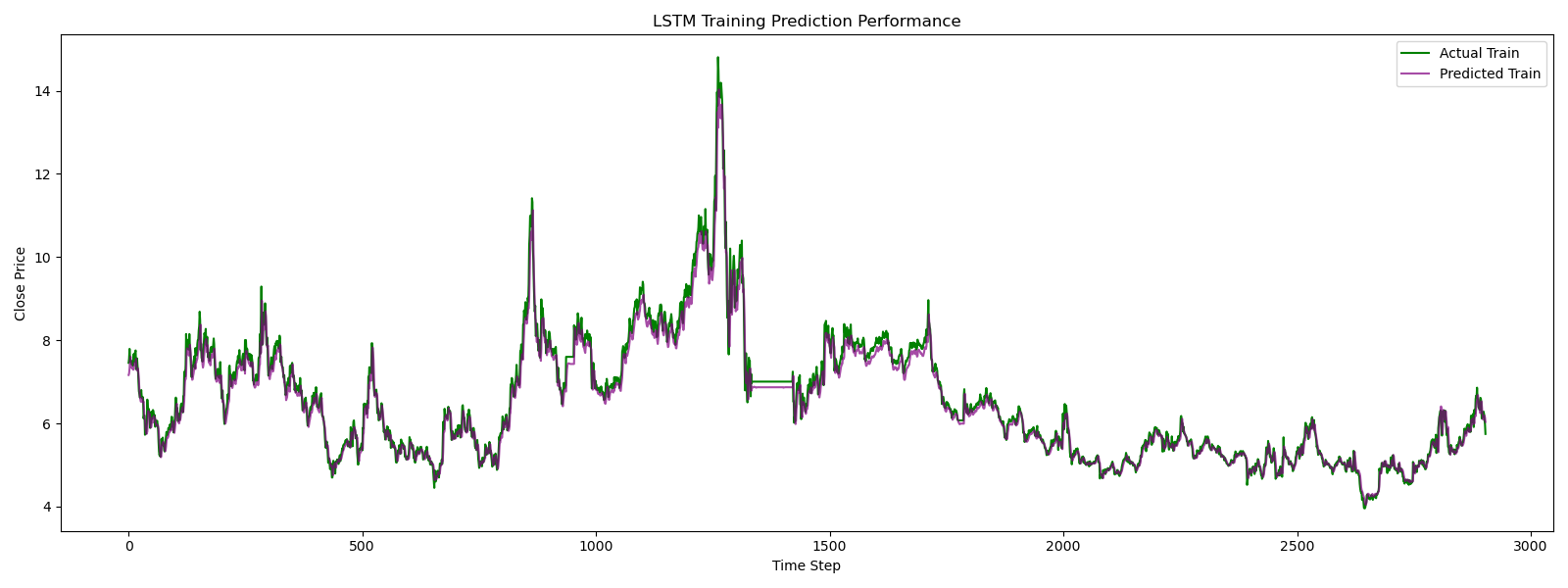

Supplement: S1 File — (ZIP) [file pone.0330324.s001.zip › Paper Model/SY/SANYUAN-600429.SS/GA-WOA-LSTM/figures/LSTM_train_fit_plot.png]

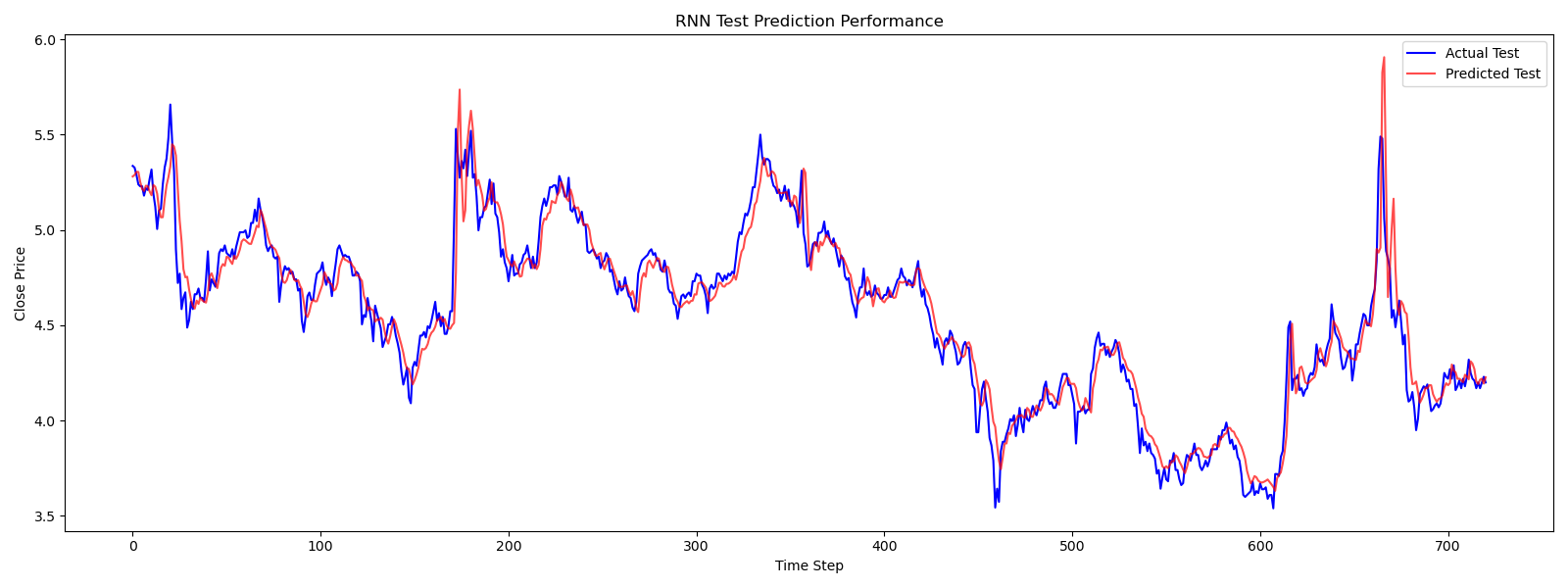

Supplement: S1 File — (ZIP) [file pone.0330324.s001.zip › Paper Model/SY/SANYUAN-600429.SS/GA-WOA-LSTM/figures/RNN_test_fit_plot.png]

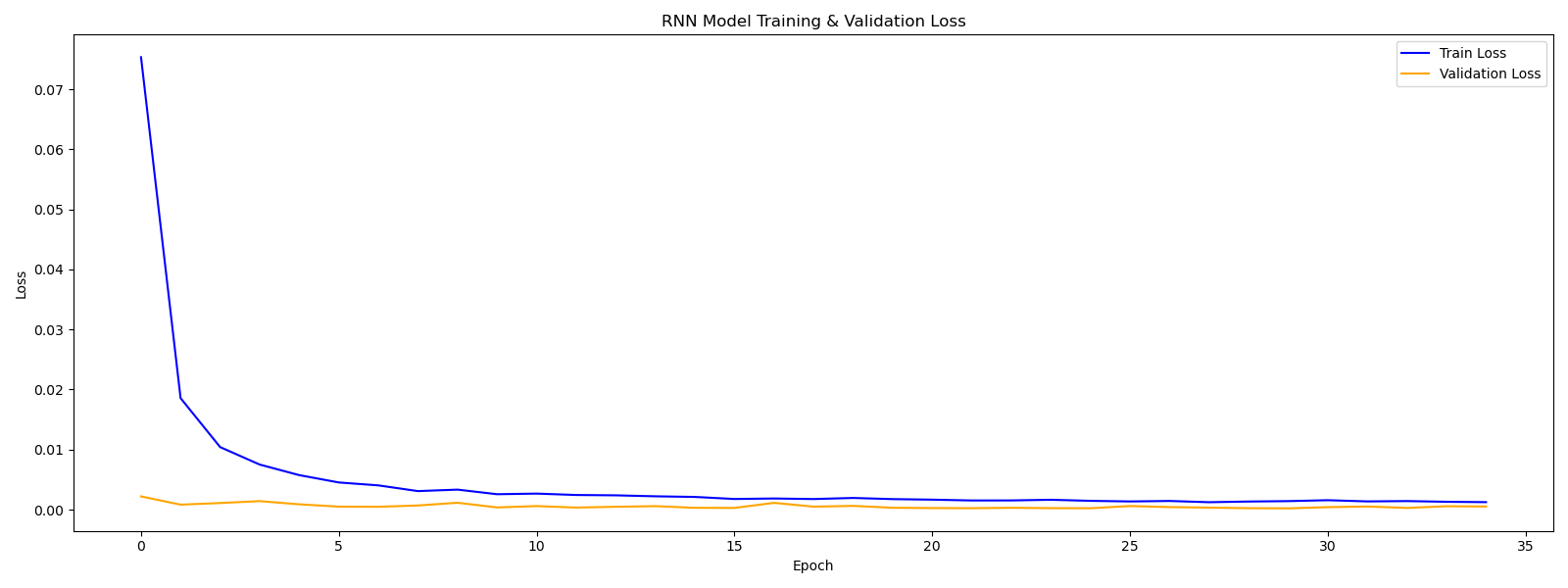

Supplement: S1 File — (ZIP) [file pone.0330324.s001.zip › Paper Model/SY/SANYUAN-600429.SS/GA-WOA-LSTM/figures/RNN_training_loss.png]

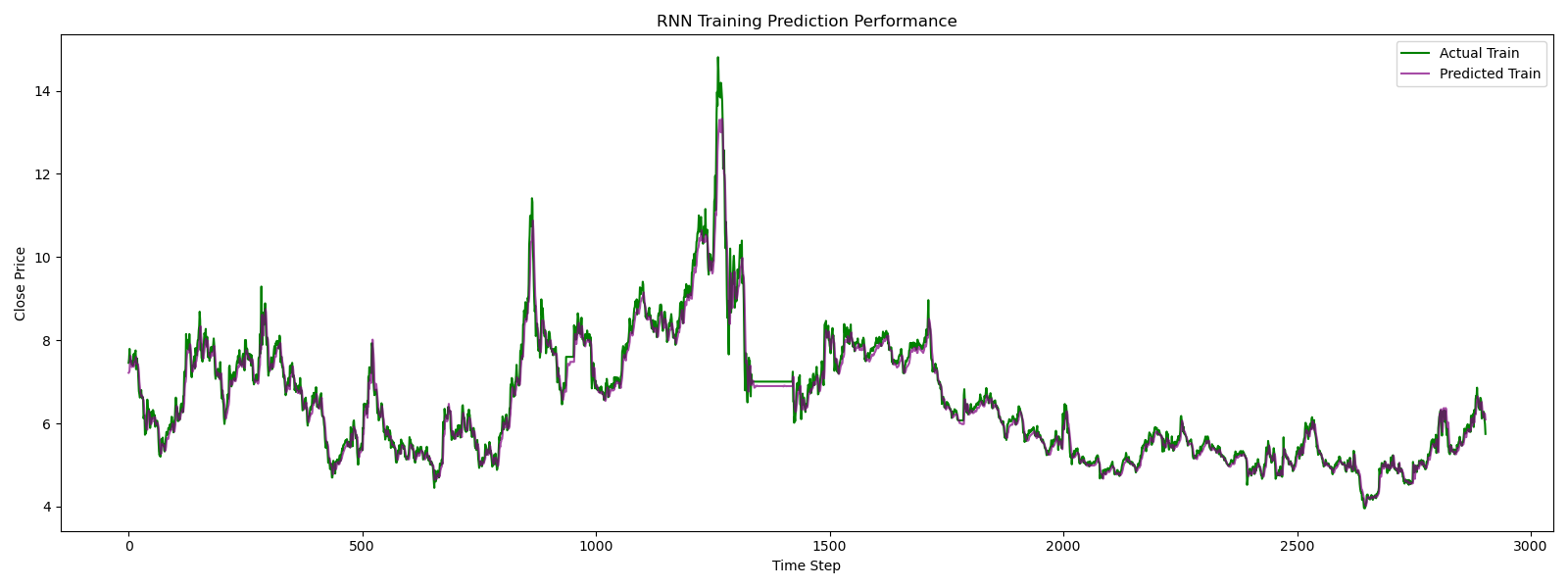

Supplement: S1 File — (ZIP) [file pone.0330324.s001.zip › Paper Model/SY/SANYUAN-600429.SS/GA-WOA-LSTM/figures/RNN_train_fit_plot.png]

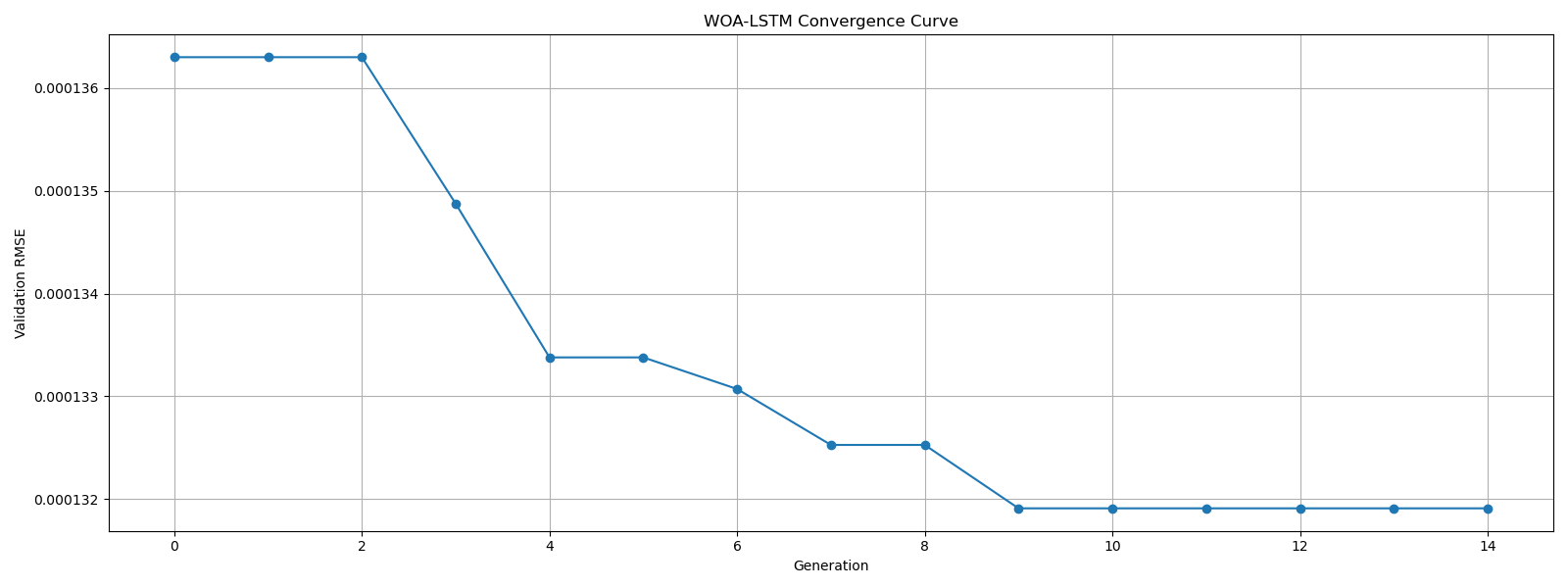

Supplement: S1 File — (ZIP) [file pone.0330324.s001.zip › Paper Model/SY/SANYUAN-600429.SS/GA-WOA-LSTM/figures/WOA_LSTM_convergence_curve.png]

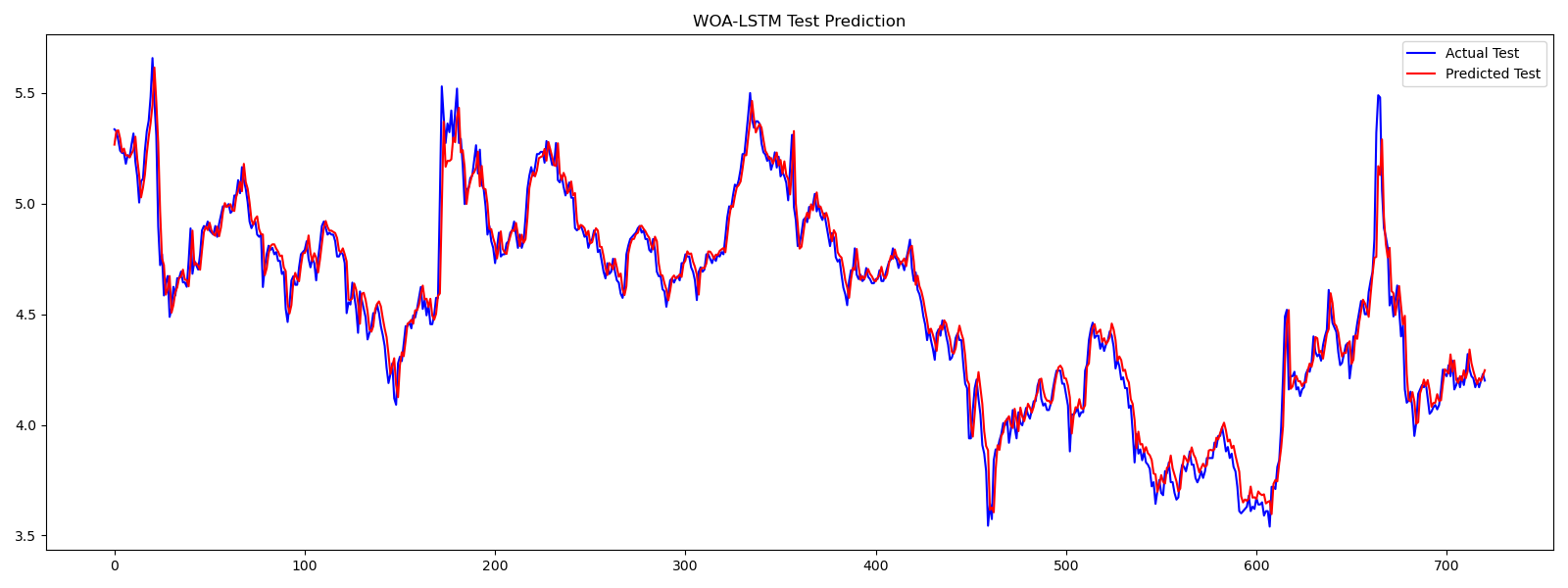

Supplement: S1 File — (ZIP) [file pone.0330324.s001.zip › Paper Model/SY/SANYUAN-600429.SS/GA-WOA-LSTM/figures/WOA_LSTM_test_fit_plot.png]

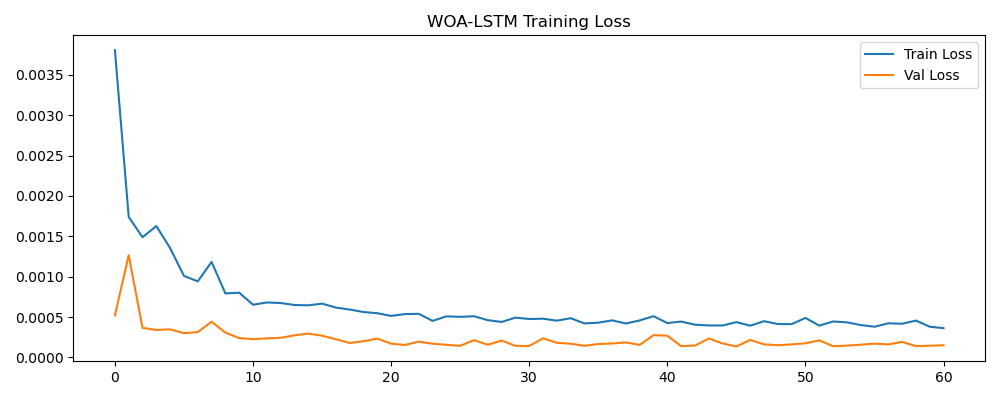

Supplement: S1 File — (ZIP) [file pone.0330324.s001.zip › Paper Model/SY/SANYUAN-600429.SS/GA-WOA-LSTM/figures/WOA_LSTM_training_loss.png]

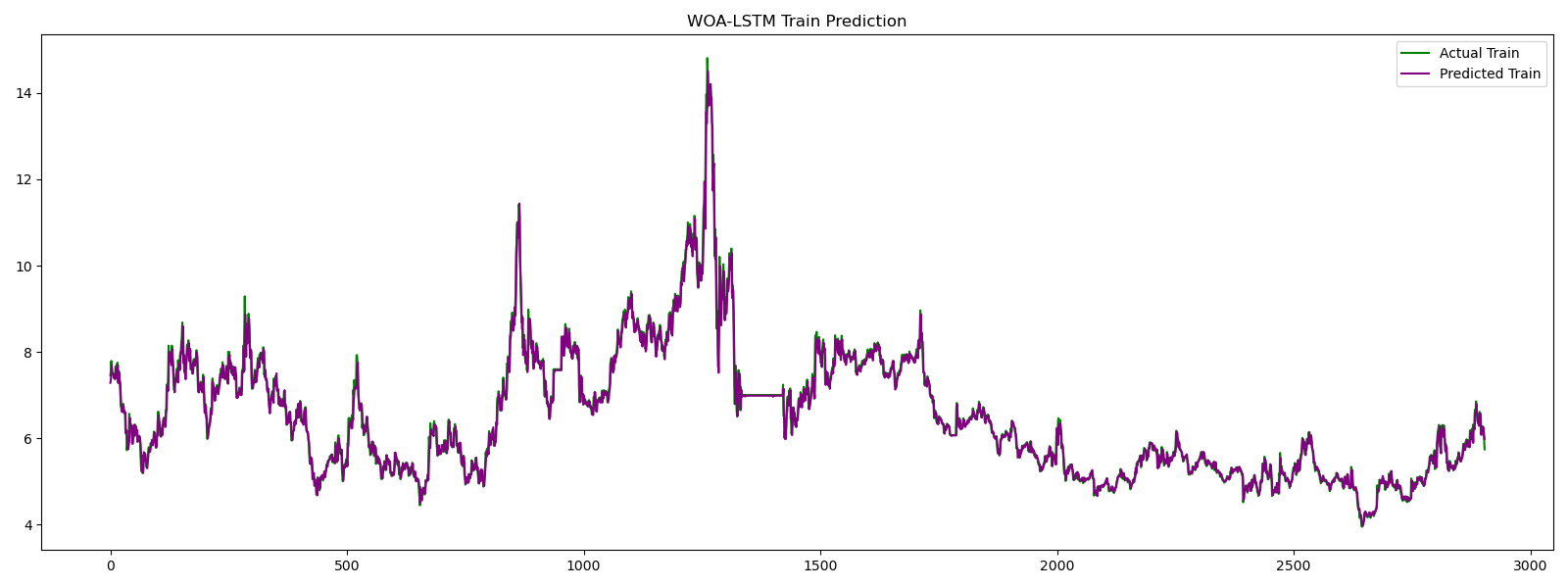

Supplement: S1 File — (ZIP) [file pone.0330324.s001.zip › Paper Model/SY/SANYUAN-600429.SS/GA-WOA-LSTM/figures/WOA_LSTM_train_fit_plot.png]

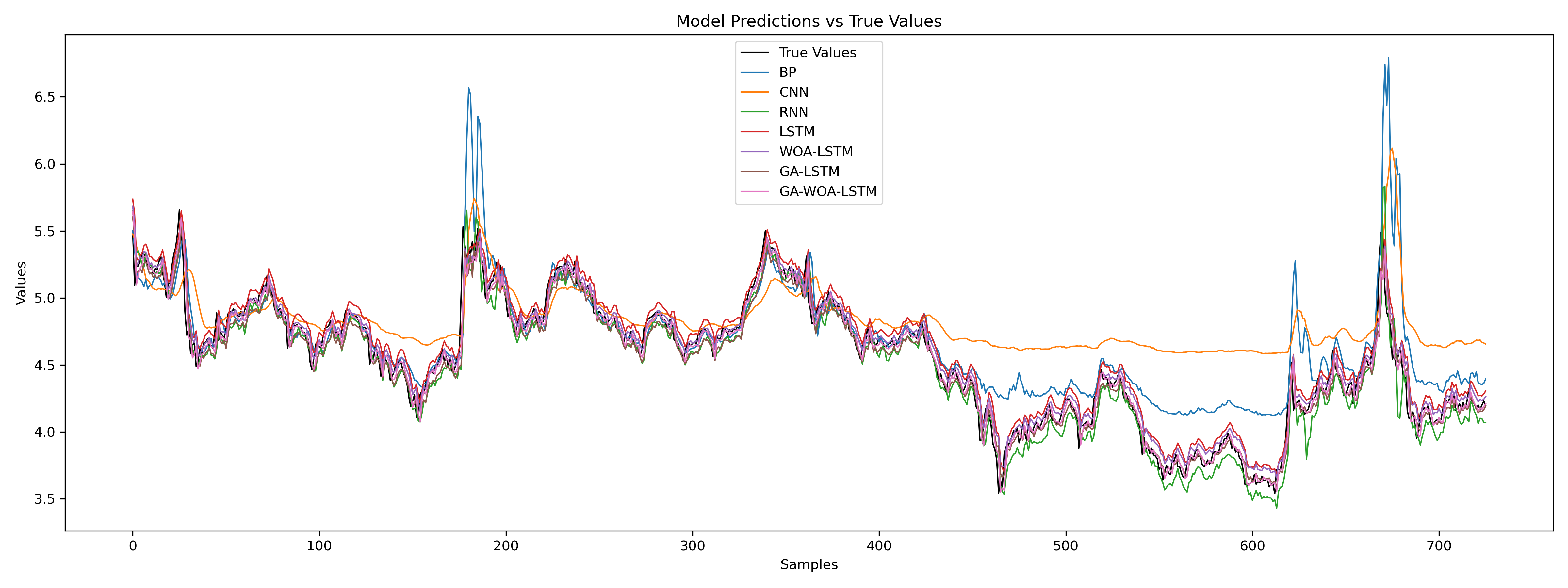

Supplement: S1 File — (ZIP) [file pone.0330324.s001.zip › Paper Model/SY/SANYUAN-600429.SS/model_comparison_style_match.png]

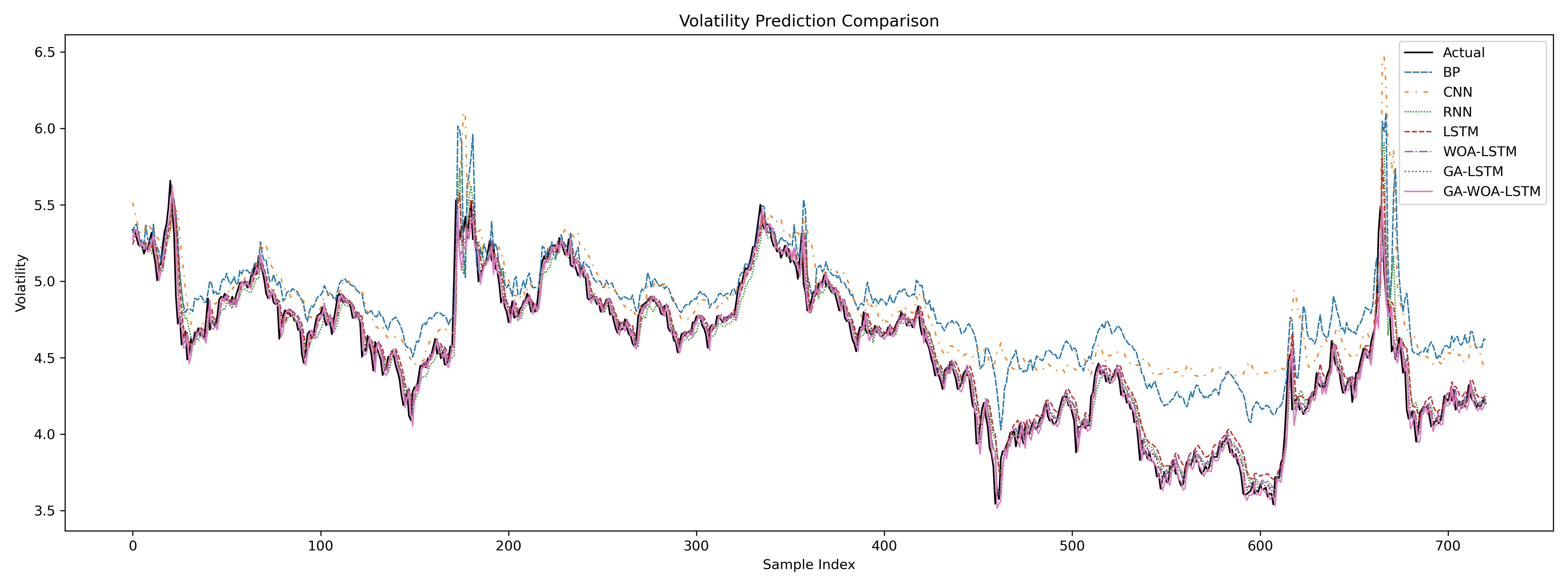

Supplement: S1 File — (ZIP) [file pone.0330324.s001.zip › Paper Model/SY/SANYUAN-600429.SS/test_model_comparison_plot.png]

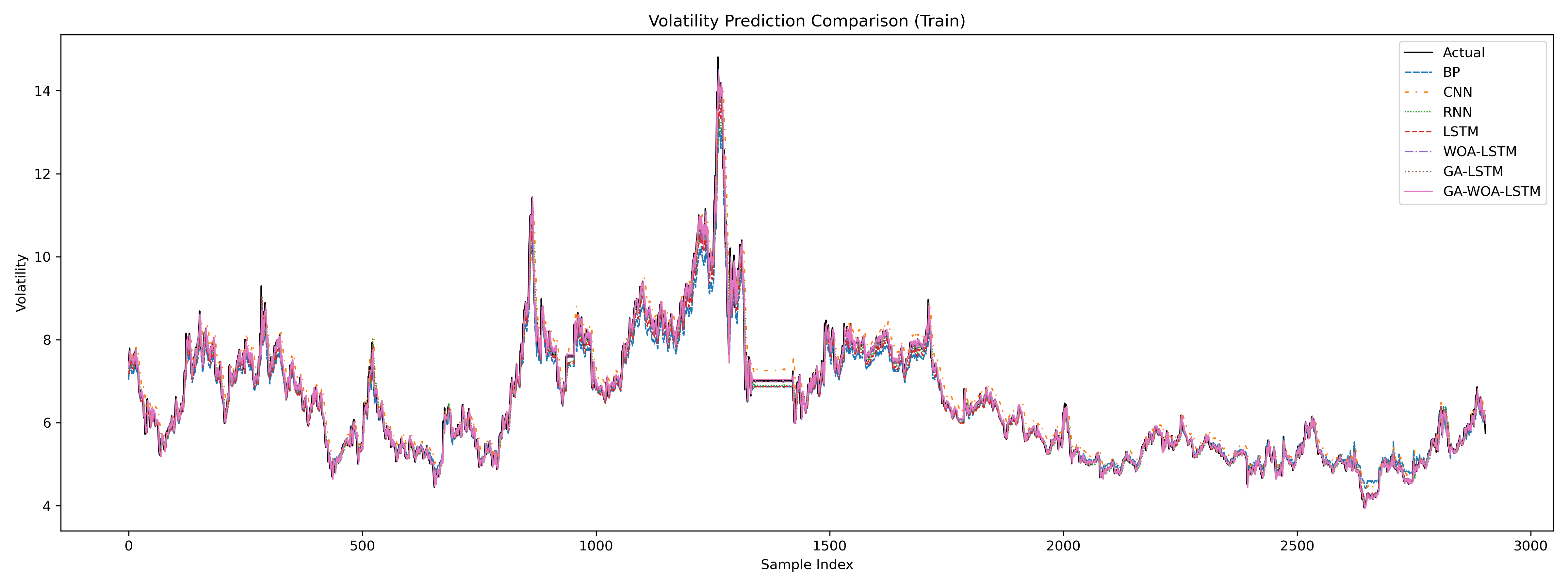

Supplement: S1 File — (ZIP) [file pone.0330324.s001.zip › Paper Model/SY/SANYUAN-600429.SS/train_model_comparison_plot.png]

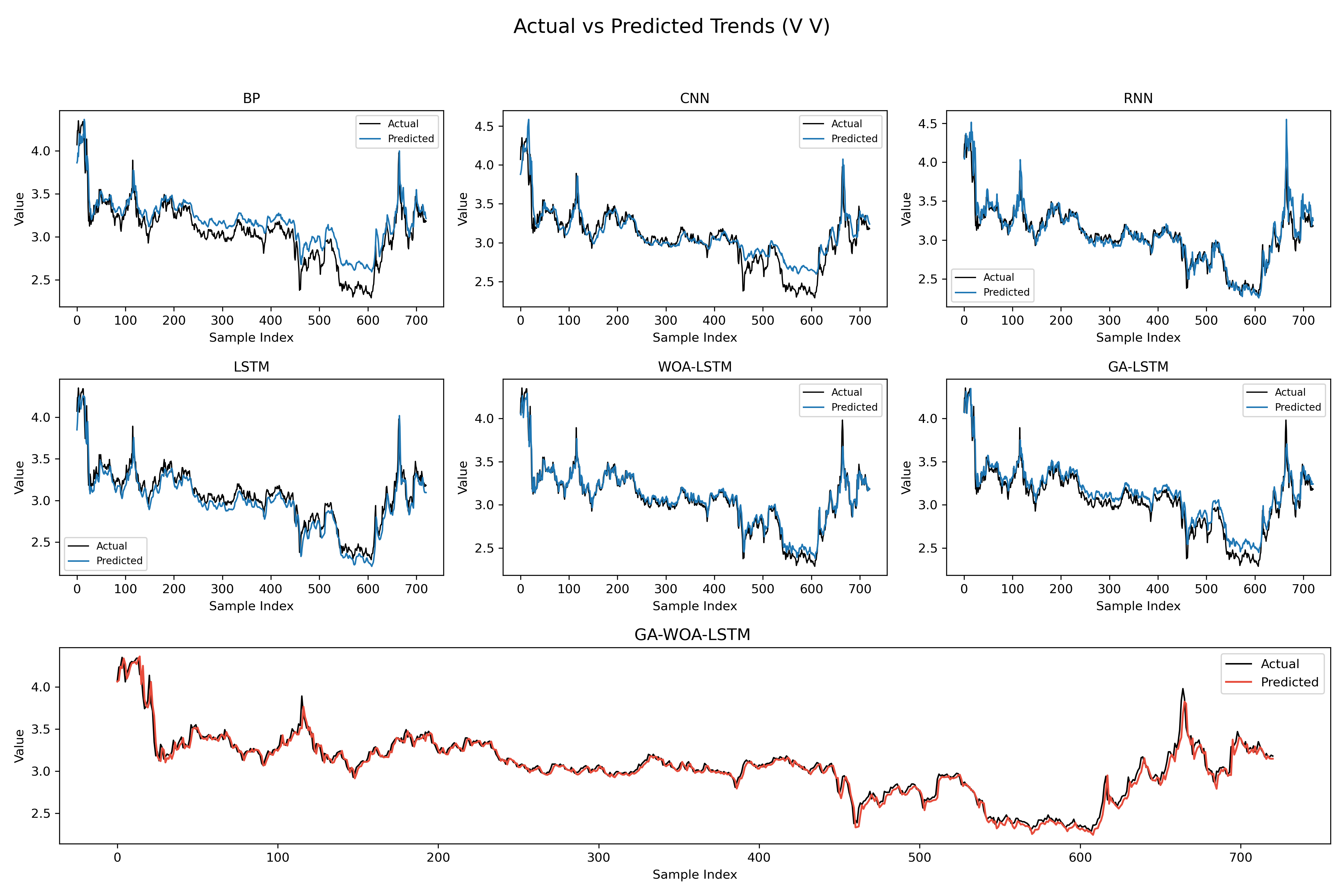

Supplement: S1 File — (ZIP) [file pone.0330324.s001.zip › Paper Model/V V/V V-600300/figures/actual_vs_predicted_7models_highlighted.png]

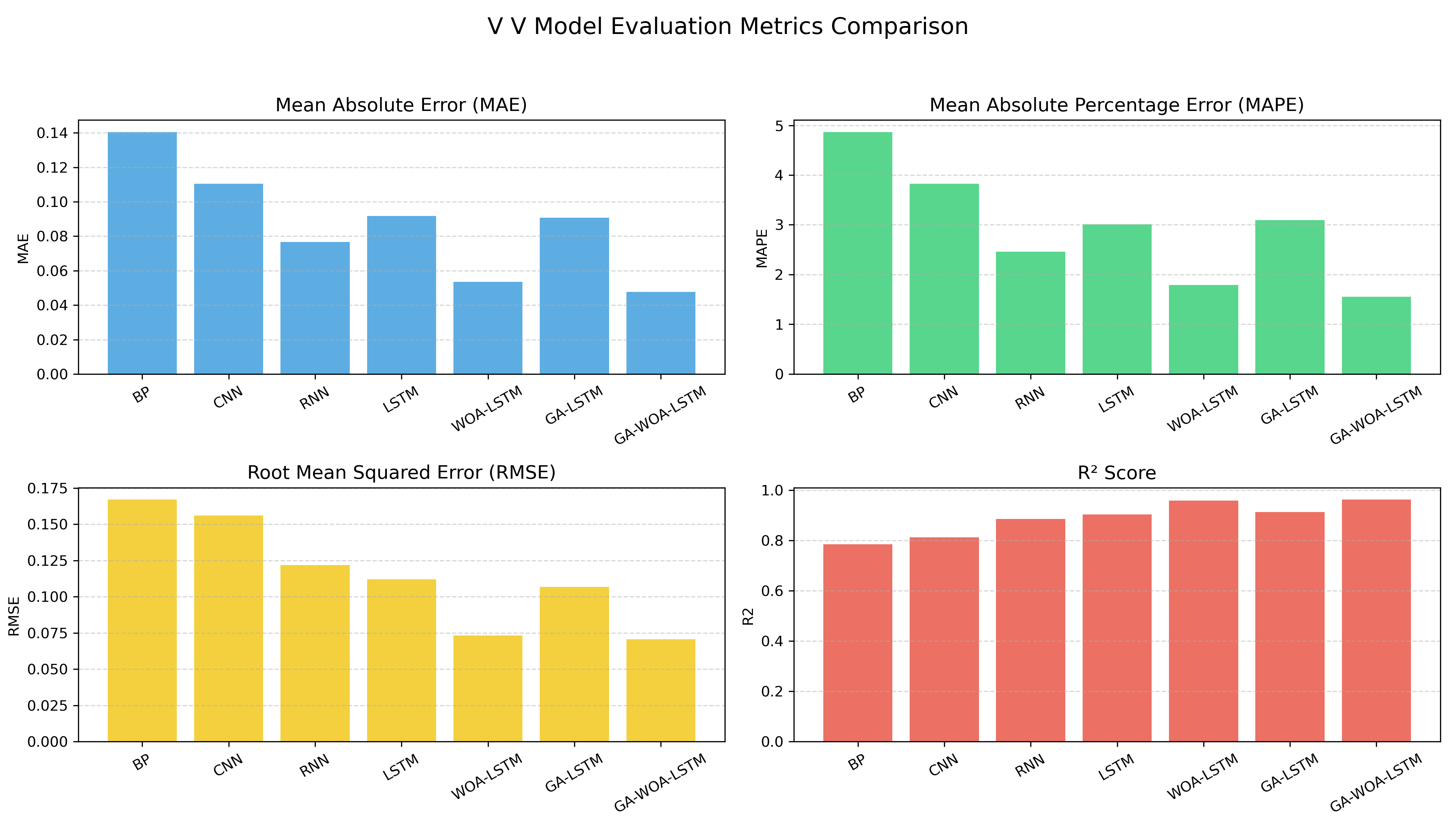

Supplement: S1 File — (ZIP) [file pone.0330324.s001.zip › Paper Model/V V/V V-600300/figures/test_model_metrics_comparison_grid.png]

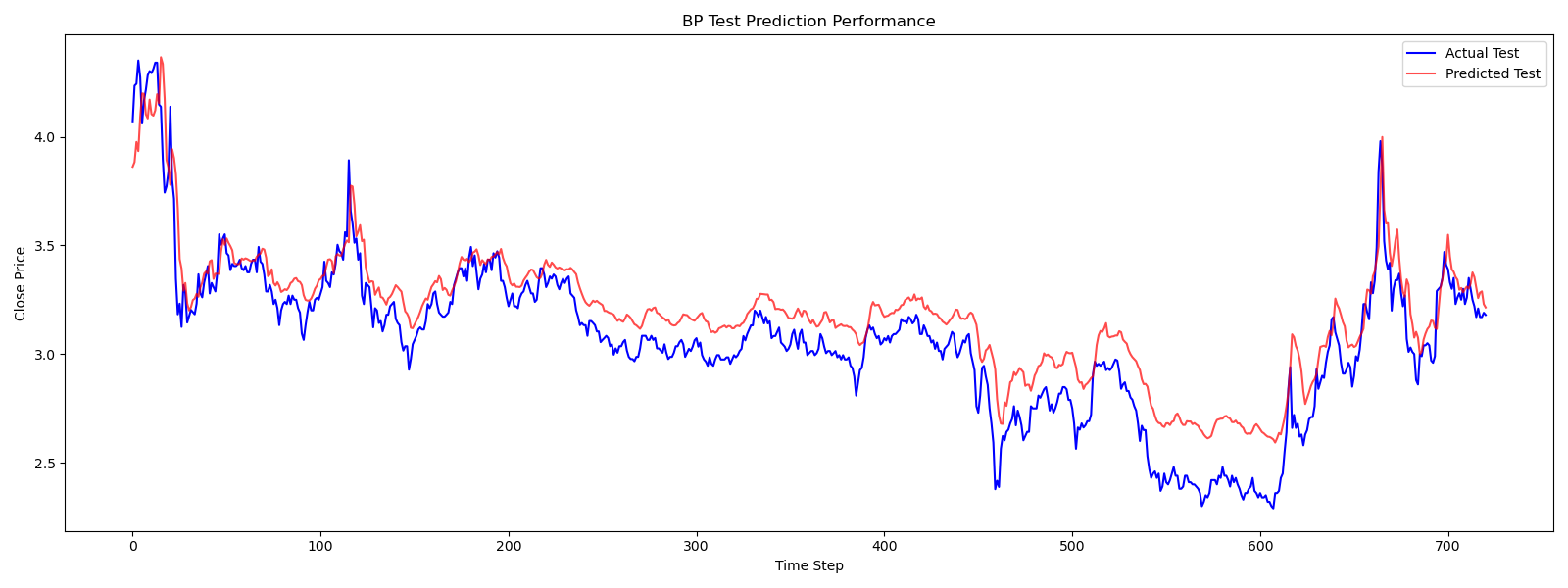

Supplement: S1 File — (ZIP) [file pone.0330324.s001.zip › Paper Model/V V/V V-600300/GA-WOA-LSTM/figures/BP_test_fit_plot.png]

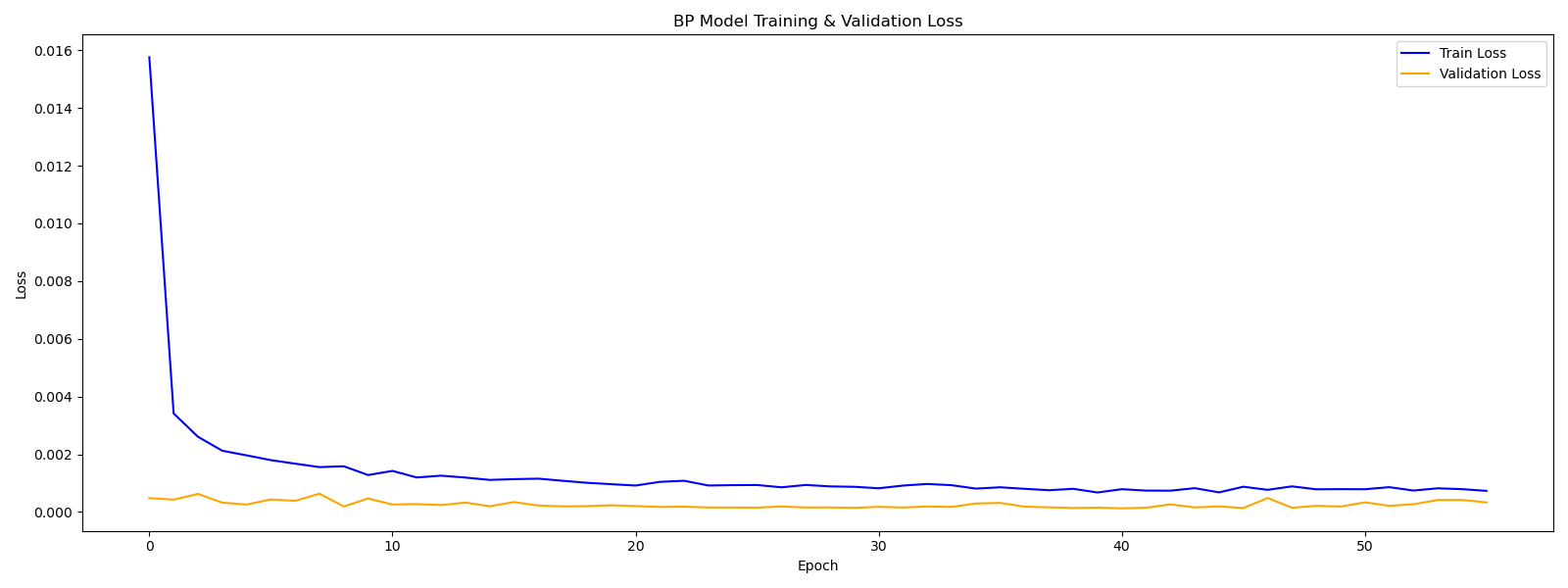

Supplement: S1 File — (ZIP) [file pone.0330324.s001.zip › Paper Model/V V/V V-600300/GA-WOA-LSTM/figures/BP_training_loss.png]

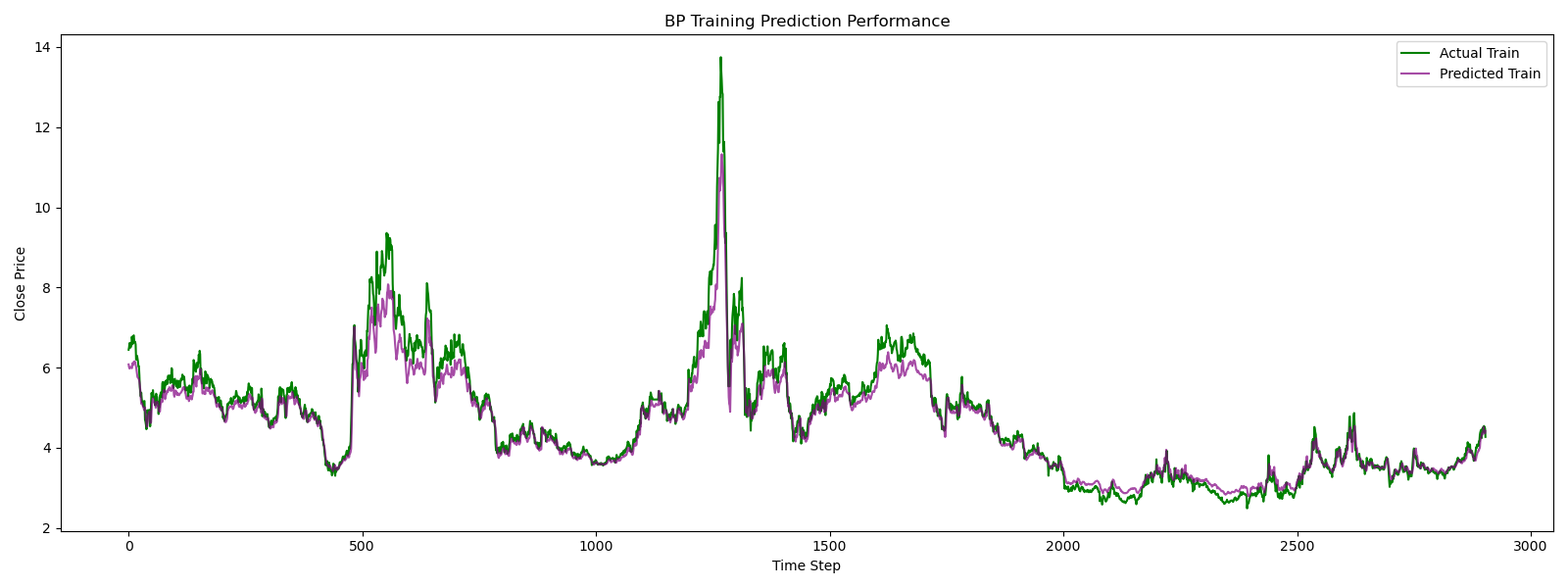

Supplement: S1 File — (ZIP) [file pone.0330324.s001.zip › Paper Model/V V/V V-600300/GA-WOA-LSTM/figures/BP_train_fit_plot.png]

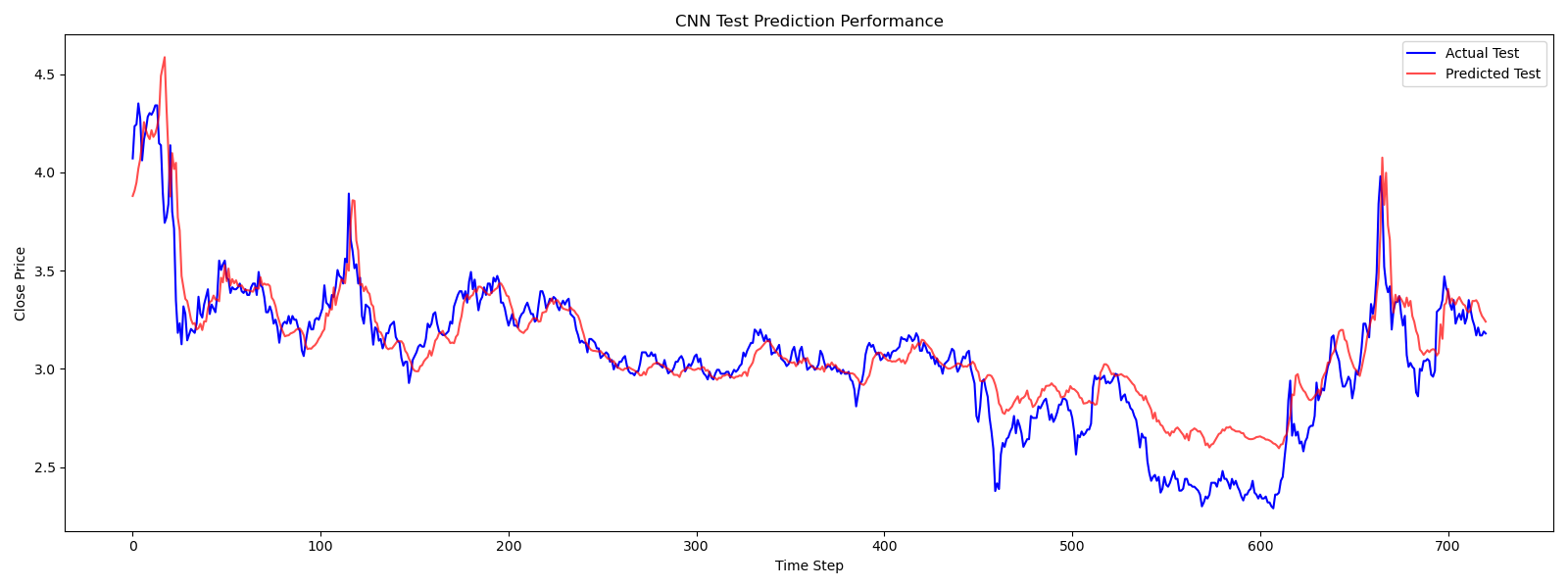

Supplement: S1 File — (ZIP) [file pone.0330324.s001.zip › Paper Model/V V/V V-600300/GA-WOA-LSTM/figures/CNN_test_fit_plot.png]

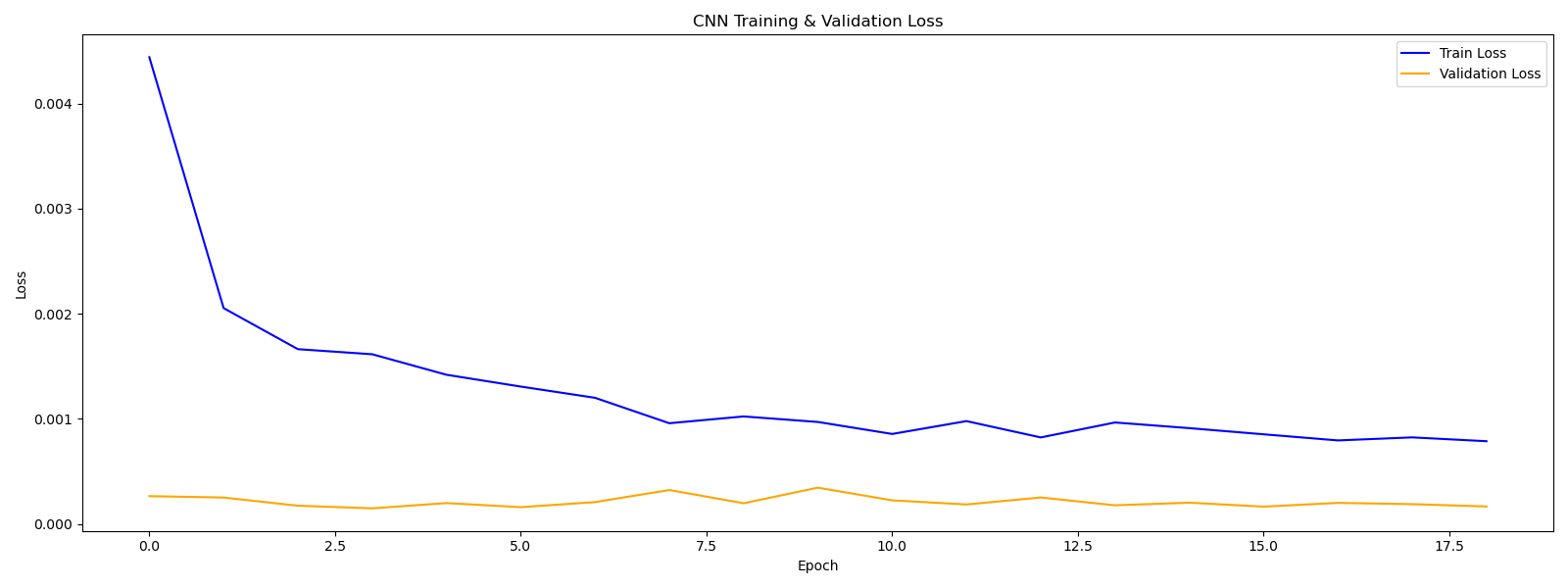

Supplement: S1 File — (ZIP) [file pone.0330324.s001.zip › Paper Model/V V/V V-600300/GA-WOA-LSTM/figures/CNN_training_loss.png]

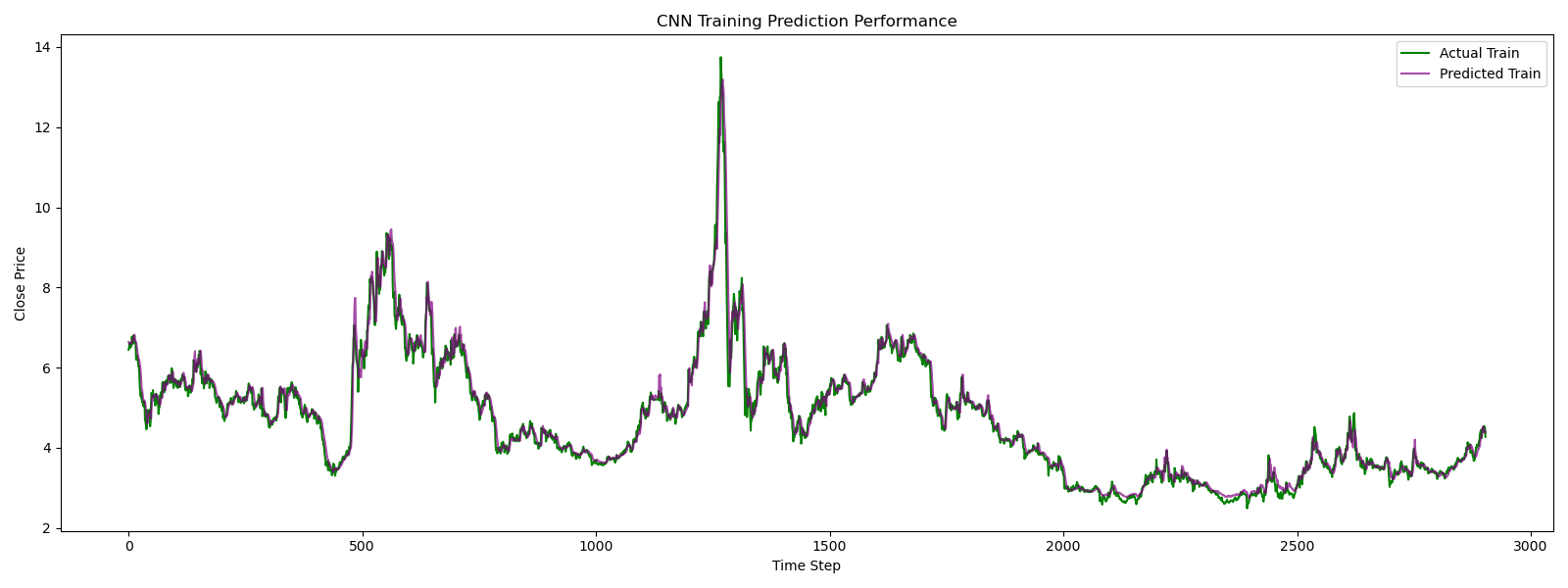

Supplement: S1 File — (ZIP) [file pone.0330324.s001.zip › Paper Model/V V/V V-600300/GA-WOA-LSTM/figures/CNN_train_fit_plot.png]

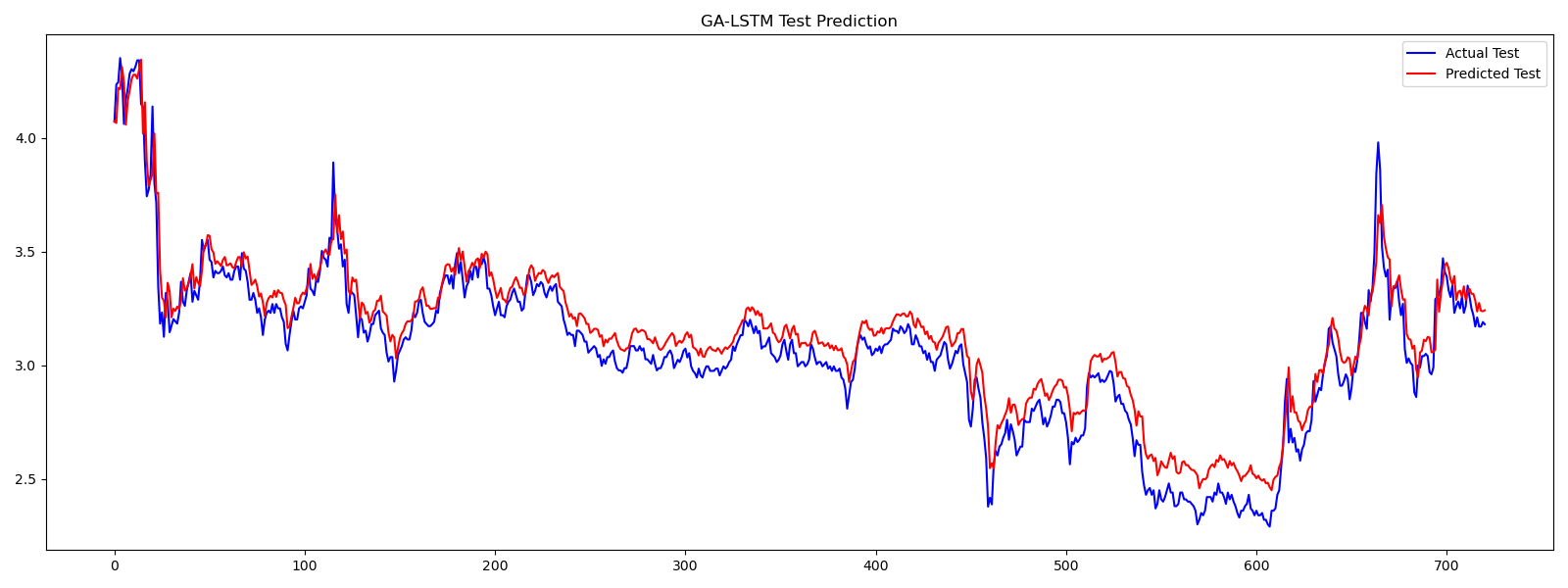

Supplement: S1 File — (ZIP) [file pone.0330324.s001.zip › Paper Model/V V/V V-600300/GA-WOA-LSTM/figures/GA_LSTM_test_fit_plot.png]

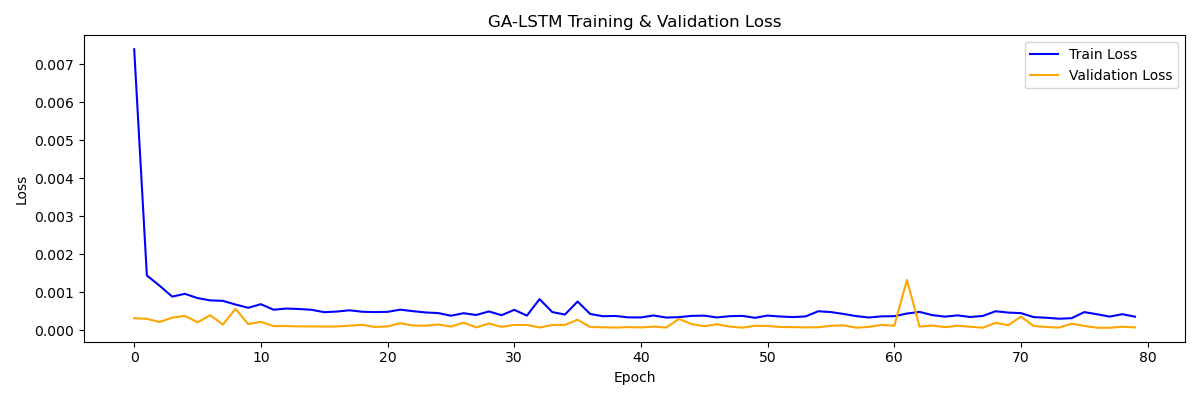

Supplement: S1 File — (ZIP) [file pone.0330324.s001.zip › Paper Model/V V/V V-600300/GA-WOA-LSTM/figures/GA_LSTM_training_loss.png]

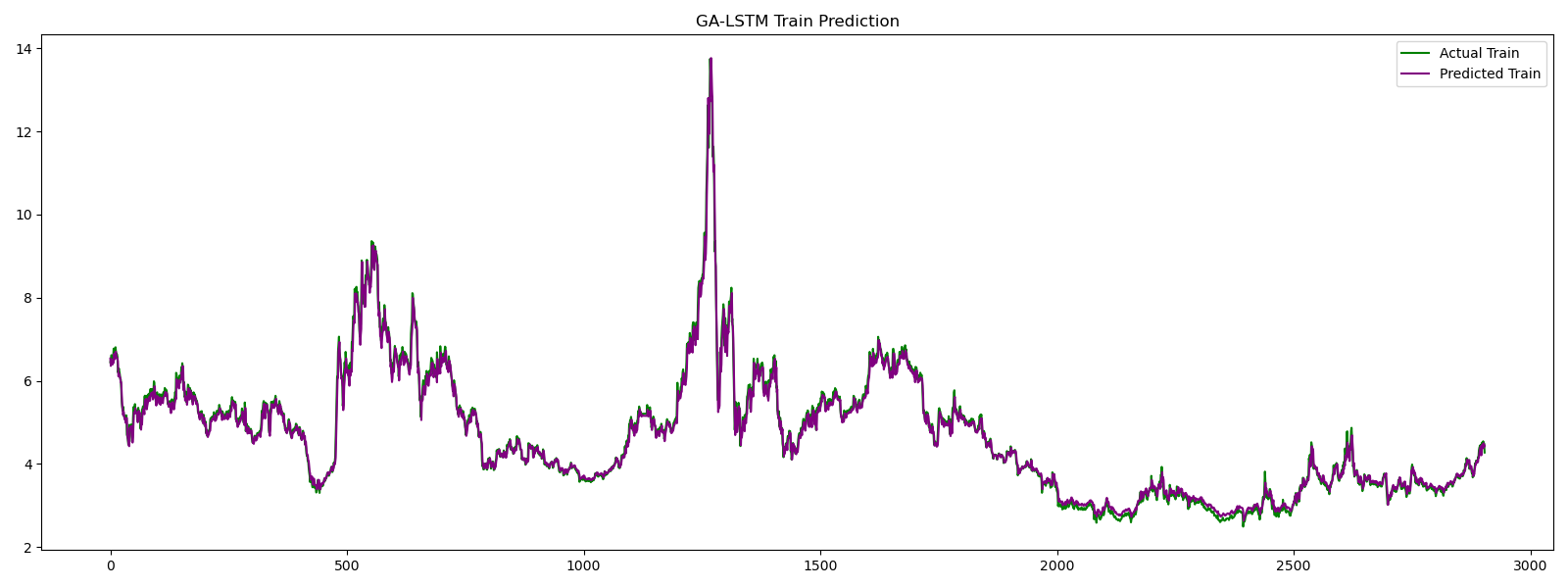

Supplement: S1 File — (ZIP) [file pone.0330324.s001.zip › Paper Model/V V/V V-600300/GA-WOA-LSTM/figures/GA_LSTM_train_fit_plot.png]

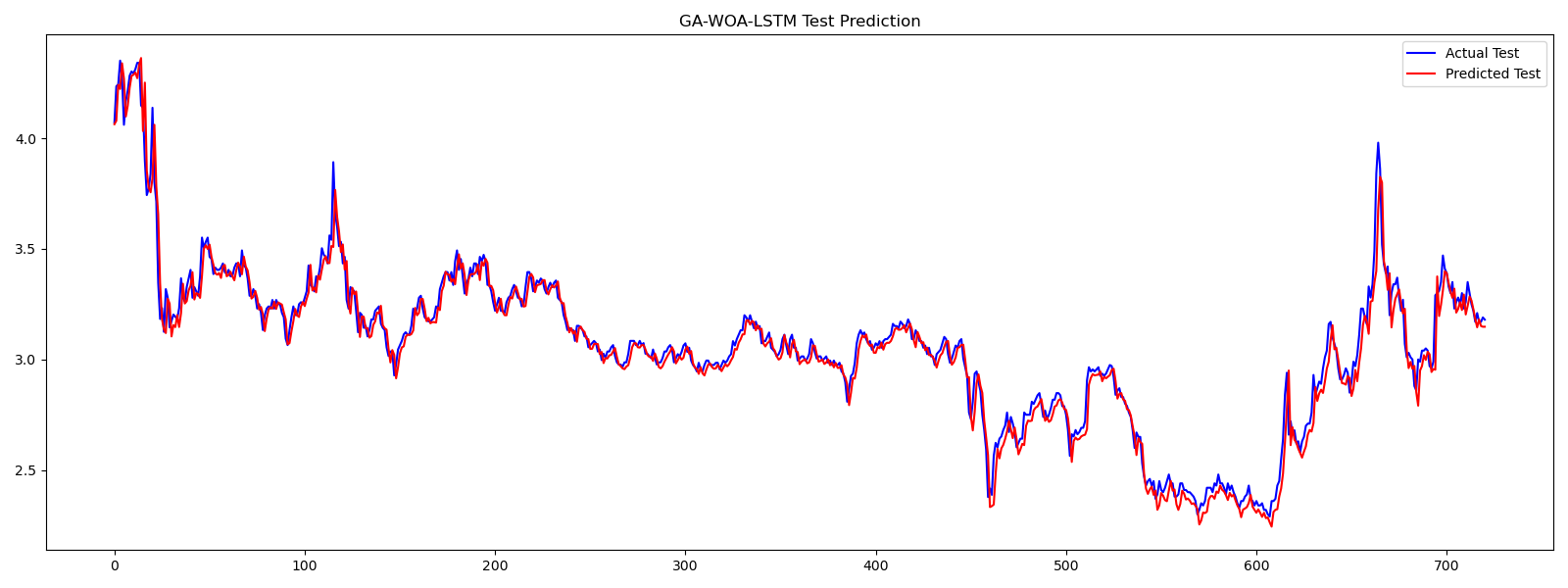

Supplement: S1 File — (ZIP) [file pone.0330324.s001.zip › Paper Model/V V/V V-600300/GA-WOA-LSTM/figures/GA_WOA_LSTM_test_fit_plot.png]

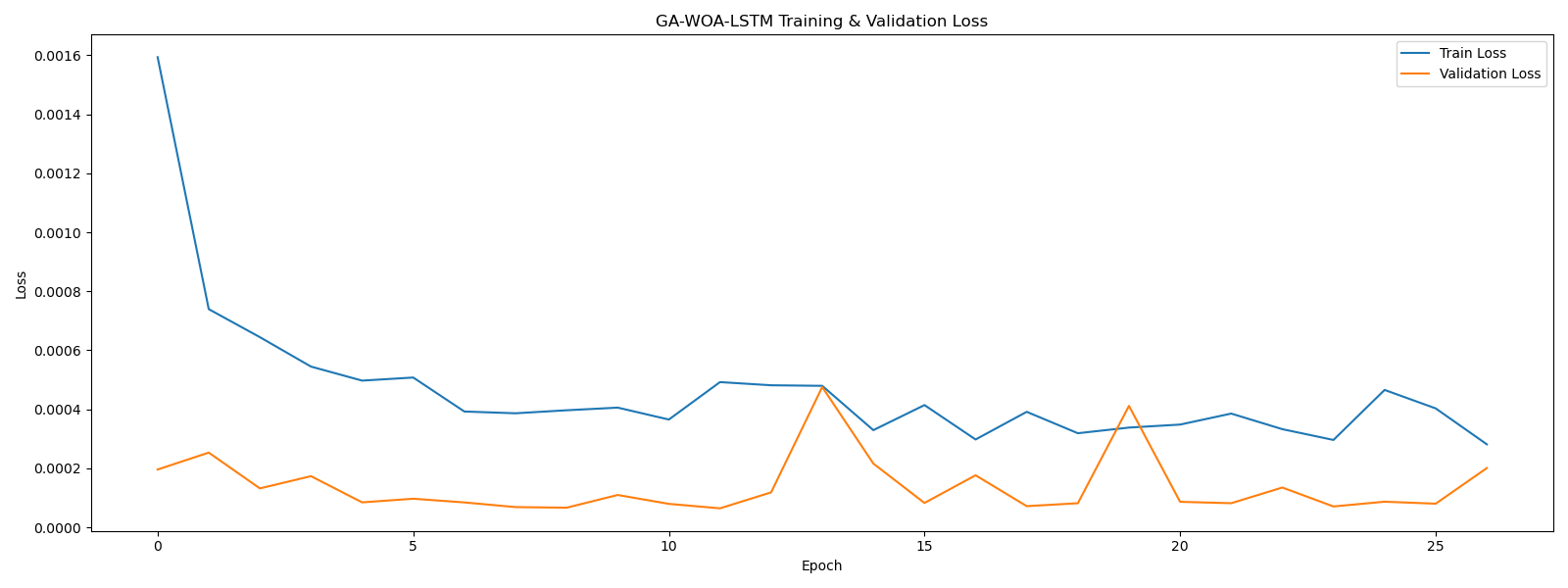

Supplement: S1 File — (ZIP) [file pone.0330324.s001.zip › Paper Model/V V/V V-600300/GA-WOA-LSTM/figures/GA_WOA_LSTM_training_loss.png]

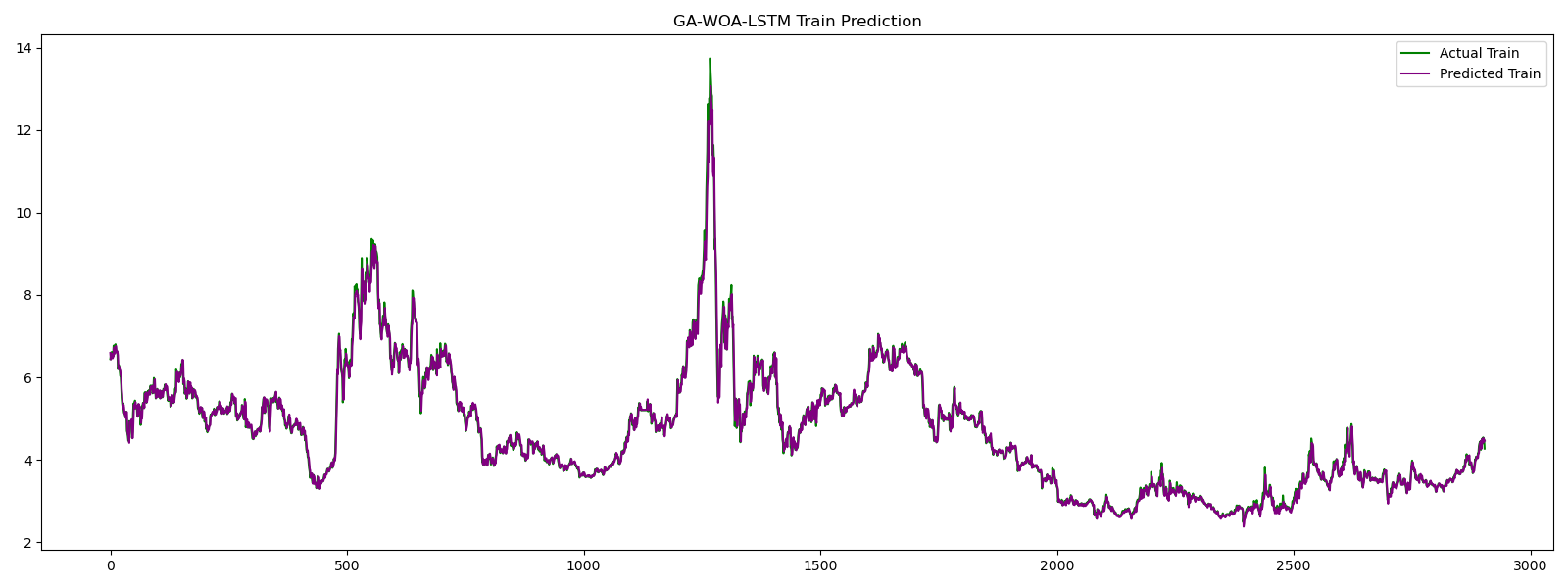

Supplement: S1 File — (ZIP) [file pone.0330324.s001.zip › Paper Model/V V/V V-600300/GA-WOA-LSTM/figures/GA_WOA_LSTM_train_fit_plot.png]

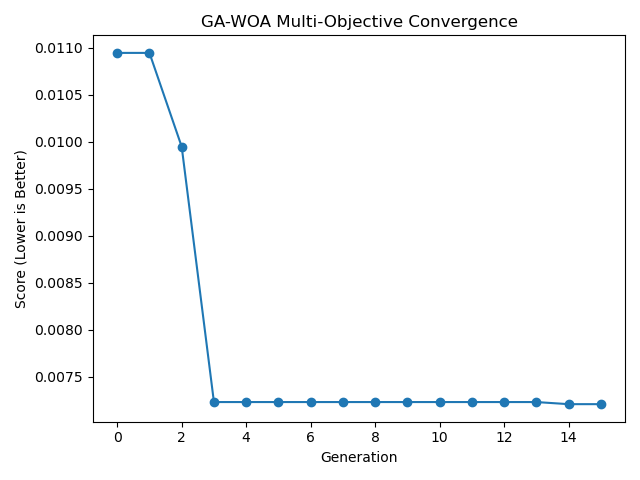

Supplement: S1 File — (ZIP) [file pone.0330324.s001.zip › Paper Model/V V/V V-600300/GA-WOA-LSTM/figures/GA_WOA_multiobj_convergence_curve.png]

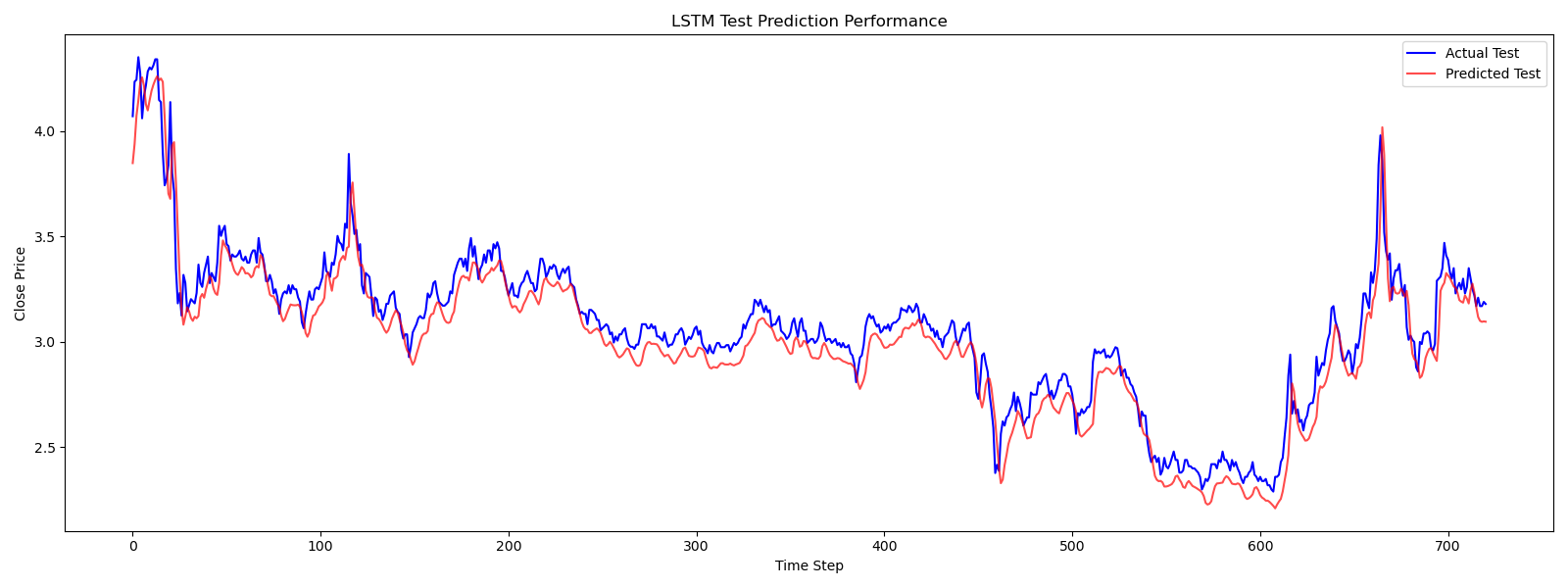

Supplement: S1 File — (ZIP) [file pone.0330324.s001.zip › Paper Model/V V/V V-600300/GA-WOA-LSTM/figures/LSTM_test_fit_plot.png]

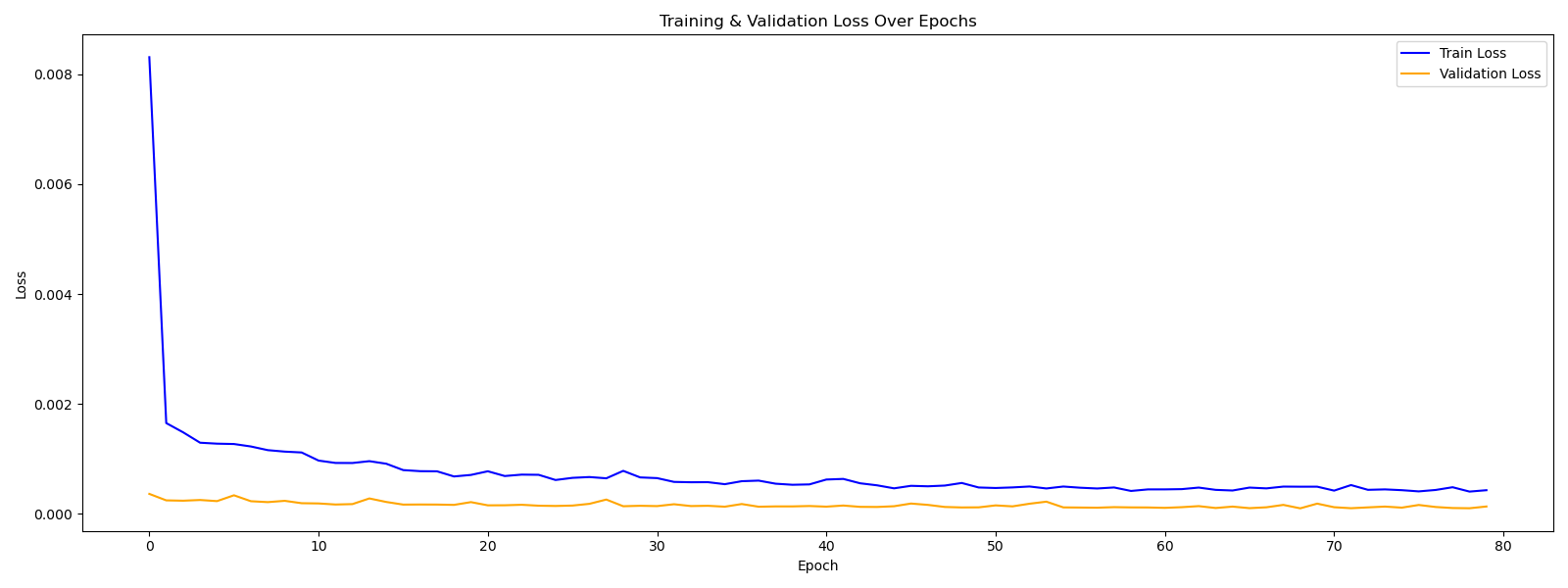

Supplement: S1 File — (ZIP) [file pone.0330324.s001.zip › Paper Model/V V/V V-600300/GA-WOA-LSTM/figures/LSTM_training_loss.png]

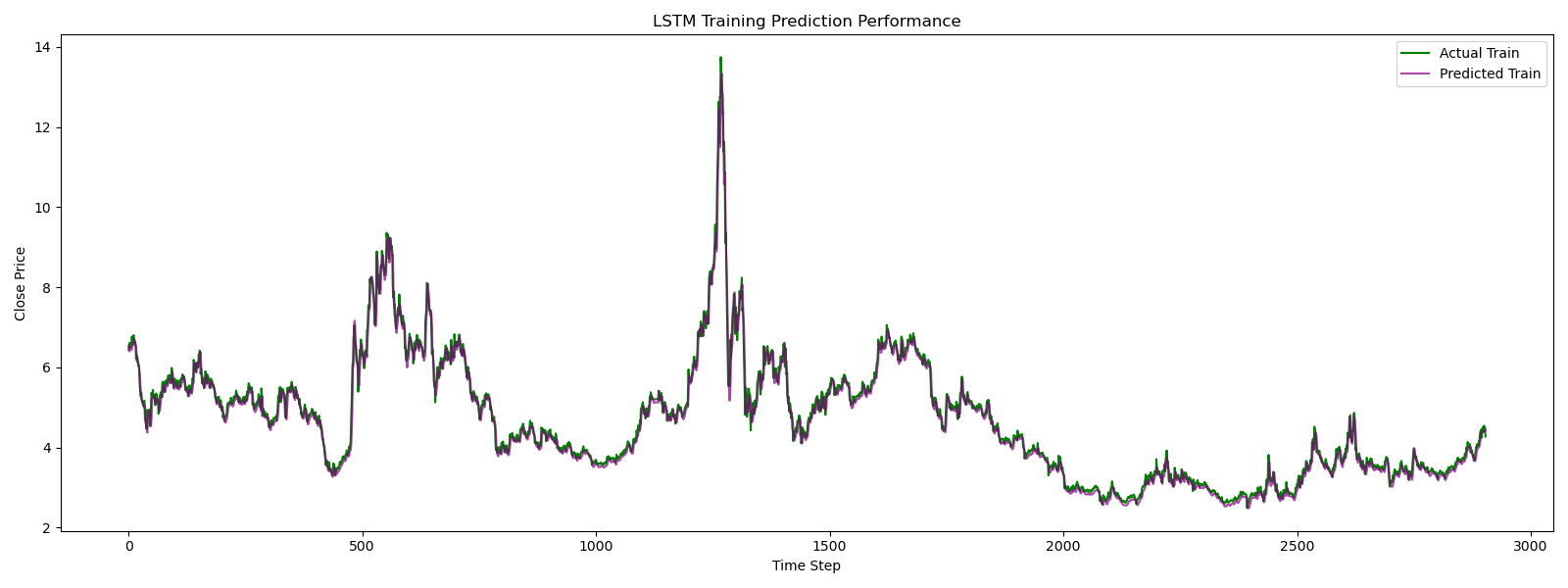

Supplement: S1 File — (ZIP) [file pone.0330324.s001.zip › Paper Model/V V/V V-600300/GA-WOA-LSTM/figures/LSTM_train_fit_plot.png]

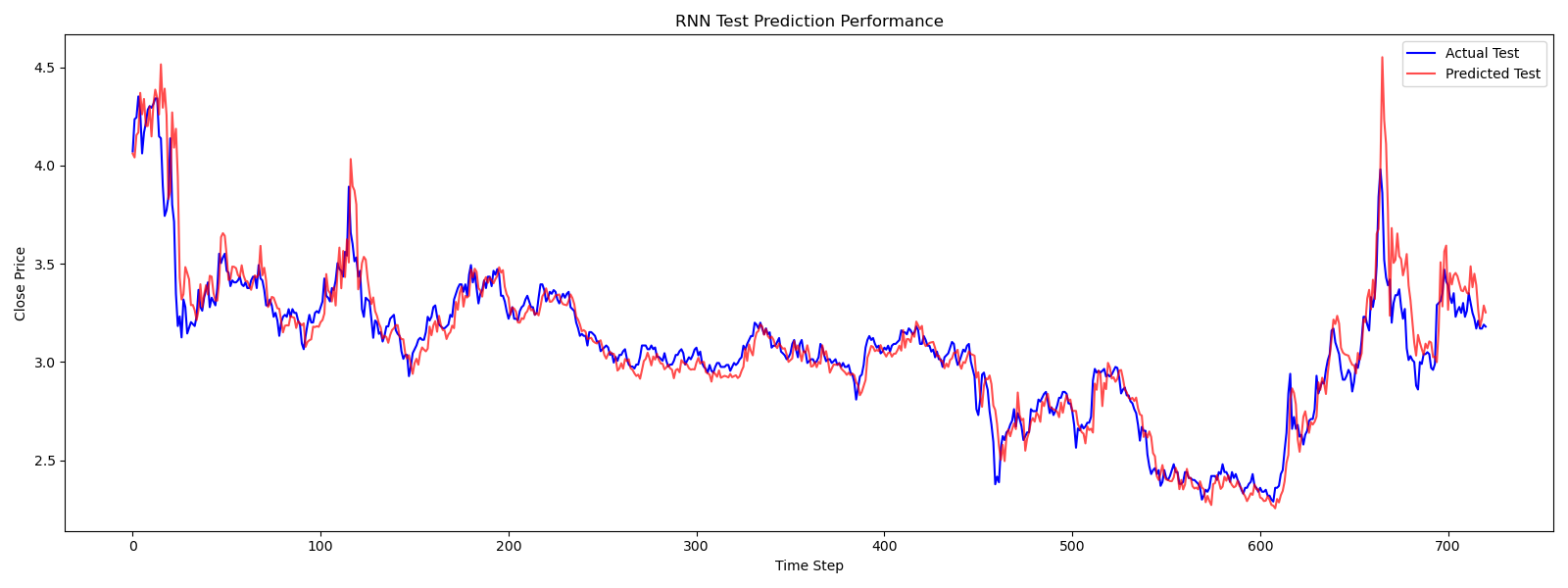

Supplement: S1 File — (ZIP) [file pone.0330324.s001.zip › Paper Model/V V/V V-600300/GA-WOA-LSTM/figures/RNN_test_fit_plot.png]

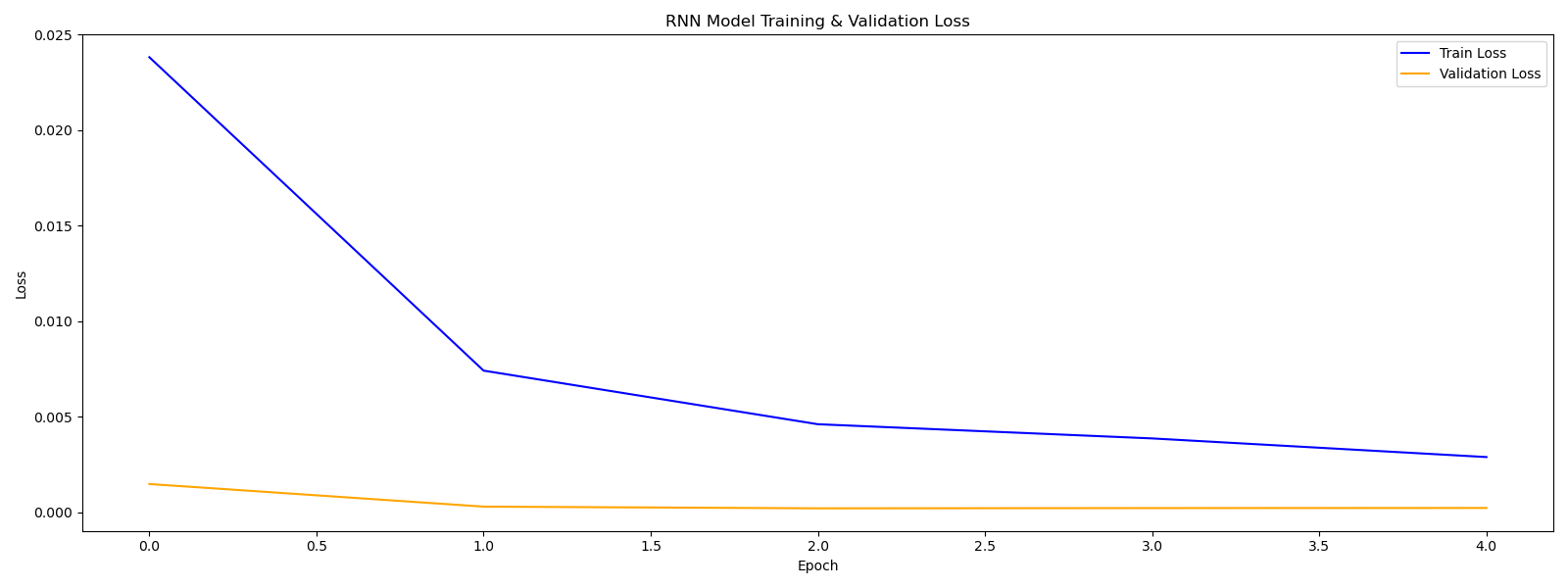

Supplement: S1 File — (ZIP) [file pone.0330324.s001.zip › Paper Model/V V/V V-600300/GA-WOA-LSTM/figures/RNN_training_loss.png]

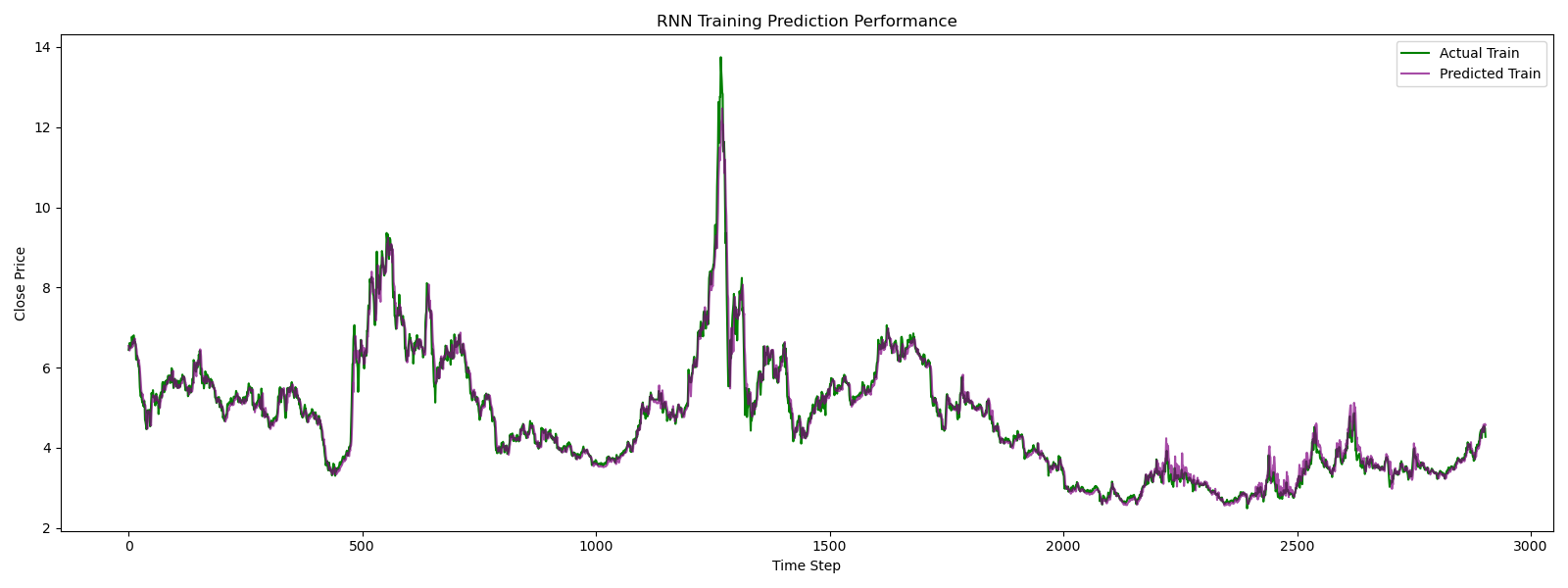

Supplement: S1 File — (ZIP) [file pone.0330324.s001.zip › Paper Model/V V/V V-600300/GA-WOA-LSTM/figures/RNN_train_fit_plot.png]

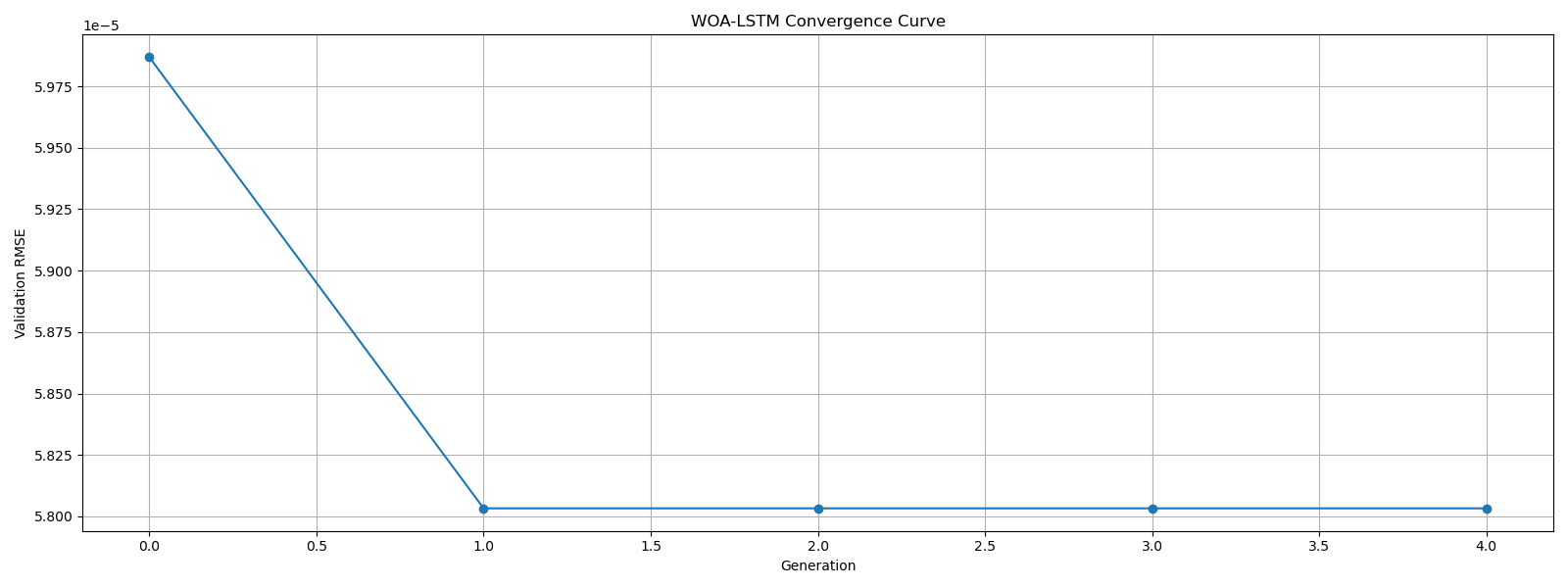

Supplement: S1 File — (ZIP) [file pone.0330324.s001.zip › Paper Model/V V/V V-600300/GA-WOA-LSTM/figures/WOA_LSTM_convergence_curve.png]

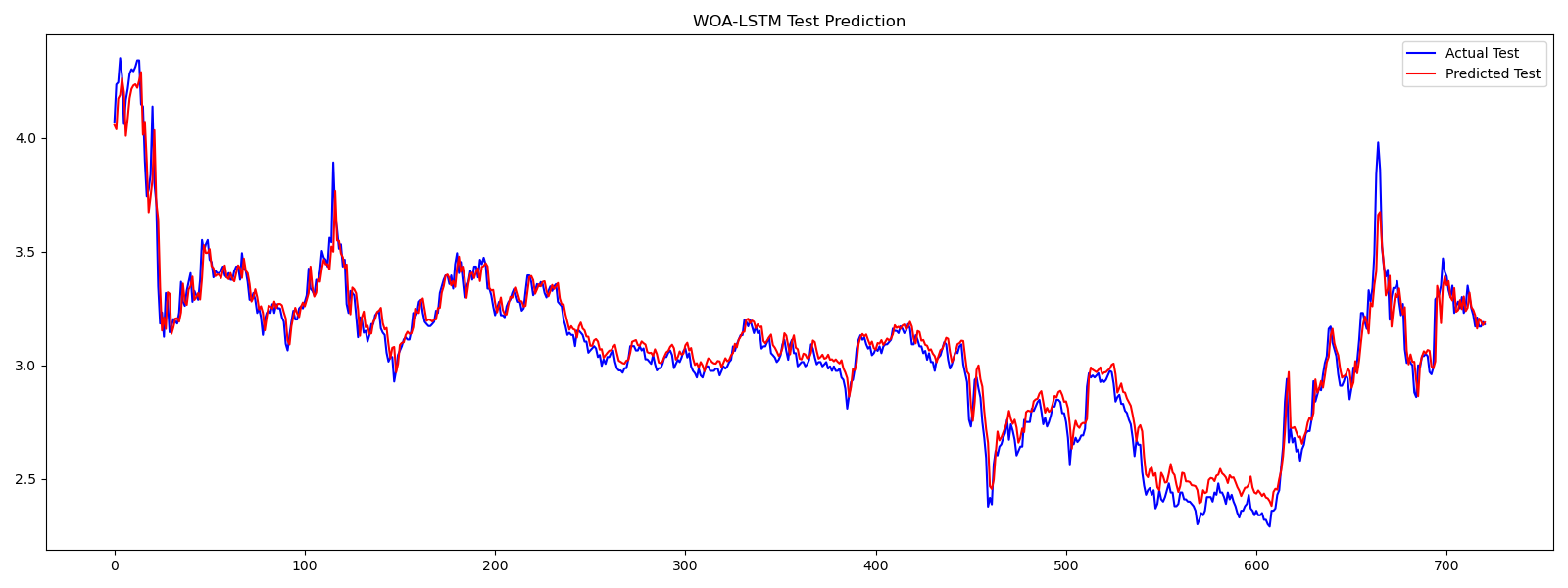

Supplement: S1 File — (ZIP) [file pone.0330324.s001.zip › Paper Model/V V/V V-600300/GA-WOA-LSTM/figures/WOA_LSTM_test_fit_plot.png]

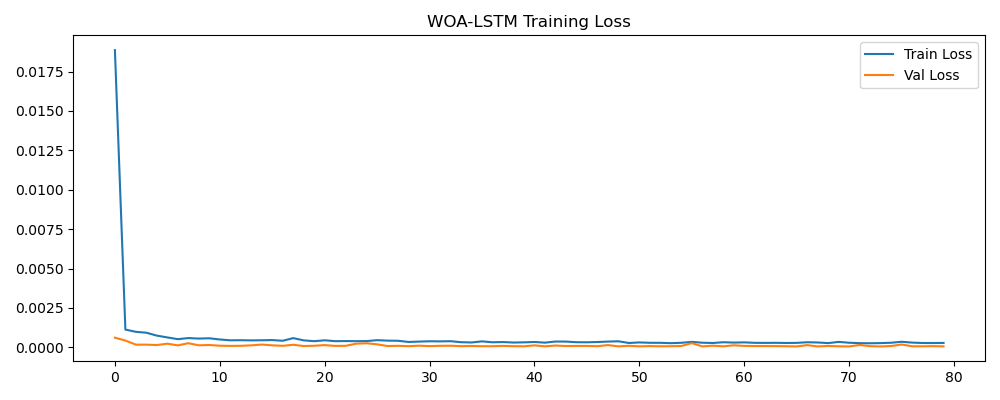

Supplement: S1 File — (ZIP) [file pone.0330324.s001.zip › Paper Model/V V/V V-600300/GA-WOA-LSTM/figures/WOA_LSTM_training_loss.png]

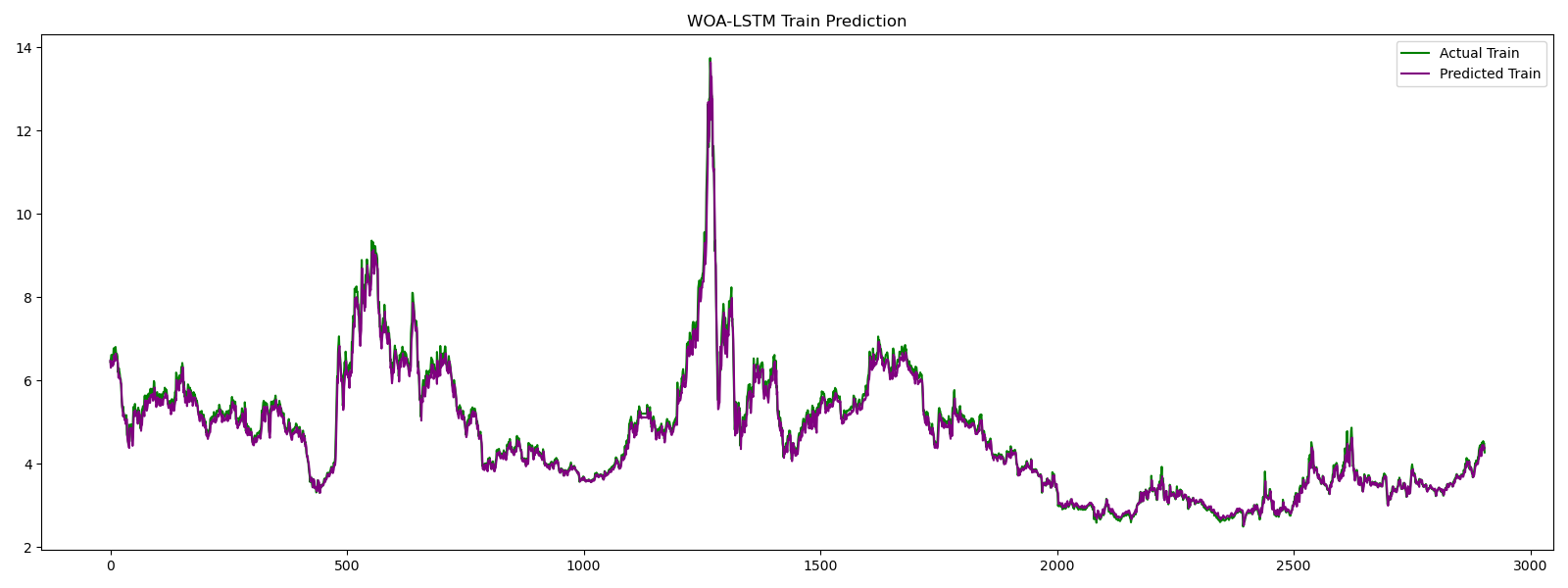

Supplement: S1 File — (ZIP) [file pone.0330324.s001.zip › Paper Model/V V/V V-600300/GA-WOA-LSTM/figures/WOA_LSTM_train_fit_plot.png]

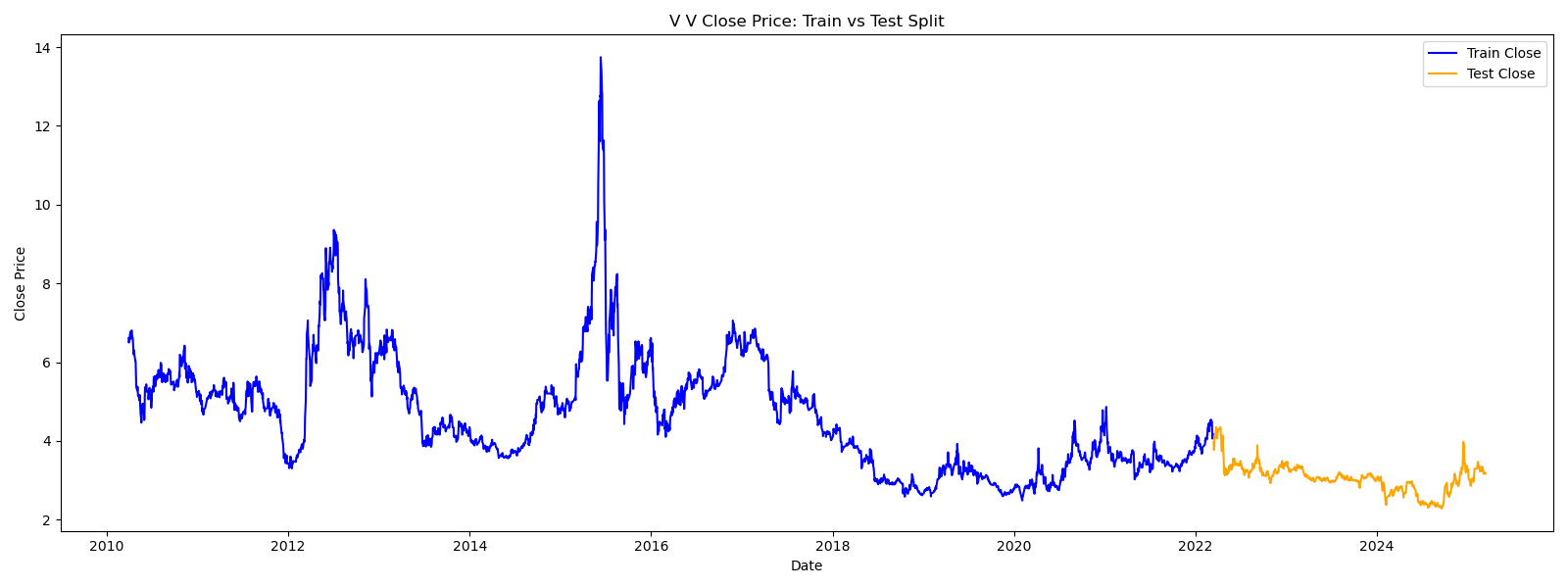

Supplement: S1 File — (ZIP) [file pone.0330324.s001.zip › Paper Model/V V/V V-600300/GA-WOA-LSTM/processed_data/close_price_split.png]
