# Supplementary material for: Stock market forecasting research based on GA-WOA-LSTM
Source: PLoS One. 2025 Aug 27;20(8):e0330324. doi: 10.1371/journal.pone.0330324 (PMC12385415; doi:10.1371/journal.pone.0330324)
Supplement: Attachment — Submitted filename: Response to Reviewers.docx [file pone.0330324.s003.docx]

Reviewer #1

Comment 1:The introduction is quite brief. It could be rewritten or added a new section titled 'Literature Review,' where more previous research could be discussed.

Response:Thank you for your review and suggestions on our paper. We have revised the introduction section you mentioned as too brief. In the new version, we rewrite the introduction section, we explicitly address the shortcomings of existing methods and illustrate the innovation and contribution of our research in this context.

Comment 2:The absence of a discussion section is clearly noticeable. Adding this section would help highlight the strengths of the proposed method.

Response:We understand the lack of the discussion section you mentioned and have added this section to the revision. In the new version, we discuss the findings in detail, especially the strengths and limitations of our proposed GA-WOA-LSTM approach. In the Discussion section, we analyze the experimental results in depth and compare them with the existing methods. We focus on highlighting the advantages of our method, such as prediction accuracy, stability of the model, and potential in practical applications.

Comment 3:The advantages of the proposed method over traditional approaches should be elaborated in greater detail. Additionally, the weaknesses of traditional methods should be discussed, and it should be clearly explained how the proposed method overcomes these limitations.

Response:We added a part of the revision to discuss the limitations of traditional methods. Specifically, traditional methods usually have problems such as failure to effectively handle large-scale data, low prediction accuracy, and poor adaptability to market dynamics. We explain in detail how GA-WOA-LSTM can overcome these problems by introducing optimization algorithms (such as genetic algorithms and whale optimization algorithms) and improve the predictive power of the model.

Comment 4:Include a data analysis framework in the section presenting the numerical example to provide a more structured approach for demonstrating how the data supports the study's findings.

Response:We introduced a more structured data analysis framework in the numerical examples section. Specifically, we first provide an overview of the data sources and preprocessing methods, and then detail the data analysis process, including each step of the feature selection, model training, and prediction process. In this way, we clearly show how the data can be used to support our findings.

Reviewer #2

Comment 1:The introduction of the research background is insufficient.

Response:Thank you for your review and valuable suggestions. In the revision, the gaps in the existing studies and the innovation of our research are clearly identified. And further explain the advantages of our GA-WOA-LSTM model in this context.

Comment 2:Thank you for your review and valuable suggestions. In the revision, the gaps in the existing studies and the innovation of our research are clearly identified. And further explain the advantages of our GA-WOA-LSTM model in this context.

Comment 3:How does the GA algorithm perform feature selection?

Response:In this paper, we propose a stock market prediction model based on GA-WOA-LSTM, and the innovation of this method is to optimize the LSTM model by combining genetic algorithm and whale optimization algorithm, thus improving the accuracy and robustness of the model in stock market prediction. Compared with conventional LSTM or other prediction models, our method is better able to adapt to complex nonlinear data relationships and overcome the limitations faced by conventional methods in the optimization process. Furthermore, we also validate the effectiveness of this model in practice and compare it with other existing models, demonstrating the superior performance of our method in multiple experimental settings.

Comment 4: What are the LSTM parameters optimized by WOA? How to set the upper and lower bounds of the optimization parameters? How to set the parameters of the comparison algorithms?

Response:In this study, we used the whale optimization algorithm (WOA) to optimize the hyperparameters of the LSTM model. Specifically, the LSTM parameters optimized for the WOA include:Number of neural units in the LSTM and learning rate.Meanwhile, we set the optimized upper and lower bounds for each LSTM hyperparameter
