# Supplementary material for: Stock market forecasting research based on GA-WOA-LSTM
Source: PLoS One. 2025 Aug 27;20(8):e0330324. doi: 10.1371/journal.pone.0330324 (PMC12385415; doi:10.1371/journal.pone.0330324)
Supplement: Attachment — Submitted filename: Response_to_Reviewers_auresp_2.docx [file pone.0330324.s004.docx]

Reviewer #2

Comment 1and 3:

We sincerely thank you for pointing out the inconsistency between the abstract and the main text regarding the terminology and methodological descriptions. We acknowledge that this was a lapse in our wording.

In this study, the Genetic Algorithm (GA) was not used for feature selection. Instead, GA was employed to perform global optimization of the LSTM model’s hyperparameters, including the number of neurons, dropout rate, learning rate, and batch size, rather than optimizing high-dimensional feature spaces. The search space of GA was explicitly defined by setting upper and lower bounds for each hyperparameter, and the optimization objective was to minimize the mean squared error (MSE) in cross-validation. The Whale Optimization Algorithm (WOA) was subsequently applied to locally fine-tune the initial solutions obtained from GA, thereby improving the overall model performance. The combination of these two optimization strategies effectively integrates global exploration with local exploitation.

To avoid potential misunderstanding, we have carefully revised and unified the abstract to ensure consistency with the main text, and to accurately reflect the respective roles of GA and WOA within the hybrid optimization framework. This revision only concerns the textual expression and does not affect the methodology or experimental conclusions of the paper.

Once again, we sincerely thank you for the thorough review and insightful suggestions, which have helped us further improve the clarity and academic rigor of the manuscript.

Comment 2:We sincerely thank you for identifying the inconsistencies between the abstract and the main text regarding the role of the Genetic Algorithm (GA). We acknowledge that the original abstract incorrectly described GA as being used for feature selection and data preprocessing, which does not reflect the actual methodology applied in this study. In our work, GA is not used for feature selection or to process high-dimensional feature spaces. Instead, it is employed to conduct global optimization of the LSTM model’s hyperparameters, including the number of neurons, dropout rate, learning rate, and batch size. The optimization objective is to minimize the mean squared error (MSE) based on cross-validation. To enhance the quality of the solutions found by GA, we integrate the Whale Optimization Algorithm (WOA), which is used to perform local fine-tuning of the best hyperparameter configurations found by GA. This hybrid approach effectively combines the global search capability of GA with the local exploitation strength of WOA.

In response to your request for more details on how GA and WOA are combined and how their experimental parameters are configured, we have provided additional clarifications in the revised manuscript. Specifically, we employ a two-stage optimization framework in which GA first explores the hyperparameter space globally, and WOA subsequently refines the best individuals generated by GA. The GA uses a population size of 20 and runs for 15 generations, with tournament selection , mean-based crossover, and an adaptive mutation rate that linearly decreases from 0.5 to 0.1. The WOA uses the same population size and operates with a dynamically increasing fusion ratio—from 30% (favoring GA) to 70% (favoring WOA)—to balance exploration and exploitation. The convergence control parameter a decreases linearly from 2 to 0 during optimization, and three types of search strategies (encircling, spiral, and random search) are randomly applied to each individual.

All experiments were conducted under a consistent data partitioning scheme, and each training run executed a complete GA-WOA optimization cycle. During LSTM training, an early stopping mechanism was employed to prevent overfitting. These additions have been clearly reflected in the revised “Model Construction” section to ensure greater transparency and reproducibility. We sincerely appreciate your insightful comments, which have significantly contributed to the integrity and academic rigor of this work

Comment 4:We sincerely appreciate your suggestion regarding the use of multiple datasets. We fully agree that testing the model on diverse datasets can further validate its robustness and generalizability. In response to your comment, we have conducted additional experiments on multiple datasets and updated the revised manuscript accordingly.

Comment 5:Thank you very much for your valuable comment. We fully understand that clear and precise language is essential for effective scientific communication. In response to your suggestion, we have carefully revised the manuscript to improve its overall writing quality, including grammar, sentence structure, and clarity of expression.

Once again, we sincerely appreciate your thoughtful feedback, which has helped us enhance the quality and clarity of our manuscript.
