# Supplementary material for: Stock market forecasting research based on GA-WOA-LSTM
Source: PLoS One. 2025 Aug 27;20(8):e0330324. doi: 10.1371/journal.pone.0330324 (PMC12385415; doi:10.1371/journal.pone.0330324)
Supplement: Attachment — Submitted filename: Response_to_Reviewers_auresp_3.docx [file pone.0330324.s005.docx]

Reviewer #3

Comment :

This study explicitly employs a sliding window method for time series samples. Each input sample is a continuous feature sequence spanning window sizes of 8 days, with the target being the closing price on day T+1. This approach enables modeling non-independent time-dependent structures while completely avoiding randomization or independent sample assumptions. In our data processing module, we first sort the data chronologically and then perform partitioning. The partition strategy allocates the first 80% of trading days as the training set and the last 20% as the test set. Rather than conducting cross-validation, we extracted 10% of the training set as a validation set for evaluation. Predictions were made using only real input features from the test set without employing recursive prediction structures, representing standard sliding window forecasting. This process does not incorporate prediction feedback or cumulative error mechanisms, making it more suitable for assessing models 'performance in real-time short-term trend analysis.

Second, we introduced WOA (Weighted Opaque Ant) centered on the optimal individual from the current GA solution to implement fine-tuned perturbation searches. Inspired by humpback whales' coordinated hunting behavior, this process combines "surrounding prey" and "spiral attack" strategies to conduct multi-angle searches around the current optimal solution, aiming to uncover potentially better solutions within its local neighborhood. The algorithm employs update mechanisms such as D = |C·X* -X| and X(t+1) = X* -A·D, demonstrating WOA's capability to actively approach and deeply explore the neighborhood of optimal individuals in the solution space. Furthermore, we introduced three unique search strategies specific to WOA in the implementation: optimal solution-centered linear envelope perturbations, spiral trajectory approximation, and mutation perturbations that jump to other individual positions. In each WOA iteration, the execution ratio of these three strategies is controlled by a random variable p, ensuring local search accuracy while maintaining the possibility of escaping local optima, thereby enhancing overall convergence stability. To further strengthen WOA's dominance in the convergence phase, the algorithm dynamically adjusts the synergy ratio between GA and WOA across generations, implementing a weight mechanism that gradually shifts toward WOA (linearly increasing from 30% to 70% over iterations) to achieve a natural transition from global exploration to local development. Through these mechanisms, WOA is systematically integrated into the local fine-tuning module of this model, not only preserving diversity in solution space perturbations but also significantly improving convergence accuracy in later optimization stages and the robustness of LSTM prediction performance.

Once again, we sincerely appreciate your thoughtful feedback, which has helped us enhance the quality and clarity of our manuscript.
